# Network analysis of psychological problems in school‐attending students aged 6–16 years in China: A comparison between rural and urban areas

**DOI:** 10.1002/ped4.70056

**Published:** 2026-04-14

**Authors:** Lianjingyi Liang, Wanling Zhang, Ying Li, Yanyu Wang, Raymond C. K. Chan

**Affiliations:** ^1^ School of Psychology Shandong Second Medical University Weifang Shandong China; ^2^ Department of Psychosomatic Medicine Beijing Children's Hospital, Capital Medical University, National Center for Children's Health Beijing China; ^3^ Neuropsychology and Applied Cognitive Neuroscience Laboratory State Key Laboratory of Cognitive Science and Mental Health, Institute of Psychology, Chinese Academy of Sciences Beijing China; ^4^ Department of Psychology the University of Chinese Academy of Sciences Beijing China

**Keywords:** Child Behavior Checklist, China, Network Analysis, Rural, Urban

## Abstract

**Importance:**

Urbanization significantly shapes the psychological health of children and adolescents.

**Objective:**

To apply network analysis to explore psychological problems among students aged 6–16 years from rural and urban areas of China.

**Methods:**

Using data from a multi‐stage stratified epidemiological survey, we analyzed 19 711 students (9566 urban; 10 145 rural). Among them, 3003 had mental disorders. Screening was conducted using the Child Behavior Checklist (CBCL), followed by the Mini International Neuropsychiatric Interview for Children and Adolescents and psychiatrist interviews. Network analysis was used to compare rural and urban CBCL networks.

**Results:**

In the entire sample, social problems, thought problems, rule‐breaking behavior, and aggressive behavior were more pronounced in the rural group, while the urban group showed elevated somatic complaints. The rural and urban networks exhibited significant differences in edge weights (*M* = 0.186, *P* < 0.001), but showed no significant difference in global network strength (*S* = 1.608, *P* = 0.086). Among participants with mental disorders, withdrawn/depressed, social problems, thought problems, rule‐breaking behavior, and aggressive behavior were more prominent in the rural group, while the urban group showed more somatic complaints and aggressive behavior. The rural and urban networks exhibited significant differences in edge weights (*M* = 0.223, *P* = 0.001) but showed no significant difference in global network strength (*S* = 3.245, *P* = 0.358).

**Interpretation:**

Urbanization is a significant environmental determinant affecting youth mental health in China, linked to distinct psychological patterns. Rural students tended to show more emotional problems, whereas urban students demonstrated more behavioral issues.

## Graphical abstract

A network analysis of 19711 Chinese children and adolescents found that urbanization shapes mental health symptom patterns. Rural students showed more emotional and social problems, while urban students had more somatic complaints and aggressive behavior. Although symptom networks differed in structure, overall symptom connectivity was similar across rural and urban areas.

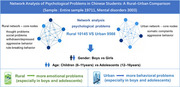

## INTRODUCTION

The effect of urbanization on mental health has gained growing attention, with research suggesting that urbanicity may be a risk factor for mental disorders.^1^ The differences between rural and urban areas contributed to significant mental health disparities.^2^ Urban and rural areas differ in economic relations, social structures, population density, and natural environments, encompassing factors that influence one's mental well‐being.^3^ In China, the disparity between rural and urban areas has been widening quickly, as the current average per capita household income in urban areas is three times higher than that in rural areas.^4^ National surveys in China indicated that 17.5% of school children and adolescents have mental health issues, with a higher prevalence of mental disorders in urban areas than in rural areas.^5^

Recently, the evolving trends of physical growth of children and adolescents in China have shown persistent disparities between urban and rural areas, although a notable overall improvement has been observed in physical growth over three decades.^6^ On the other hand, the effects of urbanicity on mental health in children and adolescents warrant further investigations. The fast‐paced economic reforms and urbanization that have occurred over the past three decades may intensify the effects of urbanicity on mental health.^7^ Many parents in rural areas moved to urban areas for economic opportunities, resulting in a growing number of “left‐behind children” who were under the care of grandparents.^8^ A meta‐analysis of 11 studies pooled data from 4621 migrant children and 5076 urban children, and found that migrant children exhibited poorer mental health than their urban counterparts.^9^ However, another study found that urban dwellers had a higher incidence of mental health issues related to the coronavirus disease 2019 (COVID‐19) pandemic.^10^ Therefore, a thorough investigation into the effects of urban‐rural disparity on mental health is needed for health policy planning. Targeted mental healthcare addressing the diverse circumstances across geographical settings would benefit from such knowledge.^9^

Regarding psychiatric symptoms, a cross‐sectional study recruited 372 migrant, 254 urban, and 268 rural children from various schools in Guangdong Province, China, and showed that migrant and rural children displayed higher scores in emotional symptoms, hyperactivity/inattention, and overall difficulties compared to their urban counterparts.^11^ Another study found that rural Chinese students exhibited more symptoms of anhedonia compared to their urban counterparts.^12^ In another survey that assessed 2400 college students in Beijing, no statistically significant difference was observed between the rural and the urban groups concerning measures of depressive symptoms and suicide ideation.^13^ Inconsistent findings in the extant literature call for more refined research on the possible urban‐rural disparities in psychopathological characteristics among children and adolescents.

As a powerful tool to study psychopathology, network analysis accounts for the complex interplay between different psychological problems.^14^ This method has been used in a previous study on the rural–urban disparity in mental health.^15^ Few network analysis studies have investigated the psychopathology of children and adolescents, and none have explored the disparity in psychopathological phenomena between rural and urban areas.

Therefore, the current study aimed to explore the effects of urban factors (urbanicity) and rural factors on the mental health of children and adolescents in China using network analysis. First, we sought to examine the distinct characteristics of psychopathology in children and adolescents in rural and urban settings based on the network perspective. Then, the whole sample was categorized into two groups, i.e., the healthy subgroup and the subgroup with mental disorders. Differences in psychological problems between rural and urban environments were examined in the subgroup analyses. We hypothesized that significant differences would exist in the psychopathology of Chinese children and adolescents based on rural and urban settings.

## METHODS

### Ethical approval

The study protocol was approved by the Ethics Committee of Anding Hospital, Capital Medical University, China (Project identification code: 2012BAI01B02). All procedures were conducted in accordance with the Declaration of Helsinki. Underage participants and their primary guardians provided written informed consent.

### Participants

Data in this research were collected from the first national epidemiological survey across China on children's mental disorders.^16^ This previous population‐based epidemiological survey comprised two phases.^10^ In the first phase, the Child Behavior Checklist (CBCL)^17^ was employed for screening, followed by the Mini International Neuropsychiatric Interview for Children and Adolescents (MINI‐KID) for diagnostic evaluation.^18^, ^19^ Some results of this national epidemiological survey have been published elsewhere.^10^, ^16^ In the present study, we acquired a part of this data from the authors,^16^ consisting of participants who had specific information regarding the residence (and thus clarified participants’ locations in either urban or rural areas), along with demographics including age, gender, ethnicity, family members, and other related information.

The resultant sample comprised 19 711 participants aged 6–16 years from one province of China, classified into children (aged 6–11 years) and adolescents (aged 12–16 years). The screening and recruitment processes of the original sample were conducted systematically and meticulously.^10^, ^16^ We excluded data from other provinces due to the following characteristics of this provincial dataset. First, the data from this province clearly indicated which school each participant was attending, and therefore provided valid and essential information to categorize the sample into urban and rural groups. Second, during the stratified sampling process in this province, random stratification was based on rural and urban distinctions. Third, the disparity between rural and urban areas in this province is the most pronounced compared with that in other regions in China.

Specifically, in this province, data collection was carried out across a wide geographic area, involving 40 primary and junior/senior high schools (21 primary schools and 19 junior/senior high schools). For primary schools, students from the first to the sixth grades were enrolled (aged approximately 6–12 years). For junior high schools, Year 1–3 students were involved (aged approximately 13–15 years). For senior high schools, only Year 1–2 students (aged approximately 15–16 years) were involved. Year 3 senior high school students were excluded because they were preparing for the National College Entrance Examination. In this study, 11 primary and 10 junior/senior high schools were categorized as rural, while the other 10 primary and 9 junior/senior high schools were classified as urban.

### Assessment procedure

#### Child Behavior Checklist

The observer‐rated CBCL, developed by Achenbach and colleagues, is a widely used instrument designed to assess emotional and behavioral problems in children. The Chinese version of the CBCL has been validated and demonstrated satisfactory reliability and validity.^20^ The CBCL contains 113 items, which are scored on a 3‐point Likert scale of 0 = “none”, 1 = “a little”, and 2 = “a lot” (For the 8 subscales of the CBCL and their abbreviations are listed in Table S1; the content of each CBCL item is detailed in Table S2). Higher total scores of the CBCL indicate higher severity of the child's emotional and behavioral issues. In this study, the Cronbach's alpha for the scale was 0.949. In stage 1, the CBCL was adopted as the primary screening tool, indexing both the total score and the eight subscales.^21^

#### MINI‐KID and Diagnostic and Statistical Manual of Mental Disorders, Fourth Edition interview

In stage 2, 50 trained and qualified psychiatrists conducted two types of interviews with both parents and children. Firstly, the semi‐structured MINI‐KID^19^ was administered, and then the Diagnostic and Statistical Manual of Mental Disorders, Fourth Edition (DSM‐IV) interview was also applied. Clinicians used the MINI‐KID results as references when administering the DSM‐IV interview to aid the final diagnosis. The Chinese version of the MINI‐KID showed good psychometric properties,^18^ and has been frequently used in formal psychiatric evaluations. Excellent consistency in ratings amongst all clinicians was observed, with an intraclass correlation coefficient of 0.85.^22^

### Data analysis

#### Descriptive analysis

Descriptive analysis was conducted on demographic data and clinical measurements utilizing SPSS (Version 26.0). To ascertain the differences in age and gender between the whole sample and the validated subgroups, we employed the independent‐sample *t*‐tests and Chi‐square tests. For data not normally distributed, the Mann‐Whitney *U* test was utilized for comparisons. Accordingly, quantitative data with a normal distribution were described using the mean ± standard deviation, while data with a non‐normal distribution were described using the median (interquartile range). Categorical data are presented as numbers and percentages (*n*, %).

#### Network analysis based on CBCL

For the whole sample (*n* = 19 711) and the subsample of participants with diagnosed mental disorders (*n* = 3003), nodes (Cn) representing each item of the CBCL were calculated to construct the rural and urban networks separately. Then the network comparison test (NCT) was employed, contrasting the established rural‐urban networks to determine whether differences existed between their global and edge strengths.

The “qgraph” package in R (Version 4.2.2) was loaded to construct the networks and determine the centrality indexes. Within each network, nodes representing each item in the CBCL were classified by different colors based on the 8‐factor model. Edges between nodes signified the partial correlation between two nodes, with blue edges indicating positive correlations. The thickness of an edge represented the strength of the correlational coefficient. We set a minimum weight threshold of 0.1 for each edge to provide a clear visualization of the network.

Specifically, the EBIC “glasso” function from the “qgraph” R package derived two networks (rural versus urban) from a dataset of 19 711 participants using the Gaussian Graphical Model (GGM).^23^ In this study, the urban subgroup had 9566 participants (48.5%) and the rural subgroup 10 145 (51.5%). The Least Absolute Shrinkage and Selection Operator (LASSO) algorithm shrank minor edges to zero for a more interpretable and consistent illustration. The GGM tuning parameter was set at the advised value of 0.5.^24^ Within the illustrated network, red and blue edges depicted negative and positive partial correlations. The magnitude of the correlation was reflected by the edge thickness.

Subsequently, the “bootnet” function in R was employed to gauge the network's stability and accuracy.^25^ The exactness of edge weights was assessed via 95% confidence intervals (CIs) computed through bootstrapping techniques. A narrower CI suggests more precise estimations for edge weights and centrality indicators. The stability of the centrality measures was evaluated by calculating the correlation stability coefficient (CS‐coefficient) through a case‐dropping bootstrap technique. Ideally, CS‐coefficient, indicating how much data can be omitted, should surpass 0.50, but never fall below 0.25.^23^

Given established critiques regarding the conceptual and practical limitations of traditional centrality measures in psychopathological networks,^26^, ^27^ the current study adopted expected influence as its primary centrality index. Expected influence has been proposed as a more accurate measure for cross‐sectional networks, as it accounts for both positive and negative edge weights and better predicts a node's influence on others.^28^, ^29^ In line with the common interpretation of centrality metrics in psychopathology, nodes with the highest expected influence values were considered the most central and potent risk factors in the network, whereas nodes with the lowest values were viewed as potential protective factors. Thus, higher expected influence reflects a node's stronger potential for activation interplay within the symptom network.^30^

Finally, we employed the NCT in R for statistical analysis. First, we examined the interaction effect of age group (children vs. adolescents) and residential setting (rural vs. urban) on the psychological network structure. Second, with age controlled as a covariate, we analyzed the differences in psychological network structure by gender and residential setting (rural vs. urban). The NCT leverages a permutation test to scrutinize the uniformity in global strength (the aggregate of edge weights) and architecture across two distinct networks.^31^

## RESULTS

### Sample characteristics

A total of 19 711 participants were included in this study. The entire sample had a mean age of 11.7 ± 3.0 years and comprised 9938 (50.4%) boys and 9773 girls (49.6%). The age distribution was treated as approximately normal for parametric tests, as its skewness (−0.295) and kurtosis (−1.051) fell within acceptable limits,^32^ despite a significant Kolmogorov‐Smirnov test (*P* < 0.001) attributable to the large sample size. As detailed in Table S3, significant demographic differences were observed between the rural (*n* = 10 145, 51.5%) and the urban (*n* = 9566, 48.5%) subsamples. Participants from the rural subsample (12.0 ± 2.8 years) were significantly older than those from the urban subsample (11.4 ± 3.2 years, *P* < 0.001). The gender distribution also differed significantly (*P* < 0.001), while no other demographic variables showed significant differences. Based on the diagnostic assessment, 3003 (15.2%) participants met the criteria for at least one mental disorder, while the remaining 16 708 (84.8%) were classified as healthy.

### Comparing rural and urban groups based on the CBCL

The CBCL total score for the entire sample was non‐normally distributed, with a median of 25 (13, 39). The median CBCL total scores were 25 (13, 39) for the urban subgroup and 25 (14, 39) for the rural subgroup. For participants with mental disorders and healthy participants, the median CBCL total scores were 52 (41, 66) and 21 (12, 33), respectively (Table S4).

Given that our dataset did not manifest as a normal distribution, we used the Mann‐Whitney *U* test to compare the eight subscale scores between the urban and rural subgroups. Results showed that the urban subgroup scored higher on the “social problems” and “rule‐breaking behavior” subscales (*P* < 0.001), but lower on the “anxious/depressed,” “withdrawn/depressed,” “somatic complaints,” and “attention problems” subscales than the rural subgroup (*P* < 0.001) (Table 1).

**TABLE 1:** Comparison of urban‐rural subgroups in different dimensions

Scoring method	Dimension	Urban (*n* = 9 566)	Rural (*n* = 10 145)	*Z*	*P‐*value
CBCL total score	–	25 (13, 39)	25 (14, 39)	−0.756	0.450
CBCL 8‐structure‐syndrome	Anxious/depressed	2 (0, 4)	2 (0, 4)	−2.619	0.009
	Withdrawn/depressed	2 (1, 4)	2 (1, 4)	−2.572	0.010
	Somatic complaints	2 (1, 4)	2 (1, 5)	−6.781	<0.001
	Social problems	2 (1, 4)	2 (1, 4)	−2.724	0.006
	Thought problems	2 (0, 3)	2 (0, 3)	−1.621	0.105
	Attention problems	3 (1, 5)	3 (1, 5)	−2.872	0.004
	Rule‐breaking behavior	2 (1, 4)	2 (1, 4)	−2.351	0.019
	Aggressive behavior	5 (2, 8)	5 (2, 8)	−0.181	0.856
	Syndrome total score	22 (12, 35)	22 (12, 35)	−1.433	0.152

Data are presented as median (interquartile range).

CBCL, Child Behavior Checklist; The 8‐structure‐syndrome refers to the eight core syndromic scales of the CBCL: anxious/depressed, withdrawn/depressed, somatic complaints, social problems, thought problems, attention problems, rule‐breaking behavior, and aggressive behavior. *Z* values were derived from the Mann‐Whitney *U* test.

### Networks of the entire sample (*n* = 19 711)

**FIGURE 1:**
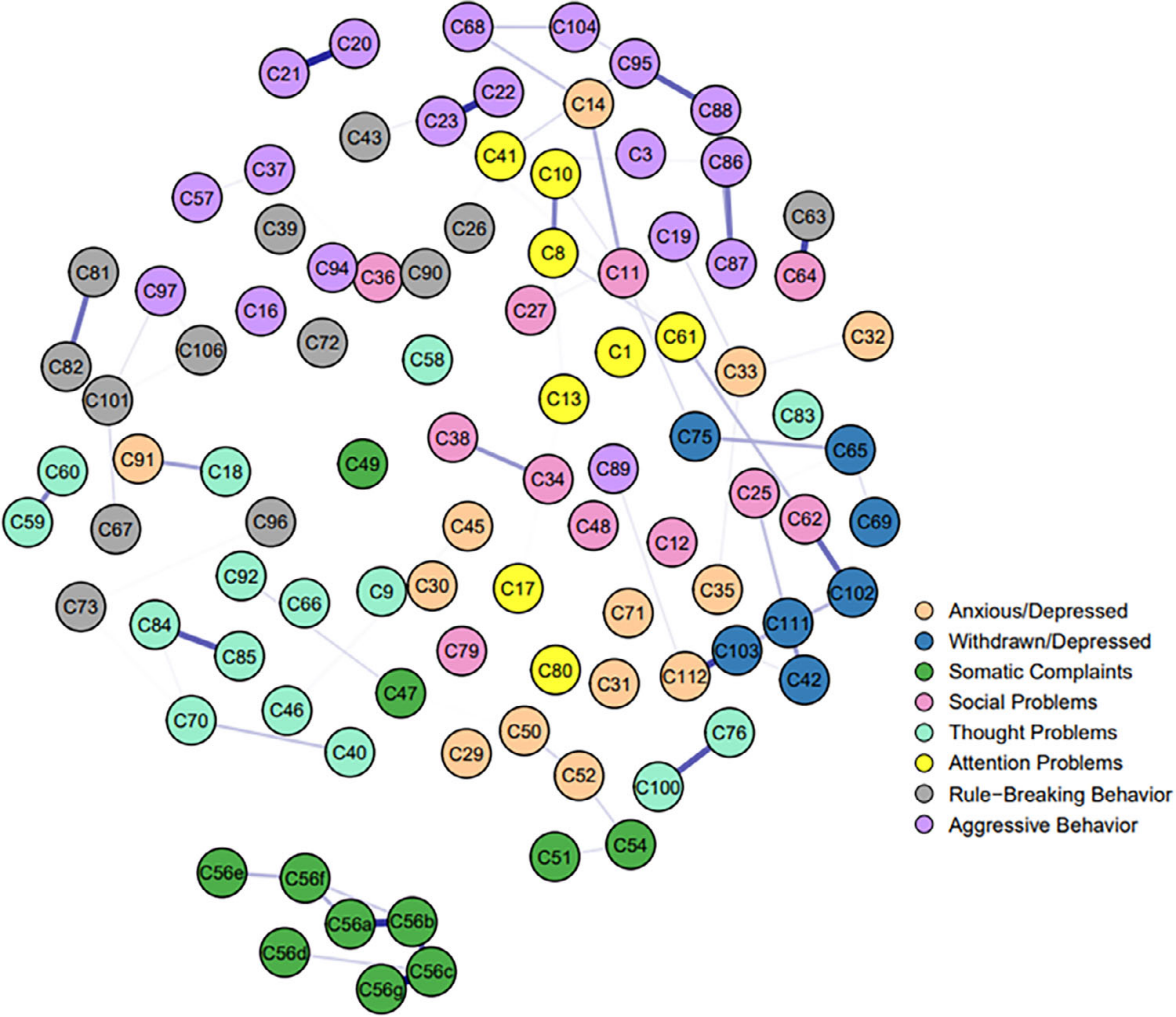
Networks of rural groups of the entire sample.

**FIGURE 2:**
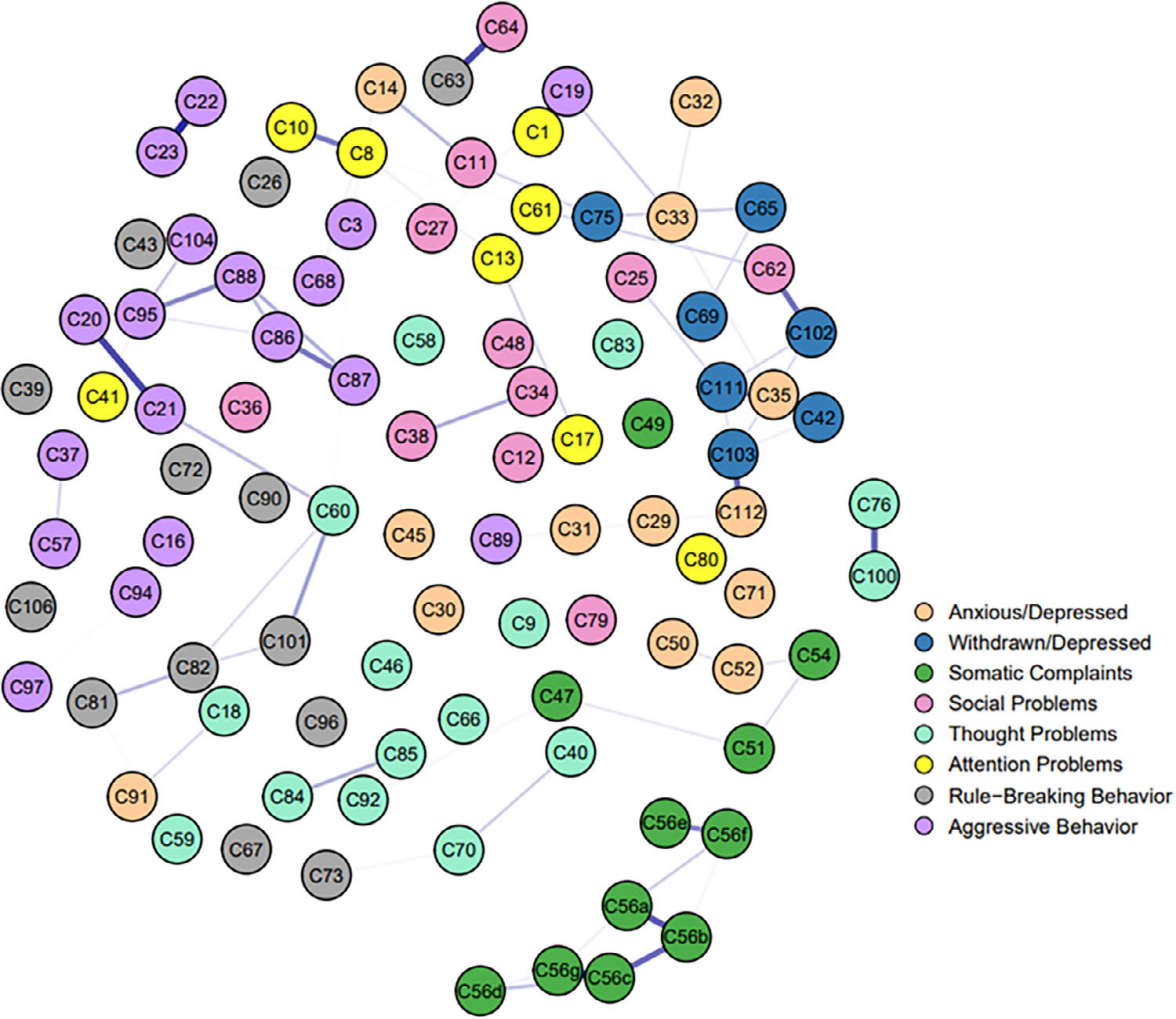
Networks of urban groups of the entire sample.

Based on the whole sample, we separately constructed two LASSO networks, one for the rural subsample (Figure 1), the other for the urban subsample (Figure 2). Both networks exhibited a sparse structure, with numerous weak or non‐significant edges between nodes across different dimensions. For instance, connections between items in the “anxious/depressed” and “aggressive behavior” syndromes were predominantly weak or absent, as were many edges within the “social problems” dimension, among others. Overall, in the two samples, the strongest weighted edge was from C56c (feeling sick) to C56g (vomiting), with partial correlation values in the rural and urban networks of 0.331 and 0.307, respectively. In addition, C20 (damaging one's own things) to C21 (damaging things at home or of others), C22 (disobedience at home) to C23 (disobedience at school), C63 (prefers to play with older children) to C64 (prefers to play with younger children), C76 (less sleep) to C100 (poor sleep quality) also exhibited strong partial correlations in both samples, with partial correlation values (rural/urban) of 0.294/0.264, 0.283/0.293, 0.262/0.264, 0.247/0.243, indicating good internal consistency.

In the rural sample, the node with the strongest strength was C11 (clinging to adults or excessively dependent), with a CS‐coefficient of 0.75. In the urban sample, the node with the strongest strength is C60 (excessive genital play), with a CS‐coefficient of 0.75. (See Figures S1–S6 for additional information).

In terms of the differences between the two samples, a connection was observed between C56e (skin rash or other skin issues) and C56f (abdominal pain or cramps) in the urban sample, whereas the connection was not apparent in the rural sample. In the rural sample, connections were observed between C59 (public genital play) and C60, C81 (stealing at home) and C82 (stealing outside), C84 (strange behavior) and C85 (strange thoughts), as well as between C88 (frequently angry) and C95 (temper tantrums or irritability), whereas these connections were not apparent in the urban sample.

The results of the NCT showed significant differences between the two networks in edge weights (*M* = 0.186, *P* < 0.001), but no significant difference in the global strength of the networks (*S* = 1.608, *P* = 0.086). When compared, 298 edges showed significant differences in edge weights, and 21 nodes showed significant differences in central strength between the two networks.

### Network analysis results of the sample with mental disorders (*n* = 3003)

**FIGURE 3:**
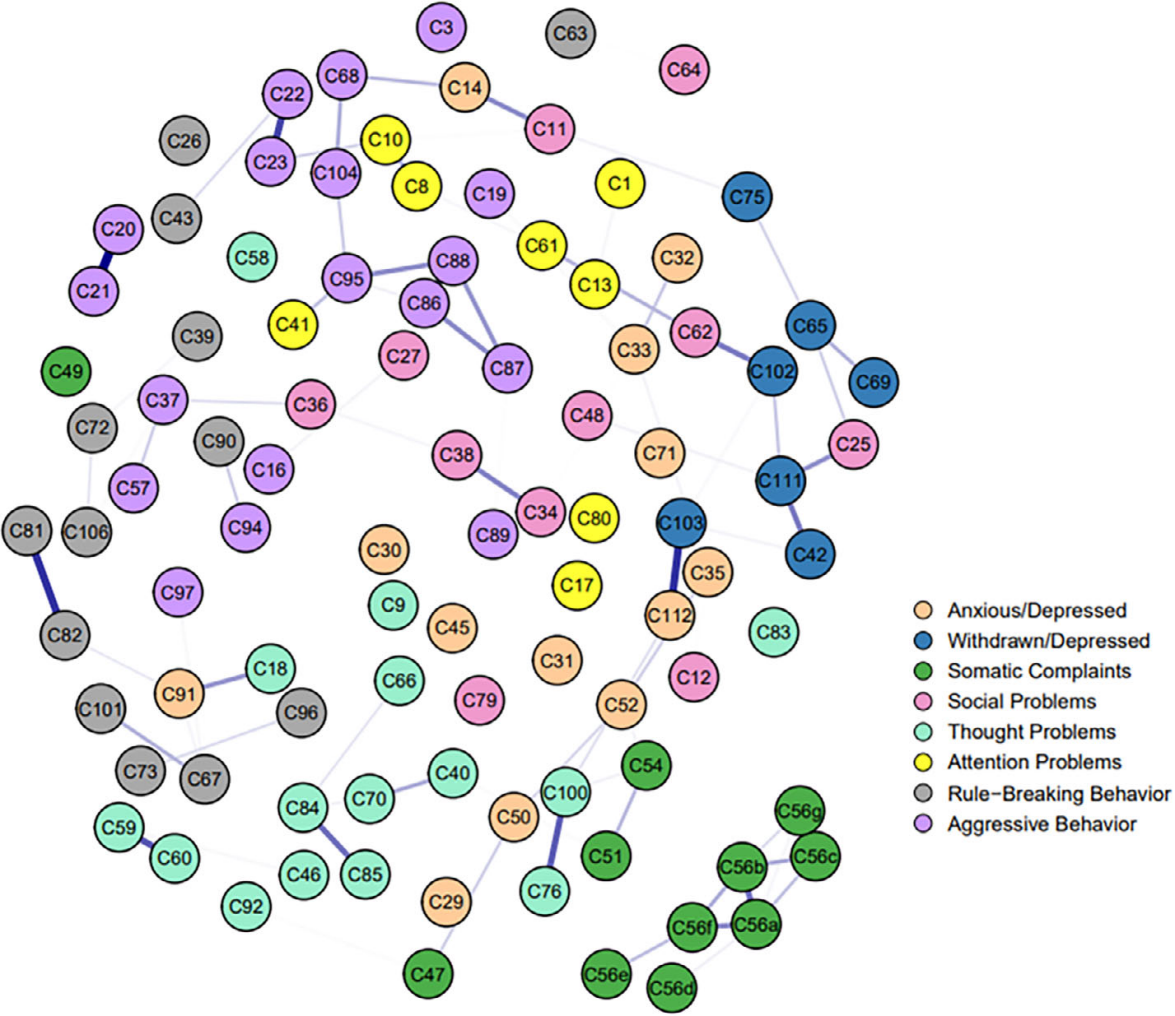
Networks of rural groups with mental disorders.

**FIGURE 4:**
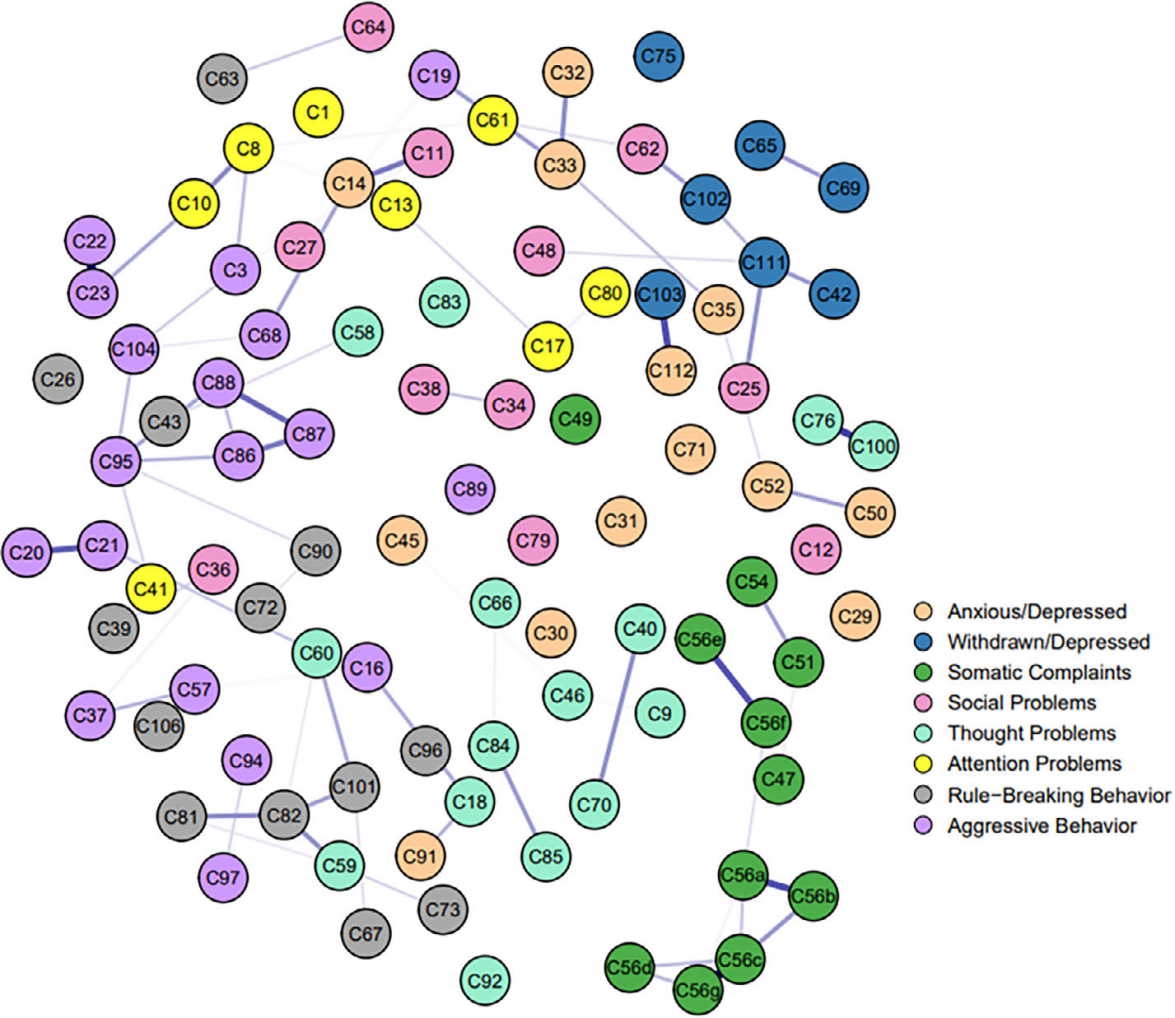
Networks of urban groups with mental disorders.

Based on all the items within the eight factors from CBCL, we separately established two LASSO networks for the rural (Figure 3) and urban groups (Figure 4). Similar to the entire sample networks, these networks were also characterized by a preponderance of weak or non‐significant connections. Examples include the generally weak associations between “Somatic complaints” and “Thought problems” items, as well as many within the “Attention problems” dimension. In both samples, the strongest weighted edge was from C56c to C56g, with partial correlation values in the rural and urban networks of 0.337 and 0.320. Additionally, the edges C103 (feeling down) to C112 (excessive worrying), C76 to C100, and C20 to C21 also exhibited strong partial correlations in both samples, with correlation values (rural/urban) of 0.290/0.250, 0.250/0.263, and 0.337/0.235, indicating good internal consistency. In the rural sample, the node with the strongest strength is C103, with a CS‐coefficient of 0.59. In the urban sample, the node with the strongest strength is C60, with a CS‐coefficient of 0.44 (see Figures S7–S12 for additional information).

In terms of the differences between the two samples, a clear connection was observed between C56e and C56f, C87 (sudden mood changes), and C88 in the urban sample, but this connection was absent in the rural sample. In the rural sample, connections were observed between C34 (feeling deliberately teased by others) and C38 (often teased by others), C42 (prefers solitude) and C111 (lonely, socially isolated), C59 and C60, C62 (awkward movements) and C102 (inactive, slow or lack of energy), C81 and C82, C84 and C85, C86 (stubborn, frowning or easily angered) and C88, while these connections were not observed in the urban sample.

The results of the NCT showed significant differences between the two networks in edge weights (*M* = 0.223, *P* = 0.001), but no significant difference in the global strength of the networks (*S* = 3.245, *P* = 0.358). When compared, 246 edges showed significant differences in edge weights, and nine nodes showed significant differences in central strength between the two networks.

### Network comparisons and interactions by residence, age, and gender

All network visualizations, stability analyses (CS‐coefficients), edge‐weight confidence intervals, and detailed centrality indicators for the reported comparisons are provided in the Supplementary Materials (Figures S12–S76). Network comparisons between rural and urban areas, moderated by age group and gender, revealed several key patterns. When examining age interactions, the global structure and strength of psychological networks differed significantly between children and adolescents in both rural and urban settings (*P* < 0.001 for both; see network plots in Figures S13 and S14 for rural, Figures S15 and S16 for urban, and centrality comparisons in Figures S26 and S27). This age‐related difference in network global structure persisted in the urban mental disorder subsample (*P* < 0.001; Figure S18), but not in the corresponding rural subsample.

Furthermore, adolescent networks significantly differed between rural and urban areas in both global structure (*P* = 0.004) and strength (*P* = 0.002), as evidenced by direct comparisons of their network metrics (see Figures S28 and Figures S30 for structure and strength invariance tests, respectively). After controlling for age, significant rural‐urban differences were observed in the network structure and global strength for boys in both the entire sample (structure: *P* < 0.001; strength: *P* < 0.001) and the mental disorder subsample (structure: *P* = 0.030; strength: *P* < 0.001). The supporting network visualizations and centrality indicator comparisons for boys are provided in Figures S46–S49 and Figures S66–S69. In contrast, no significant gender‐by‐residence interactions were found for girls in either sample (all *P* > 0.05).

## DISCUSSION

Using a large and representative sample of children and adolescents, our network analysis demonstrated significant disparity in the interplay between psychopathological symptoms in rural and urban areas. Notably, the estimated networks for both groups were characteristically sparse, with a majority of potential symptom connections being weak or absent—a pattern extending to notably weak inter‐dimensional connections.^33^ This limited and compartmentalized connectivity highlights the relative independence of many psychological constructs and symptom clusters, which aligns with the conceptual framework of the CBCL and the principle of conditional independence often observed in psychopathological networks.^34^ Within these sparse networks, we employed expected influence as our primary centrality metric to identify pivotal nodes. Symptoms with the highest expected influence values (e.g., C11 in rural and C60 in urban) are interpreted as the most influential risk factors, possessing the greatest potential to activate other symptoms, whereas nodes with the lowest expected influence may function as protective factors.^34^ Key findings can be summarized as follows: First, symptoms such as “anxious/depressed”, “withdrawn/depressed”, “somatic complaints”, and “attention problems” were more evident in the rural subsample, but symptoms of “social Problems” and “rule‐breaking behavior” were more common in the urban subsample. Second, the psychopathological profiles of children and adolescents between these two regions diverged from a network perspective. This distinction was evident not only in pivotal psychological nodes but also in the strength of connections between these critical nodes. It can be inferred that children and adolescents in rural areas were more likely to experience emotional and psychological problems, especially symptoms related to depression; whereas children and adolescents in urban areas were more likely to exhibit behavioral symptoms, particularly those associated with attention‐deficit/hyperactivity disorder (ADHD).

The disparities in psychological problems observed between rural and urban children and adolescents were not solely found in China. For instance, in a population‐based cohort study conducted in Denmark, which included newborns between 1955 and 2006 (*n* = 2 894 640), individuals born in the capital exhibited a higher incidence of psychiatric disorders compared to those born in rural areas.^35^ Research from the UK has also observed rural‐urban differences in mental health, specifically focusing on depression.^36^ Another study found that urban prevalence of psychiatric disorders might be marginally higher, with a specific emphasis on higher incidence of depression in urban compared to rural areas.^37^ However, these studies mainly focused on the different incidence or distribution patterns of mental disorders, yet seldom addressed the differences in psychopathological characteristics. The current study provided evidence for understanding how the differences between rural and urban areas impact the psychopathological characteristics of Chinese children and adolescents.

Adopting the network perspective, differences in psychopathology between rural/urban children and adolescents in China were illustrated. The disparities in psychological problems observed between rural and urban areas might be attributed to several factors. First, children in rural areas may more frequently face socioeconomic disadvantages compared to their urban counterparts.^4^ Limited access to resources, lower family income, and less exposure to diverse educational opportunities could lead to stress and other mental health challenges among rural children.^38^ Mental health resources may be less available in rural areas in China, which poses challenges to health policy makers in the post COVID‐19 era.^16^, ^39^ Second, urban areas in China generally have more advanced and accessible mental health services; children and adolescents in rural regions may lack proper early diagnosis and intervention, leading to exacerbated mental health issues over time.^20^ Third, children and adolescents from either rural or urban areas may shoulder different burdens. The pressure of urban living, coupled with the competitive education system in cities, could lead to increased levels of behavioral issues in urban children, while rural children might face pressures related to family expectations, especially in agricultural settings.^40^ Fourth, Chinese parents might have migrated from rural to urban areas in search of work, leaving their children behind to be cared for by grandparents or other relatives.^41^ This “left‐behind” situation has been suggested to cause feelings of neglect, depression, and other psychological challenges for rural children.^9^ Overall, all the aforementioned factors could potentially lead to differing psychopathological characteristics among children and adolescents in rural and urban areas. Notably, these disparities were particularly pronounced among boys, whose symptom networks differed significantly between rural and urban settings, whereas no such residence‐based difference was observed for girls. This suggests that boys’ mental health may be more sensitive to contextual disparities, a pattern supported by recent studies on gender‐specific environmental sensitivity.^42^, ^43^ This provides a reference basis for us to implement different mental health policies in different regions.

Children and adolescents from rural areas of China predominantly grapple with emotionally related challenges, especially those associated with depression. The family structure and parent‐child relationships in rural areas often involve extended periods of separation due to parents migrating to cities for work, leading to a surge of “left‐behind children” who may experience increased feelings of withdrawal, depression, and somatic complaints.^44^, ^45^ The scarcity of knowledge about mental health, coupled with parents' lack of attention to their children's psychological well‐being, might have intensified these emotional issues.^46^ Most critically, the accessibility of health services may also play a role, with urban regions often boasting superior medical and psychological health resources; the mental health resources for children and adolescents in rural regions were reported to be markedly limited.^47^ This dearth of support means that when they encounter mental health challenges, they often lack the appropriate and timely assistance they desperately need.

Conversely, children and adolescents from urban areas manifested more behavioral problems, particularly those associated with ADHD. As the epicenters of educational resources, urban areas naturally expose their younger population to heightened academic pressures.^9^ The immense focus of urban parents on academic performance, combined with the competitive education system, may lead to enhanced detection and diagnosis of ADHD and related behavioral issues.^48^ The urban environment, with its unique pressures and demands, may serve as a catalyst for inducing behavioral abnormalities in children.

Our findings hold significant value for mental health policy makers. Firstly, our results indicated that the psychopathological characteristics of children and adolescents vary between rural and urban areas. It is crucial to recognize that the psychological profiles of children and adolescents vary distinctly across different regions. For children and adolescents in rural areas, especially those left behind, screening for emotional issues is imperative, with a particular focus on depressive disorders. Given the scarcity of mental health resources in rural locales,^20^ telemedicine services for mental health care are advisable.^49^ For children and adolescents in urban areas, relevant authorities should prioritize behavioral issues, particularly early screening and intervention for ADHD. Our finding that boys’ psychopathological networks show significant rural‐urban differences further underscores the need to consider gender in tailoring interventions. For instance, support for boys in rural settings might need to address unique contextual stressors.^50^ On the one hand, the scientific understanding of ADHD among parents and teachers should be enhanced to facilitate timely intervention.^51^ On the other hand, it is necessary to establish integrated ADHD prevention and intervention systems, encompassing schools, families, medical institutions, and communities.^52^

Overall, our study revealed notable psychopathological differences between children and adolescents in rural and urban areas, underscoring the need for tailored mental health policies. The highlighted regional disparity calls for region‐specific interventions to address unique challenges. Moreover, the imbalance in mental health resource distribution highlights the need for amplified support in rural areas.^53^ These findings call for societal action to prioritize the mental well‐being of children and adolescents, endorsing timely interventions crucial for their holistic development and overall well‐being. Through informed resource allocation based on observed psychopathological patterns, a more effective and inclusive mental health framework can be envisioned across diverse regional landscapes.

Several limitations were noted. First, our evaluation of the psychological well‐being of children and adolescents in both rural and urban areas relied solely on the CBCL. This singular approach might have left out other pertinent dimensions of mental health. Future studies may benefit from additional methods such as neuropsychological testing, electroencephalography, and imaging techniques to explore the impact of rural and urban environments on the brain function of children and adolescents.^54^ Second, as a cross‐sectional study, our analysis is limited to describing symptom associations at one point in time. It cannot establish whether the observed network structure remains stable over time or can be altered through intervention.^55^ Lastly, the sample for this research was drawn exclusively from one province in the western part of China. This geographical specificity could limit the generalizability of our findings, as they might not fully represent the psychological profiles of children and adolescents across the entire country. China's uneven economic development, coupled with the imbalanced distribution of mental health resources,^16^, ^47^ may lead to disparities in mental health between rural and urban areas. Going forward, it is essential to conduct further investigations in regions with varying degrees of development to better understand these disparities.

To conclude, our work applied network analysis and a large representative Chinese sample to investigate urban‐rural disparity in mental health. Children and adolescents in rural areas were prone to depressive symptoms, whilst those in urban areas were prone to behavioral problems and ADHD‐related symptoms. Moreover, the interrelationships of symptoms differed between the urban and the rural subsamples. Our findings may benefit mental health policy makers in devising better healthcare services to young people in China.

## CONFLICT OF INTEREST

The authors declare no conflict of interest.

## Supporting information



Supporting Information
