# Supplementary material for: Network analysis of psychological problems in school‐attending students aged 6–16 years in China: A comparison between rural and urban areas
Source: Pediatr Investig. 2026 Apr 14;10(2):135–46. doi: 10.1002/ped4.70056 (PMC13109867; doi:10.1002/ped4.70056)
Supplement: Supplementary file 1 — Supporting Information [file PED4-10-135-s001.pdf]

Supplementary Material for

Network analysis of psychological problems in school-attending students aged 6–16 years in China: A comparison between rural and urban areas

Lianjingyi Liang, Wanling Zhang, Ying Li, Yanyu Wang, Raymond C K Chan

Table S1. Abbreviation of 8-factor dimension

Full title	Abbreviation
Anxious/Depressed	AD
Withdrawn/Depressed	WD
Somatic Complaints	SC
Social Problems	SP
Thought Problems	TP
Attention Problems	AP
Rule-Breaking Behavior	RB
Aggressive Behavior	AB

Table S2. The content of each CBCL item

Item	Content	Item	Content	Item	Content
CBCL1	Acts young	CBCL41	Impulsive	CBCL70	Sees things
CBCL3	Argues	CBCL42	Prefers solitude	CBCL71	Self-conscious
CBCL8	Can't concentrate	CBCL43	Lies, cheats	CBCL72	Sets fires
CBCL9	Can't get mind off thoughts	CBCL45	Nervous	CBCL73	Sex problems
CBCL10	Can't sit still	CBCL46	Twitching	CBCL75	Shy
CBCL11	Clinging to adults or excessively dependent	CBCL47	Nightmares	CBCL76	Less sleep
CBCL12	Lonely	CBCL48	Unliked	CBCL79	Speech problems
CBCL13	Confused	CBCL49	Constipated	CBCL80	Stares blankly
CBCL14	Cries a lot	CBCL50	Anxious	CBCL81	Stealing at home
CBCL16	Mean	CBCL51	Dizzy	CBCL82	Stealing outside
CBCL17	Daydreams	CBCL52	Feels guilty	CBCL83	Stores things
CBCL18	Harms self	CBCL54	Overtired	CBCL84	Strange behavior
CBCL19	Demands attention	CBCL56a	Aches	CBCL85	Strange ideas
CBCL20	Damaging one's own things	CBCL56b	Headaches	CBCL86	Stubborn, frowning or easily angered
CBCL21	Damaging things at home or of others	CBCL56c	feeling sick	CBCL87	Sudden mood changes
CBCL22	Disobedience at home	CBCL56d	Eye problems	CBCL88	Frequently angry
CBCL23	Disobedience at school	CBCL56e	Skin rash or other skin issues	CBCL89	Suspicious
CBCL25	Doesn't get along	CBCL56f	Abdominal pain or cramps	CBCL90	Swearing
CBCL26	Lacks guilt	CBCL56g	Vomiting	CBCL91	Talks about suicide
CBCL27	Jealous	CBCL57	Attacks people	CBCL92	Sleep talks/walks
CBCL29	Fears	CBCL58	Picks skin	CBCL94	Teases
CBCL30	Fears school	CBCL59	Public genital play	CBCL95	Temper tantrums or irritability
CBCL31	Fears doing bad	CBCL60	Excessive genital play	CBCL96	Thinks of sex too much
CBCL32	Must be perfect	CBCL61	Poor schoolwork	CBCL97	Threats
CBCL33	Feels unloved	CBCL62	Awkward movements	CBCL100	Poor sleep quality
CBCL34	Feeling deliberately teased by others	CBCL63	Prefers to play with older children	CBCL10l	Truant
CBCL35	Feels worthless	CBCL64	Prefers to play with younger children	CBCL102	Inactive, slow or lack of energy
CBCL36	Accident-prone	CBCL65	Won't talk	CBCL103	Feeling down
CBCL37	Fights	CBCL66	Repeats acts	CBCL104	Loud
CBCL38	Often teased by others	CBCL67	Runs away	CBCL106	Vandalism
CBCL39	Bad friends	CBCL68	Screams	CBCL111	Lonely, socially isolated
CBCL40	Hears things	CBCL69	Secretive	CBCL112	Excessive worrying

Table S3. Demographic characteristics of participants (Entire sample)

**Characteristic**	**Urban (*n* = 9 566)**	**Rural (*n* = 10 145)**	**Statistic**	***P*-value**
Age, years	11.4 ± 3.2	12.0 ± 2.8	$t = -12.442$	<0.001
Age group			$\chi^2 = 20.601$	<0.001
6–11 years	4485 (46.9)	4430 (43.7)		
12–16 years	5081 (53.1)	5715 (56.3)		
Gender			$\chi^2 = 13.520$	<0.001
Boy	4952 (51.8)	4986 (49.1)		
Girl	4614 (48.2)	5159 (50.9)		
Co-residence with both parents	4647 (48.6)	4614 (45.5)	$\chi^2 = 4.317$	0.038
Only Child	5666 (59.2)	6022 (59.4)	$\chi^2 = 0.034$	0.854
Han/non-Han	9467 (99.0)	10 039 (99.0)	$\chi^2 = 1.444$	0.229

Data are presented as mean ± standard deviation or *n* (%)

Table S4. Scores of CBCL total score in different subgroups (Entire sample)

Sample	*n*	P_{25}	P_{50}	P_{60}	P_{70}	P_{75}	P_{80}	P_{85}	P_{90}	P_{95}	P_{98}	95% *CI*	Skewness	Kurtosis
Urban	9566	13	25	30	36	39	43	48	54	65	78	(27.777, 28.601)	1.818	8.499
Rural	10 145	14	25	30	36	39	43	48	55	66	80	(27.926, 28.706)	1.336	3.481
Without mental disorder	16 708	12	21	26	31	33	36	40	44	51	61	(23.226, 23.693)	1.602	3.065
With mental disorder	3003	41	52	58	63	66	70	74	80	92	120	(54.098, 55.764)	1.759	7.890
Entire	19 711	13	25	30	36	39	43	48	55	66	79	(27.971, 28.538)	1.579	6.040

Figure	Title
Figure S1	95% CI of rural network of entire sample
Figure S2	95% CI of urban network of entire sample
Figure S3	Stability of rural network of entire sample
Figure S4	Stability of urban network of entire sample
Figure S5	Centrality indicators of rural network of entire sample
Figure S6	Centrality indicators of urban network of entire sample
Figure S7	95% CI of rural network of sample with mental disorder
Figure S8	95% CI of urban network of sample with mental disorder
Figure S9	Stability of rural network of sample with mental disorder
Figure S10	Stability of urban network of sample with mental disorder
Figure S11	Centrality indicators of rural network of sample with mental disorder
Figure S12	Centrality indicators of urban network of sample with mental disorder
Figure S13	Networks of rural children groups of the entire sample
Figure S14	Networks of urban children groups of the entire sample
Figure S15	Networks of rural adolescent groups of the entire sample
Figure S16	Networks of urban adolescent groups of the entire sample
Figure S17	Networks of rural children groups of the mental disorder
Figure S18	Networks of urban children groups of the mental disorder
Figure S19	Networks of rural adolescent groups of the mental disorder
Figure S20	Networks of urban adolescent groups of the mental disorder
Figure S21	95% CI of rural children network of the entire sample
Figure S22	95% CI of urban children network of the entire sample
Figure S23	95% CI of rural adolescent network of the entire sample
Figure S24	95% CI of urban adolescent network of the entire sample
Figure S25	95% CI of rural children network of the mental disorder
Figure S26	95% CI of urban children network of the mental disorder
Figure S27	95% CI of rural adolescent network of the mental disorder
Figure S28	95% CI of urban adolescent network of the mental disorder
Figure S29	Stability of rural children network of entire sample
Figure S30	Stability of urban children of entire sample
Figure S31	Stability of rural adolescent network of entire sample
Figure S32	Stability of urban adolescent of entire sample
Figure S33	Stability of rural children network of mental disorder
Figure S34	Stability of urban children of mental disorder
Figure S35	Stability of rural adolescent network of mental disorder
Figure S36	Stability of urban adolescent of mental disorder
Figure S37	Centrality indicators of rural children and adolescents of entire sample
Figure S38	Centrality indicators of urban children and adolescents of entire sample
Figure S39	Centrality indicators of rural children and adolescents of mental disorder
Figure S40	Centrality indicators of urban children and adolescents of mental disorder
Figure S41	Centrality indicators of rural children and urban children of entire sample
Figure S42	Centrality indicators of rural adolescent and urban adolescent of entire sample
Figure S43	Centrality indicators of rural children and urban children of mental disorder

Figure S44	Centrality indicators of rural adolescent and urban adolescent of mental disorder
Figure S45	Networks of rural boy groups of the entire sample
Figure S46	Networks of urban boy groups of the entire sample
Figure S47	Networks of rural girl groups of the entire sample
Figure S48	Networks of urban girl groups of the entire sample
Figure S49	Networks of rural boy groups of the mental disorder
Figure S50	Networks of urban boy groups of the mental disorder
Figure S51	Networks of rural girl groups of the mental disorder
Figure S52	Networks of urban girl groups of the mental disorder
Figure S53	95% CI of rural boy network of the entire sample
Figure S54	95% CI of urban boy network of the entire sample
Figure S55	95% CI of rural girl network of the entire sample
Figure S56	95% CI of urban girl network of the entire sample
Figure S57	95% CI of rural boy network of the mental disorder
Figure S58	95% CI of urban boy network of the mental disorder
Figure S59	95% CI of rural girl network of the mental disorder
Figure S60	95% CI of urban girl network of the mental disorder
Figure S61	Stability of rural boy network of entire sample
Figure S62	Stability of urban boy of entire sample
Figure S63	Stability of rural girl network of entire sample
Figure S64	Stability of urban girl of entire sample
Figure S65	Stability of rural boy network of mental disorder
Figure S66	Stability of urban boy of mental disorder
Figure S67	Stability of rural girl network of mental disorder
Figure S68	Stability of urban girl of mental disorder
Figure S69	Centrality indicators of rural boy and girls of entire sample
Figure S70	Centrality indicators of urban boy and girls of entire sample
Figure S71	Centrality indicators of rural boy and girls of mental disorder
Figure S72	Centrality indicators of urban boy and girls of mental disorder
Figure S73	Centrality indicators of rural boy and urban boy of entire sample
Figure S74	Centrality indicators of rural girl and urban girl of entire sample
Figure S75	Centrality indicators of rural boy and urban boy of mental disorder
Figure S76	Centrality indicators of rural girl and urban girl of mental disorder

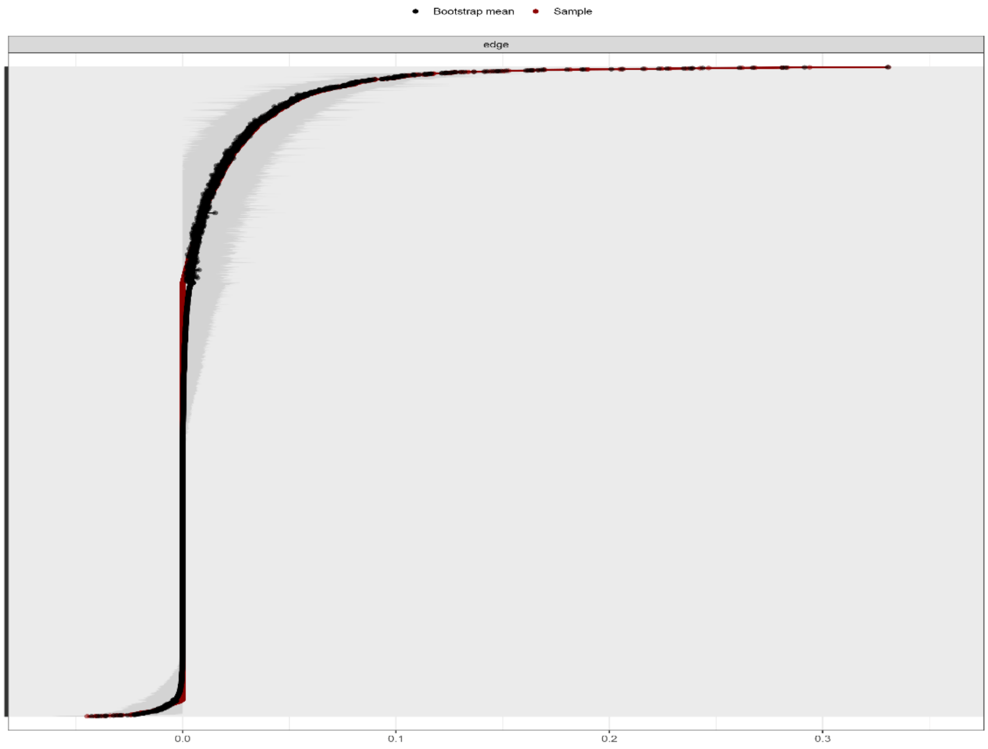

Figure S1 95% CI of rural network of entire sample

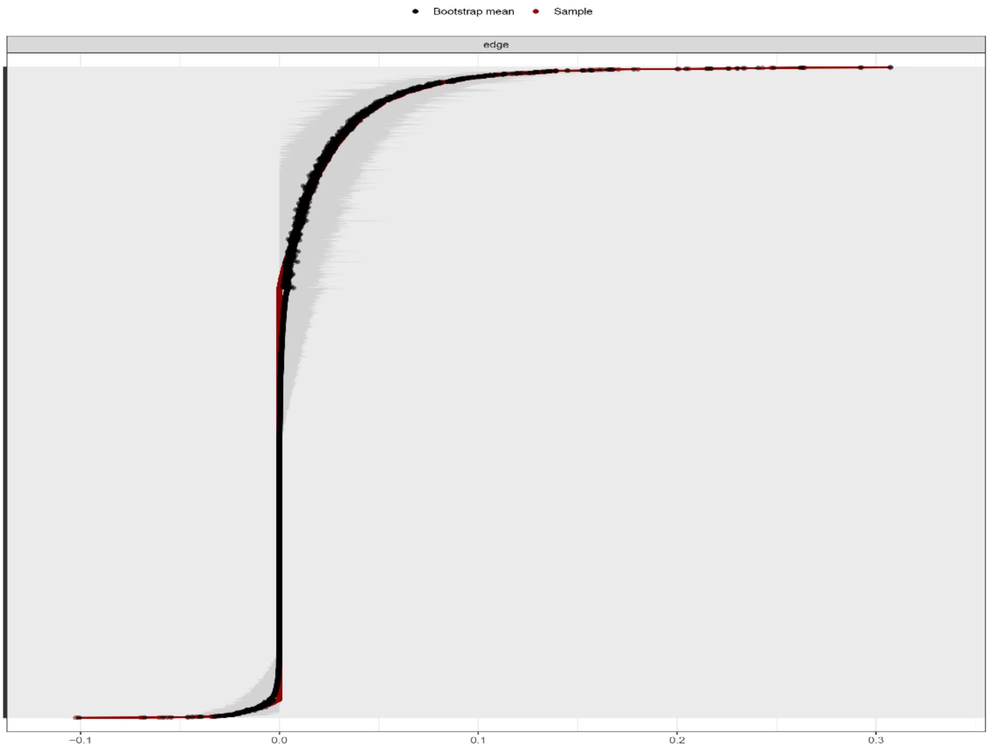

Figure S2 95% CI of urban network of entire sample

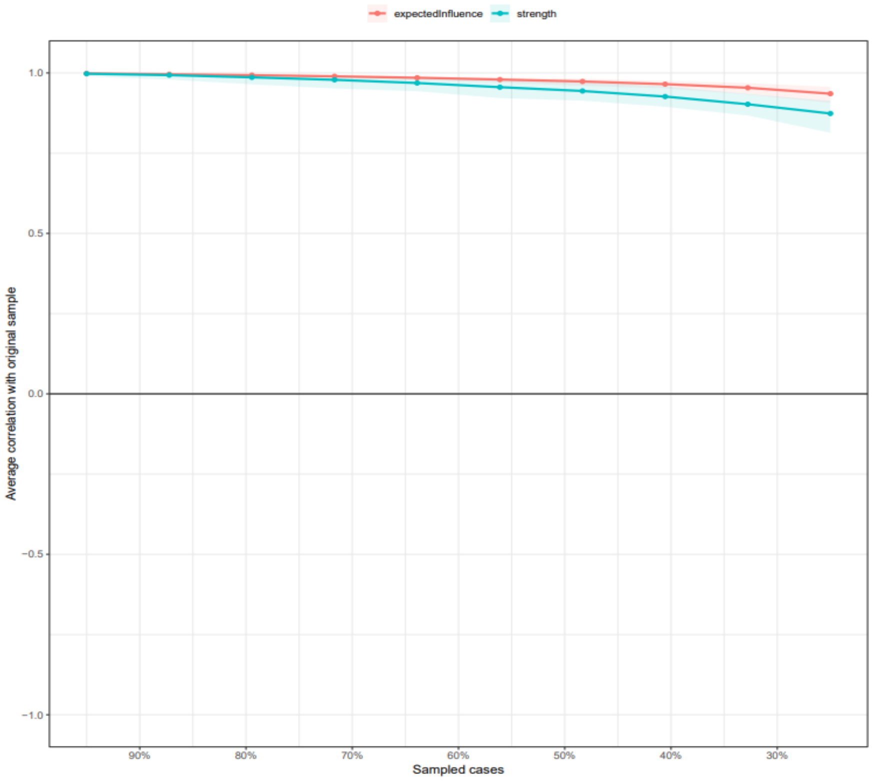

Figure S3 Stability of rural network of entire sample

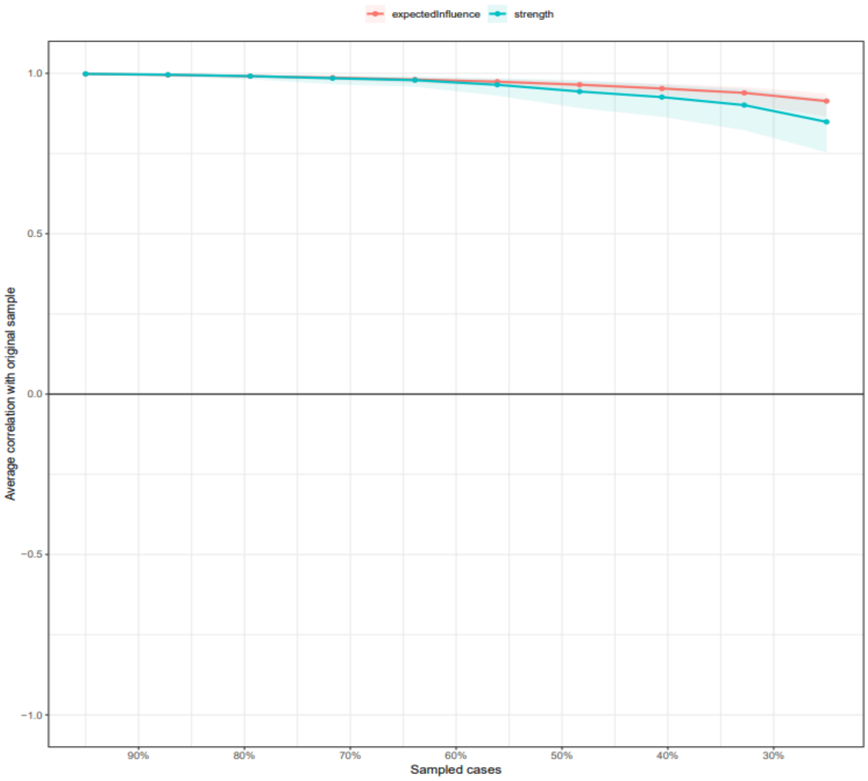

Figure S4 Stability of urban network of entire sample

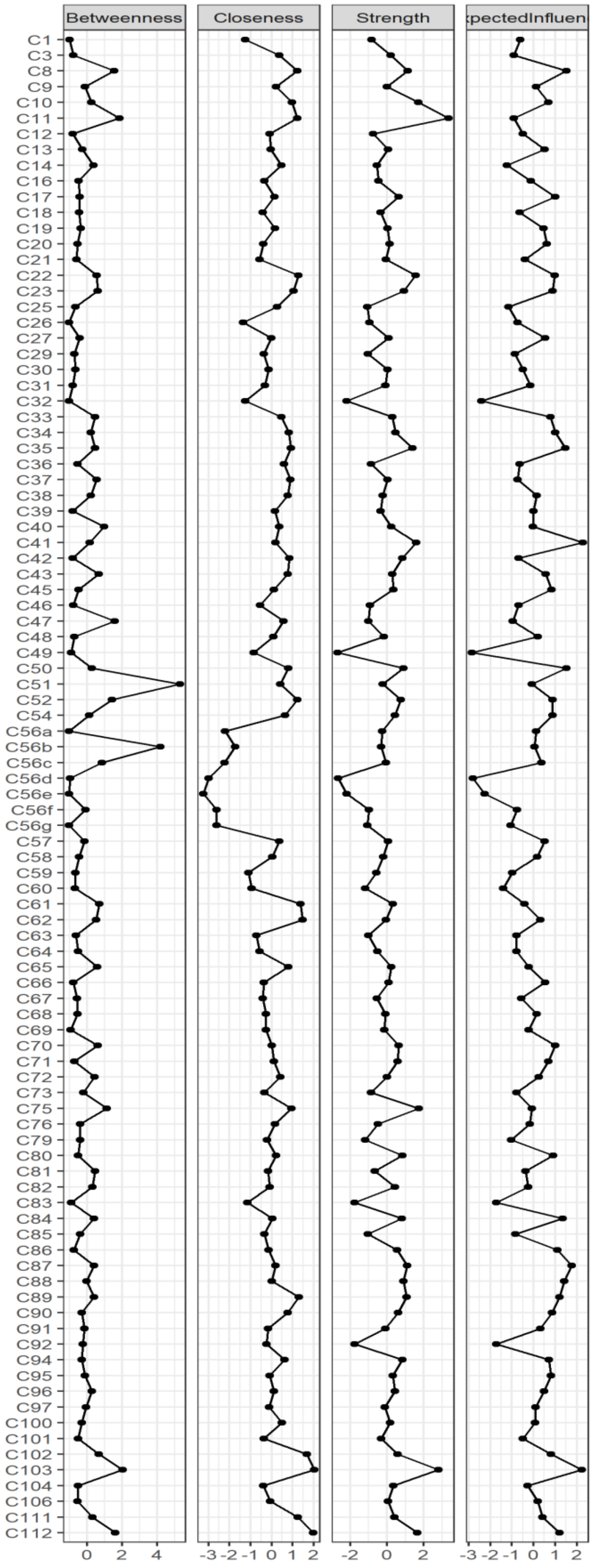

Figure S5 Centrality indicators of rural network of entire sample

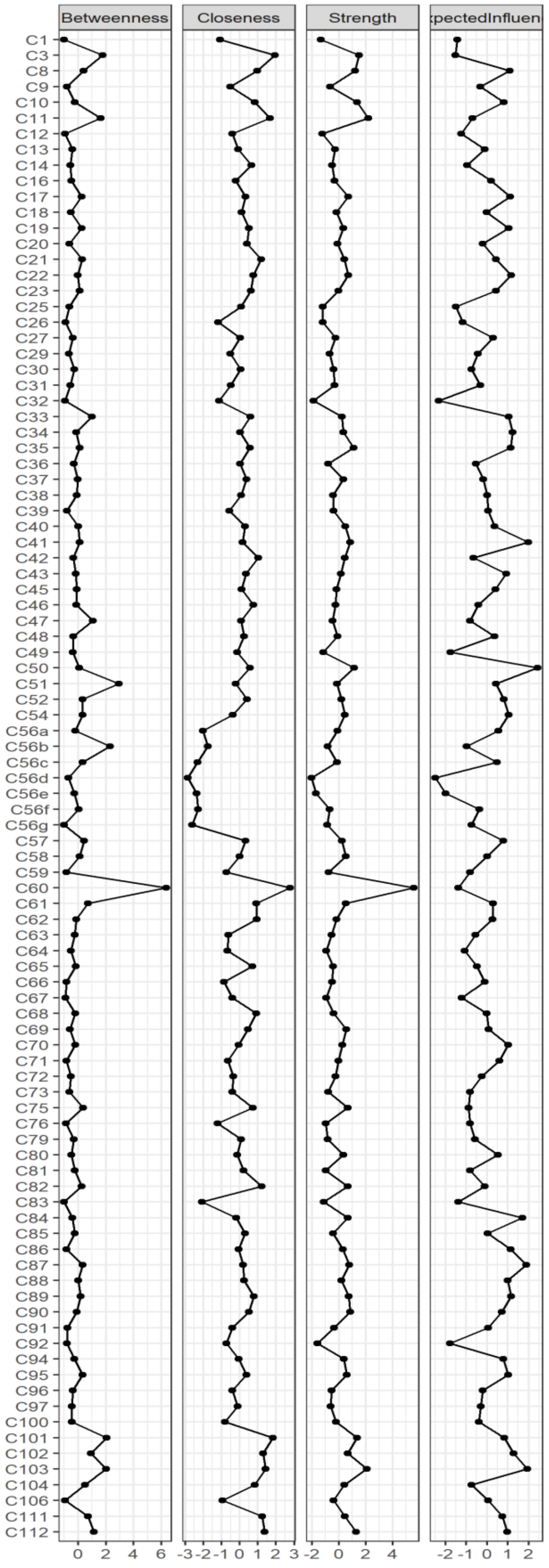

Figure S6 Centrality indicators of urban network of entire sample

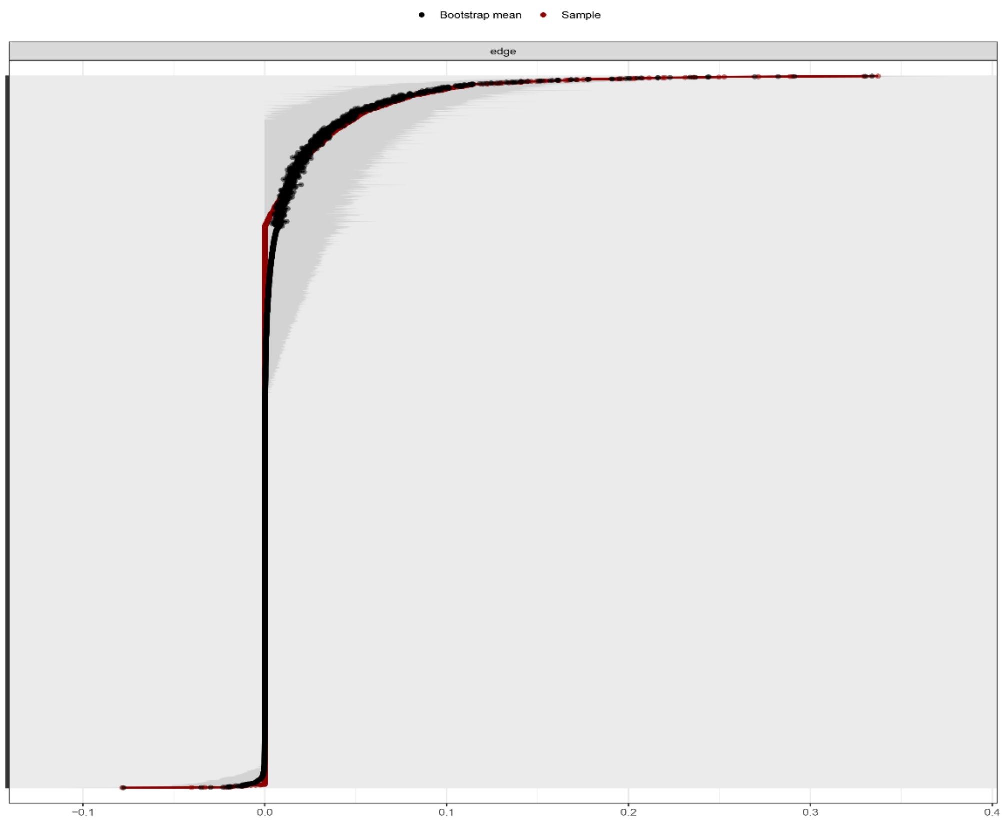

Figure S7   95% CI of rural network of sample with mental disorder

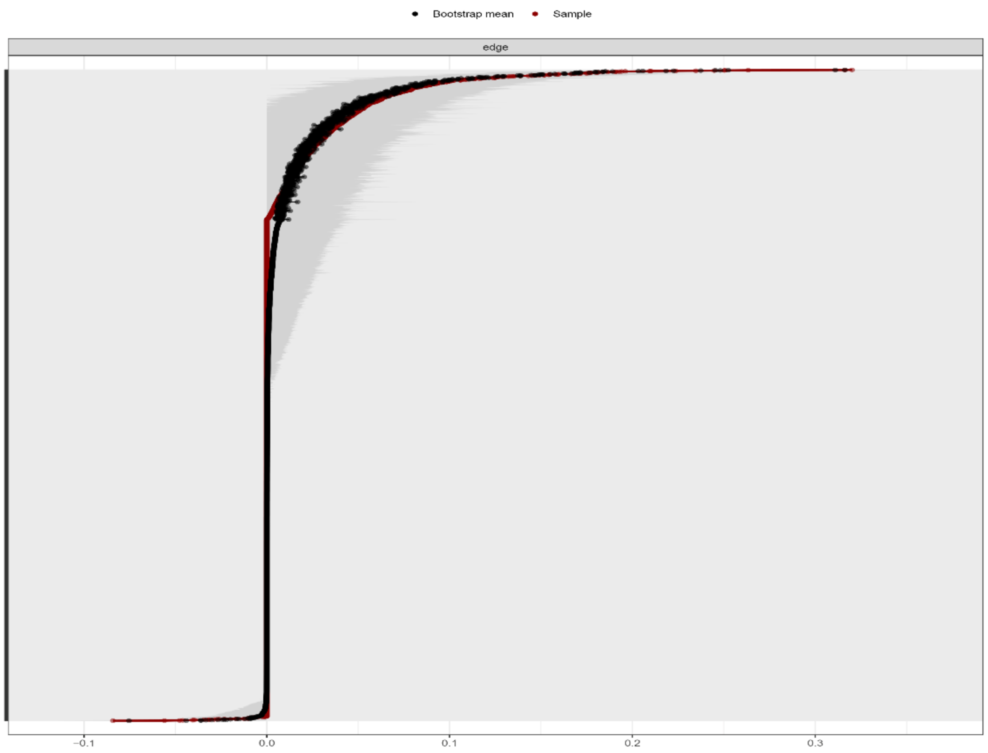

Figure S8   95% CI of urban network of sample with mental disorder

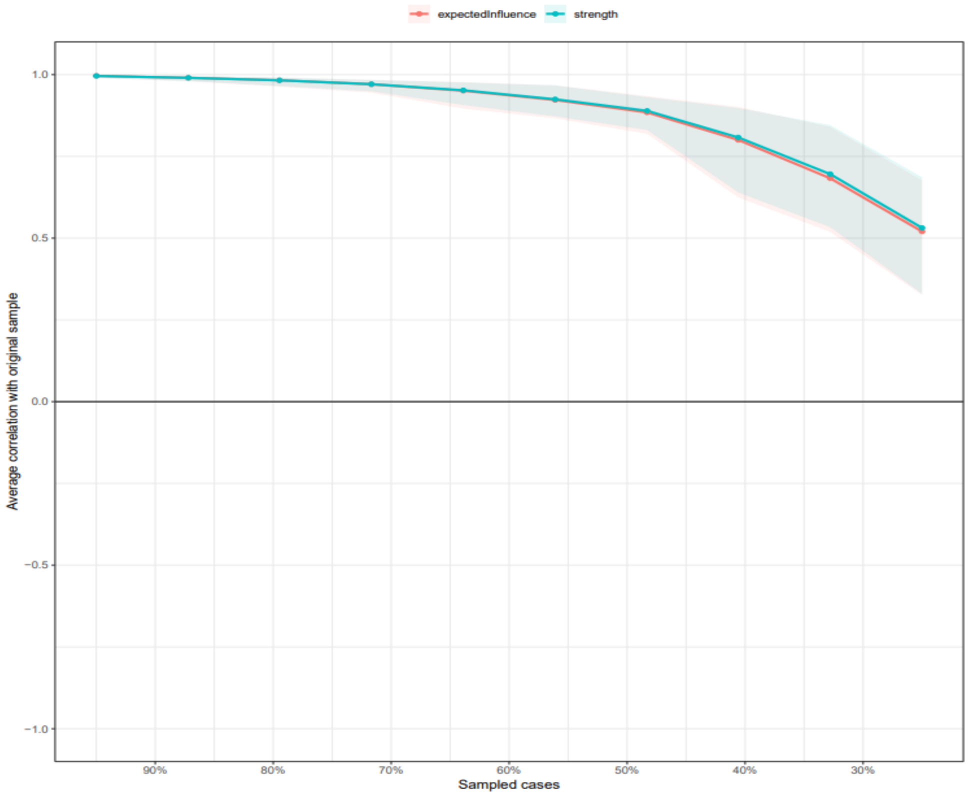

Figure S9 Stability of rural network of sample with mental disorder

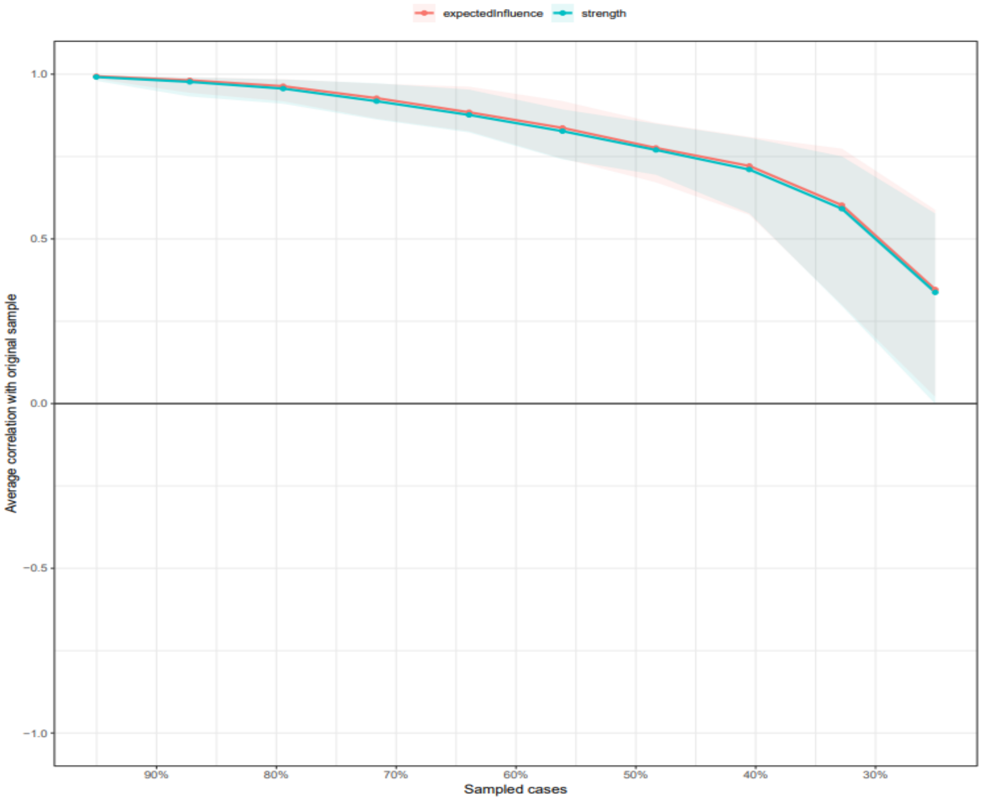

Figure S10 Stability of urban network of sample with mental disorder

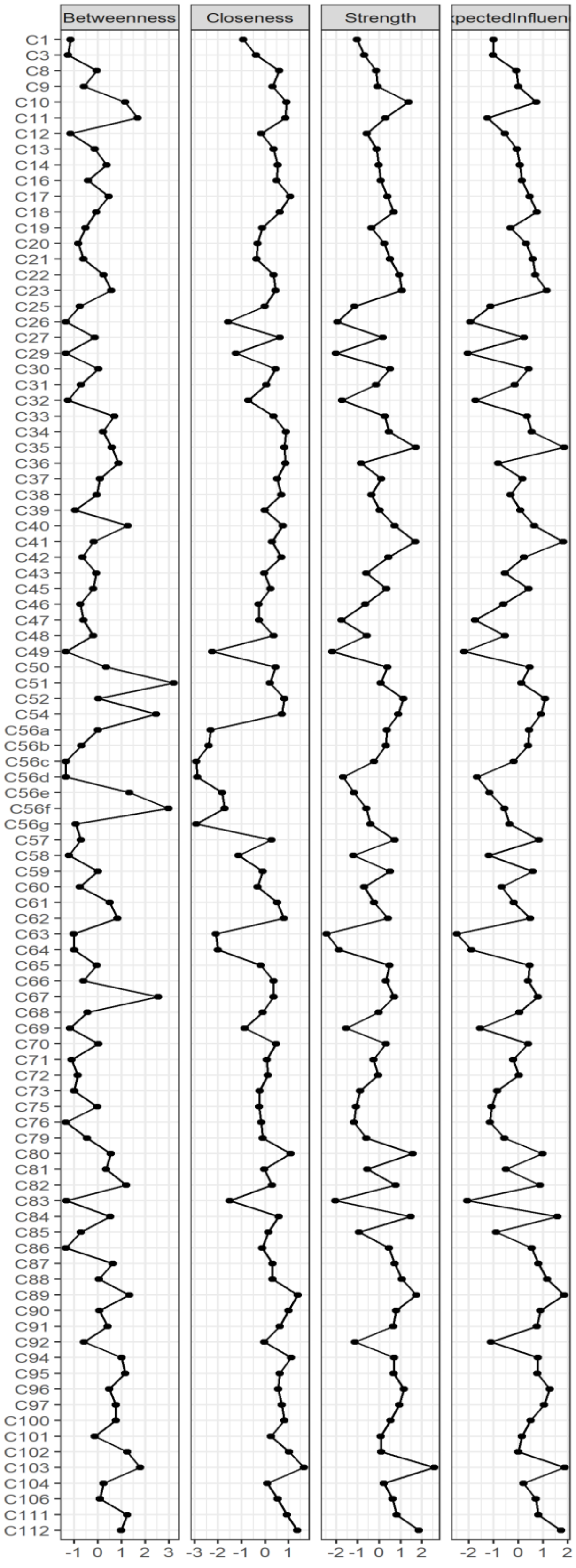

Figure S11 Centrality indicators of rural network of sample with mental disorder

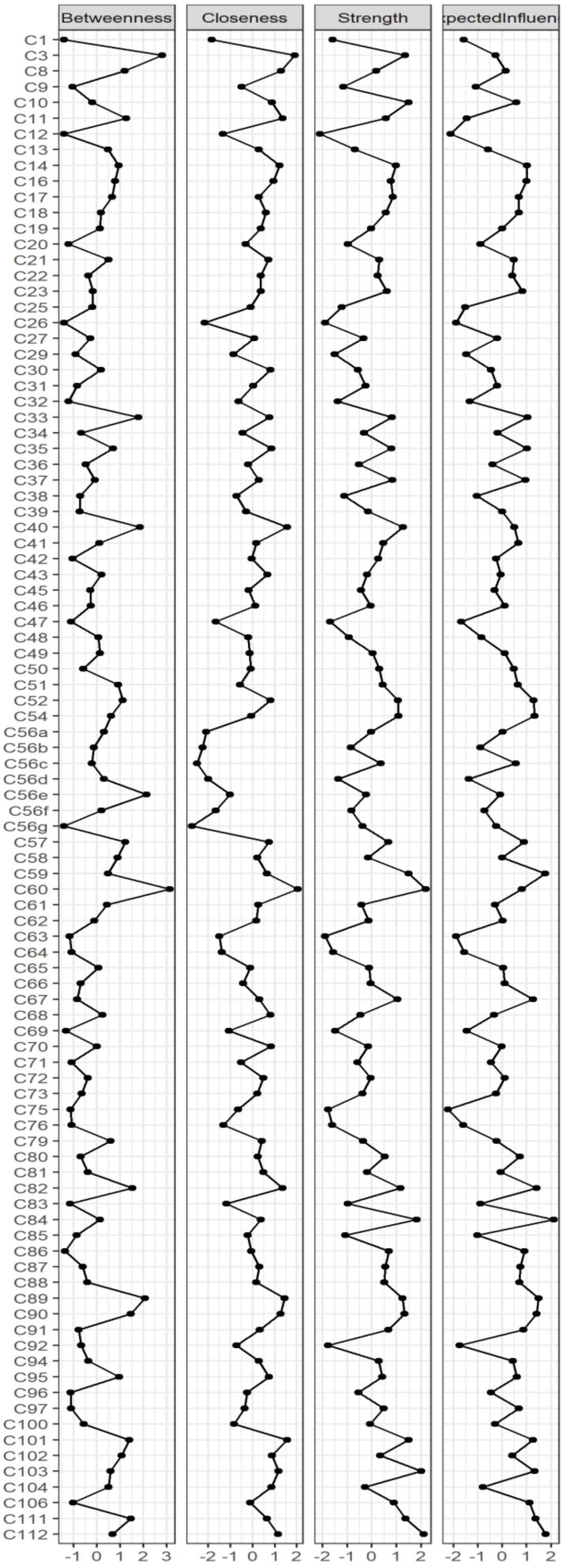

Figure S12 Centrality indicators of urban network of sample with mental disorder

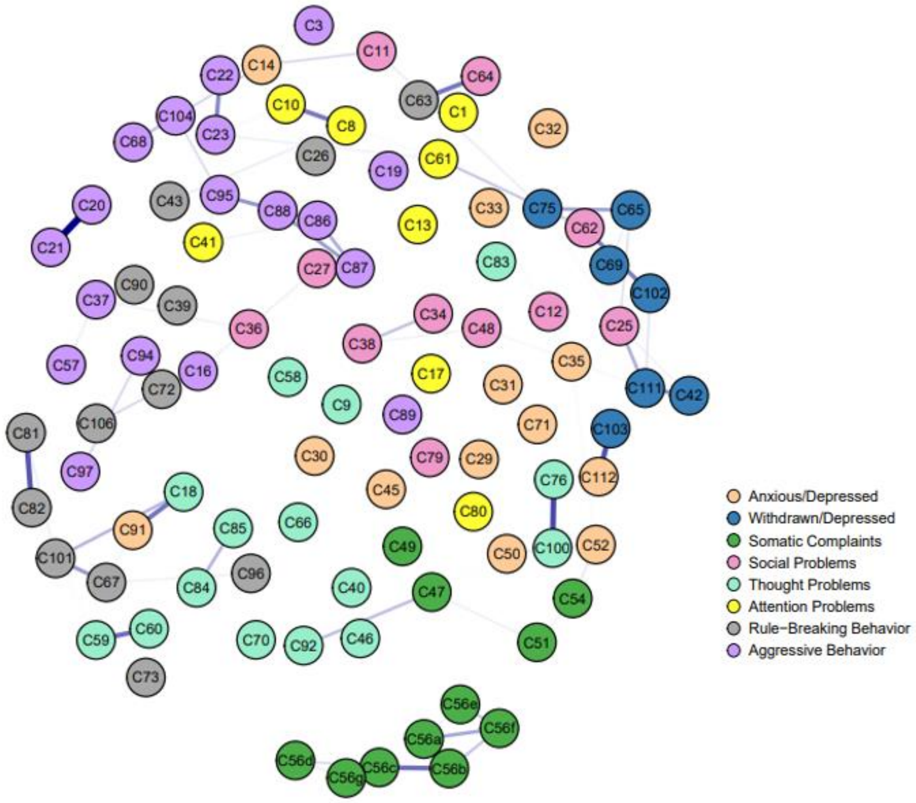

Figure S13 Networks of rural children groups of the entire sample

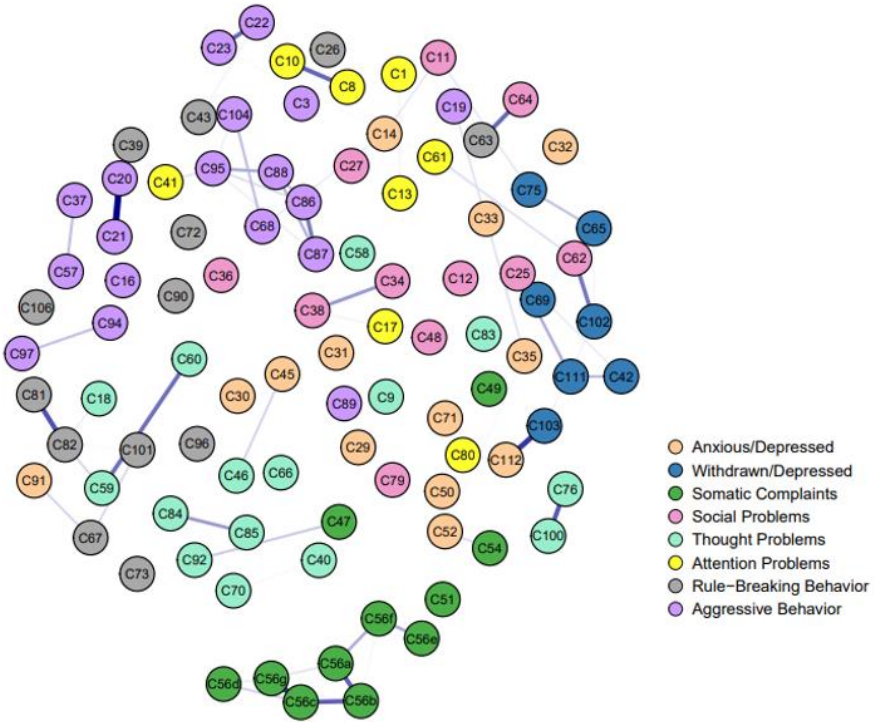

Figure S14 Networks of urban children groups of the entire sample

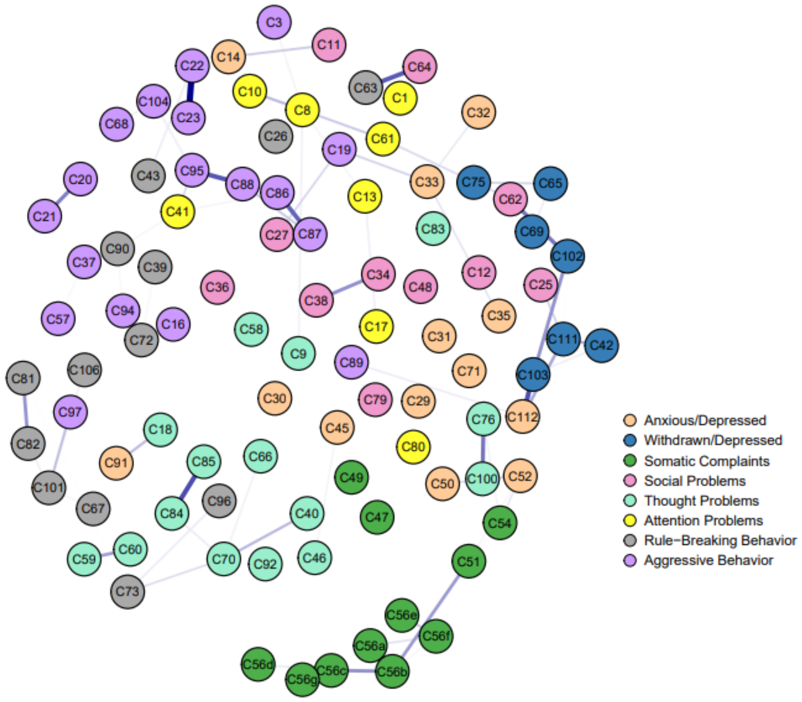

Figure S15 Networks of rural adolescent groups of the entire sample

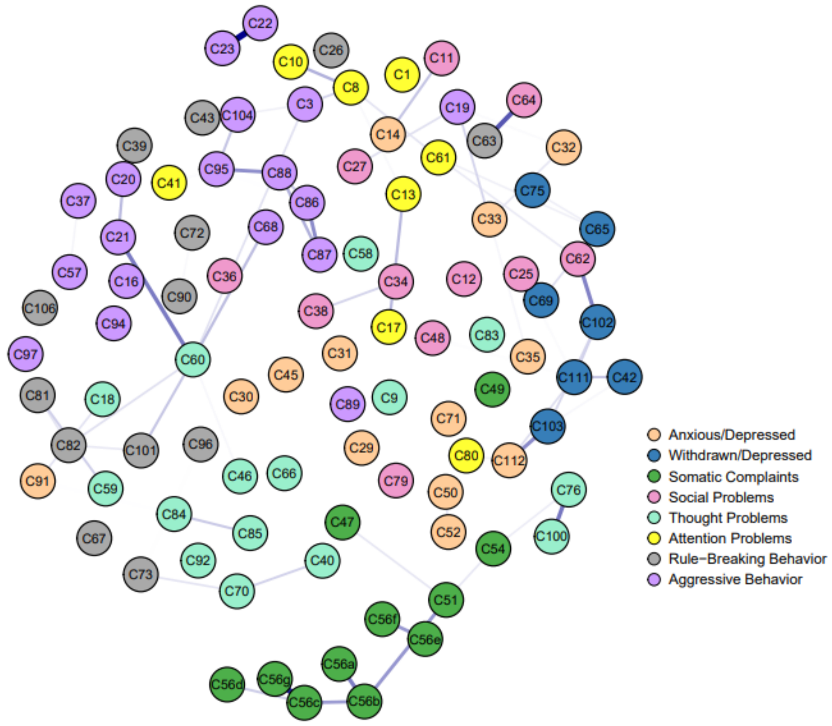

Figure S16 Networks of urban adolescent groups of the entire sample

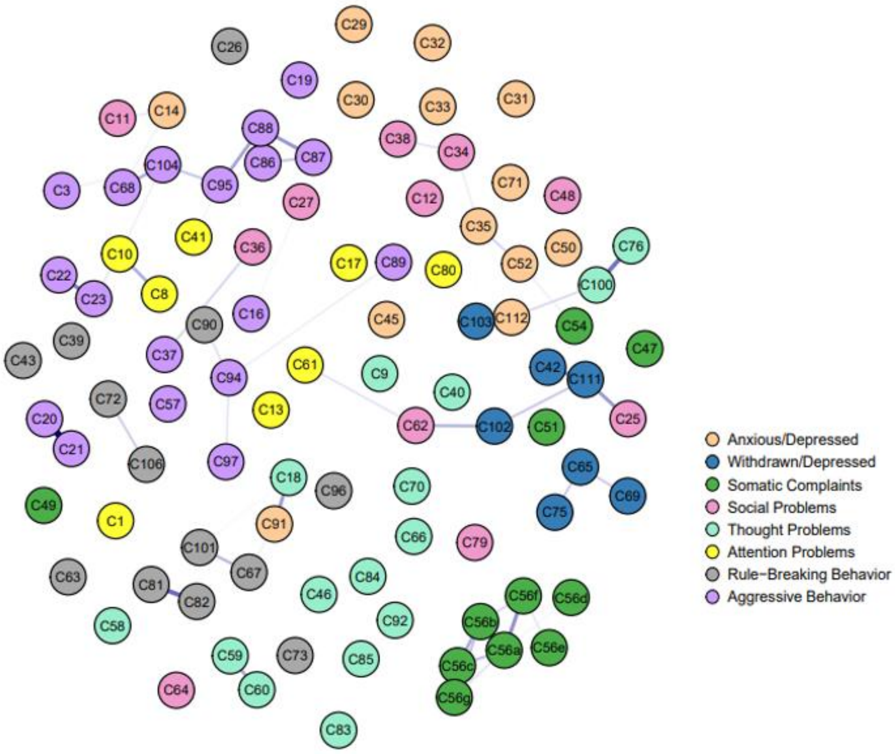

Figure S17 Networks of rural children groups of the mental disorder

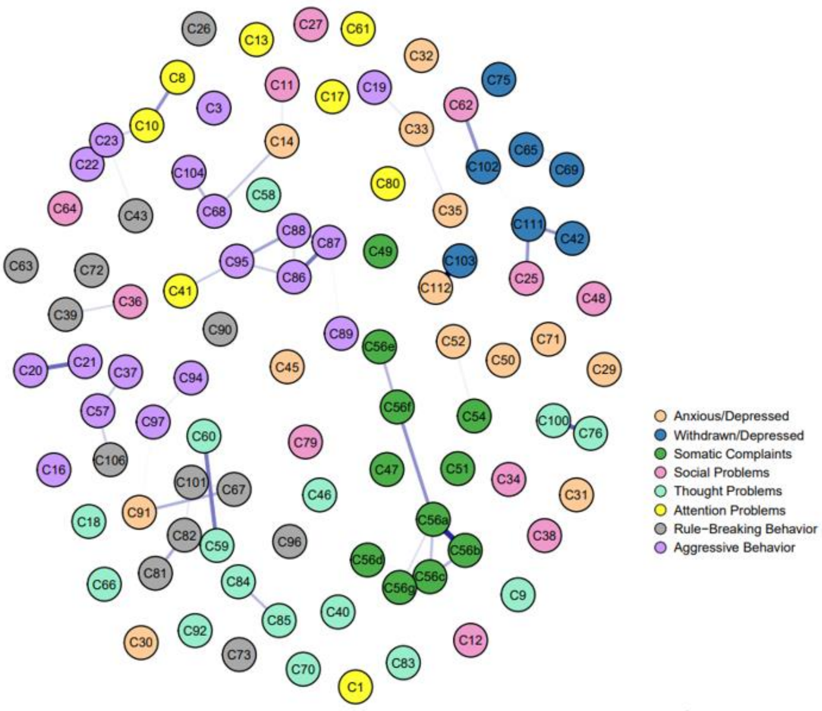

Figure S18 Networks of urban children groups of the mental disorder

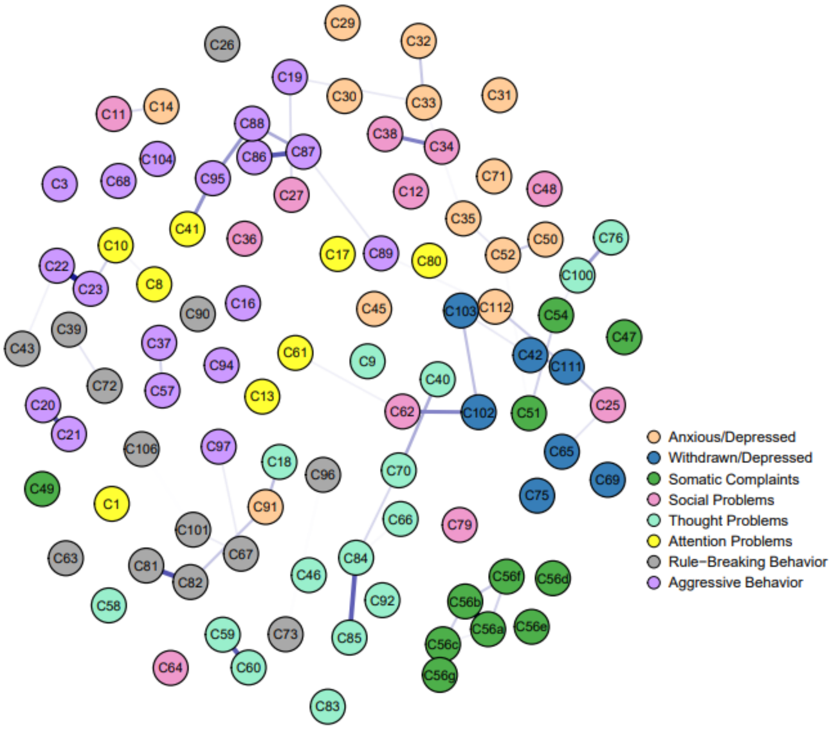

Figure S19 Networks of rural adolescent groups of the mental disorder

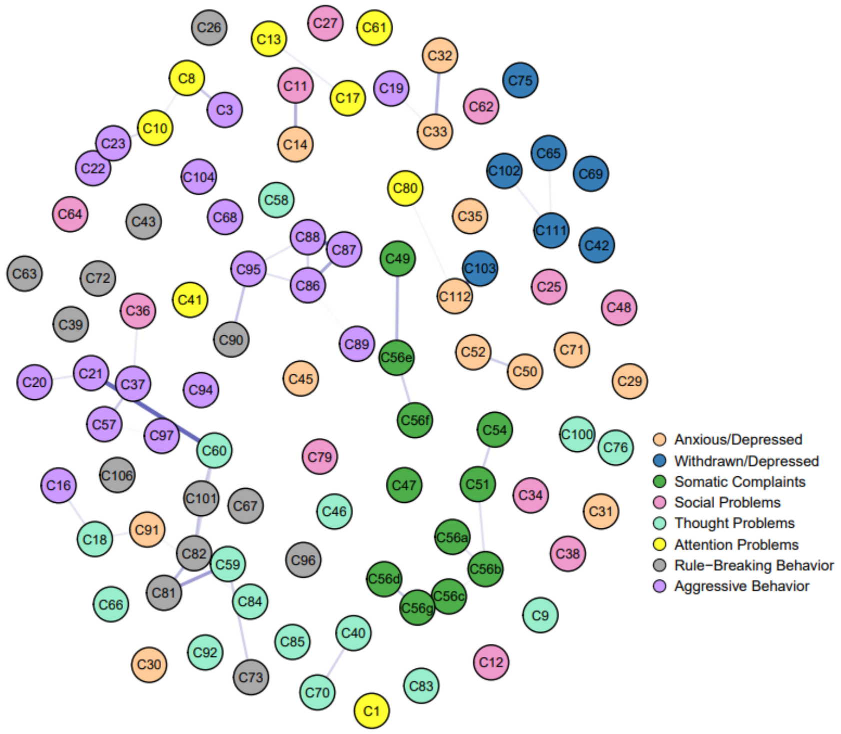

Figure S20 Networks of urban adolescent groups of the mental disorder

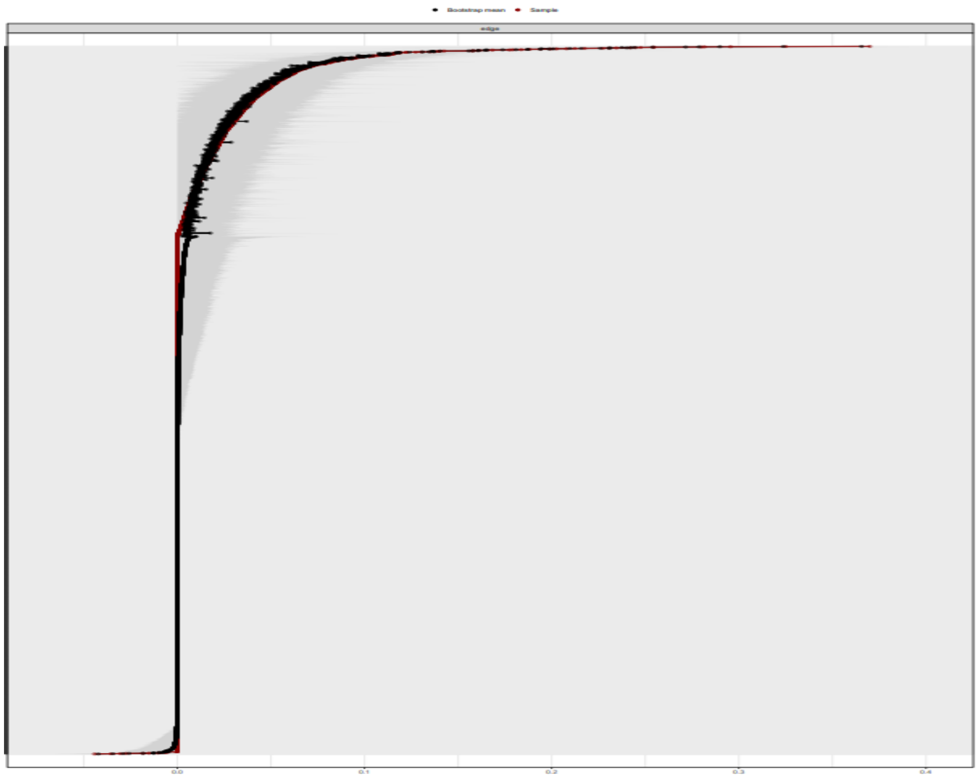

Figure S21　95% CI of rural children network of the entire sample

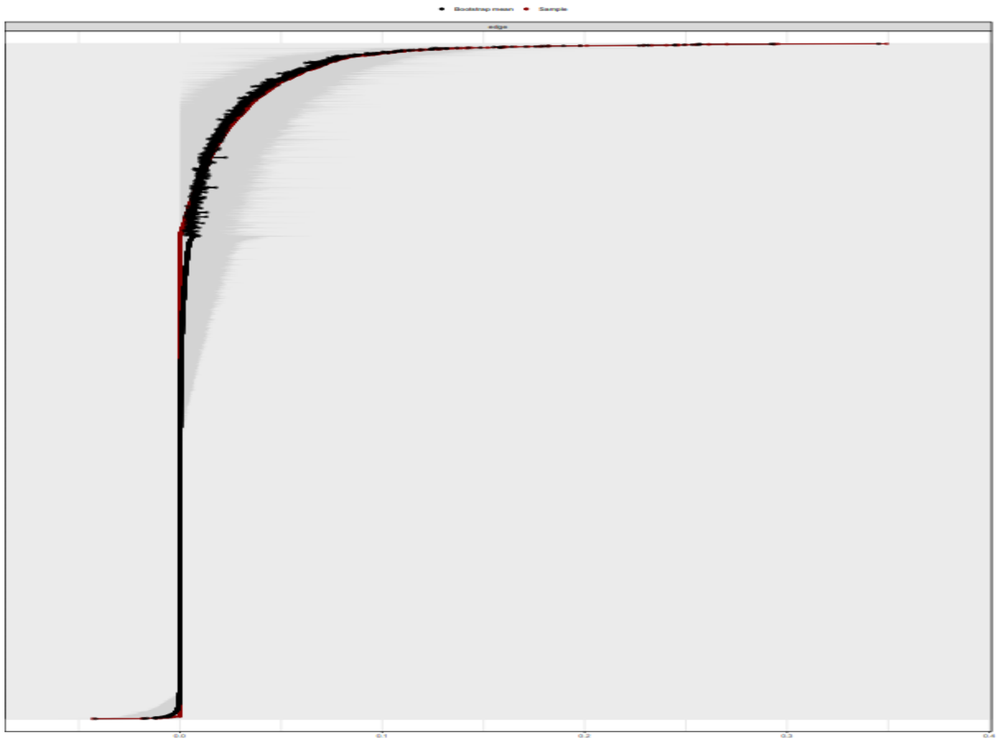

Figure S22　95% CI of urban children network of the entire sample

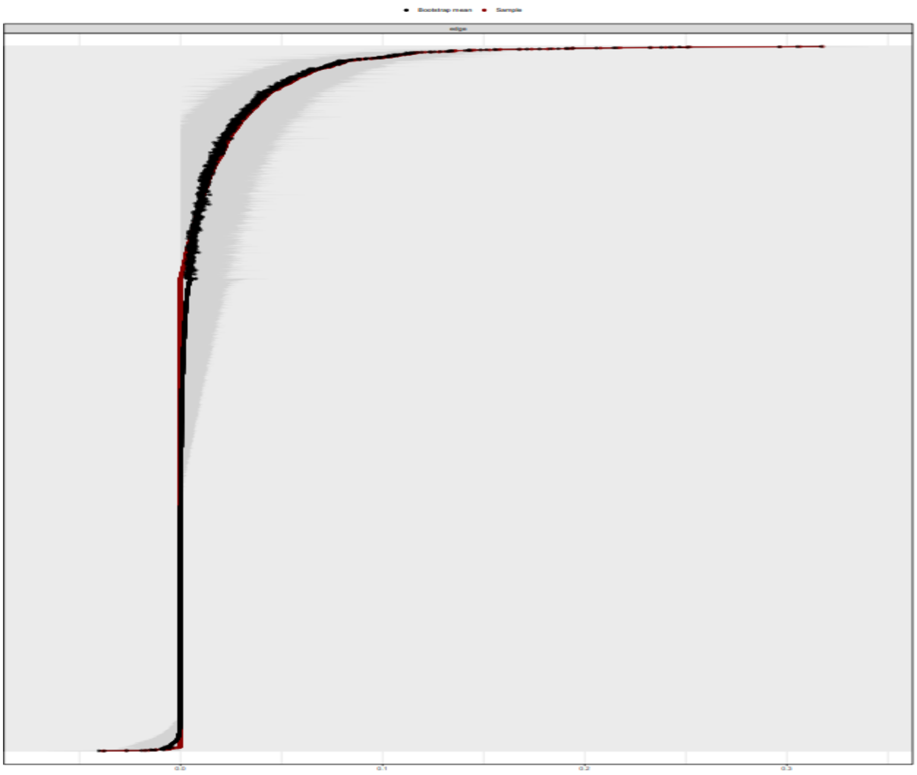

Figure S23 95% CI of rural adolescent network of the entire sample

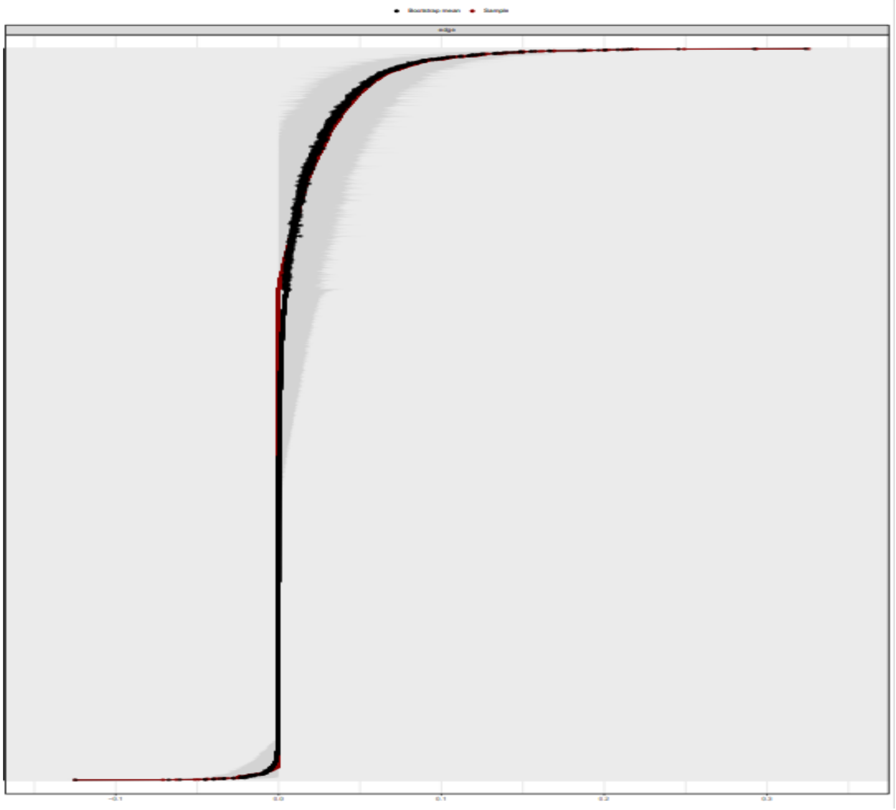

Figure S24 95% CI of urban adolescent network of the entire sample

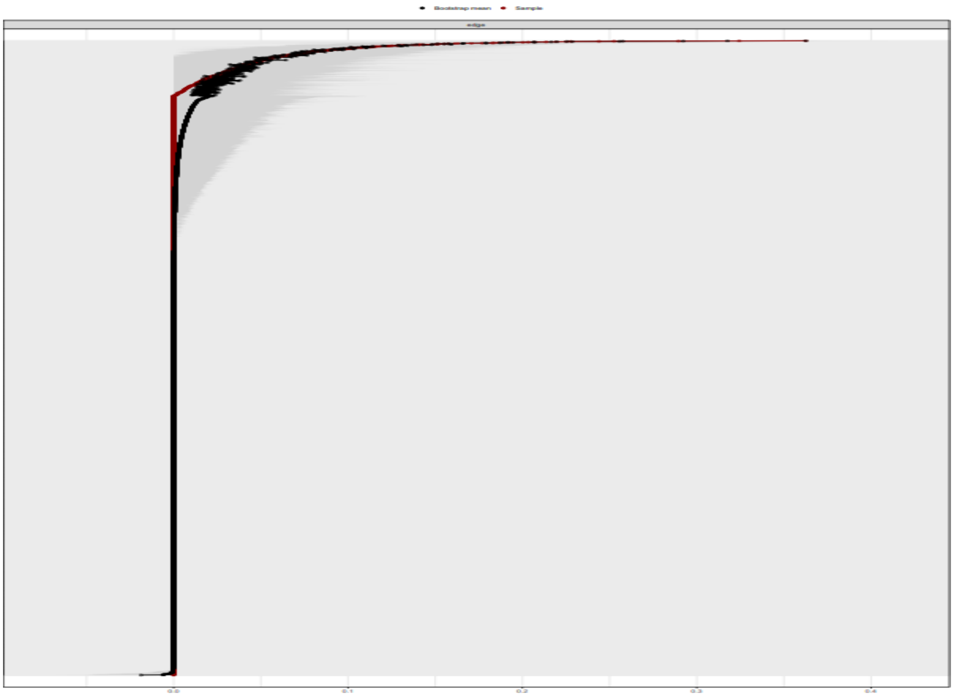

Figure S25   95% CI of rural children network of the mental disorder

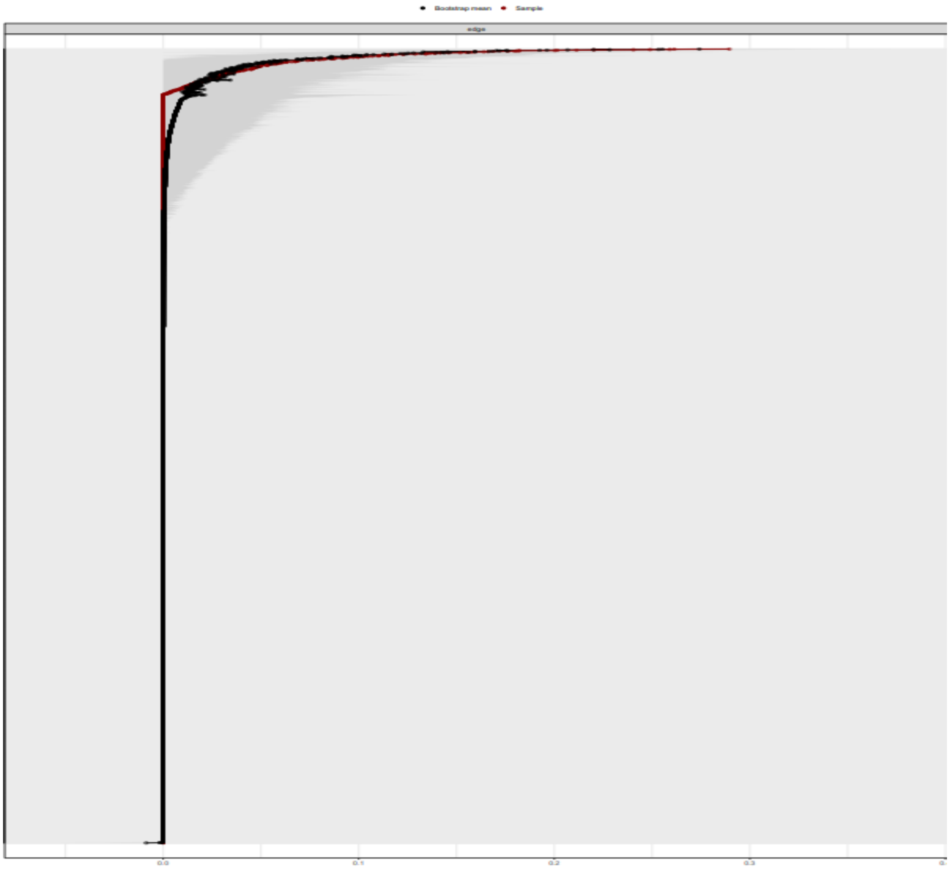

Figure S26   95% CI of urban children network of the mental disorder

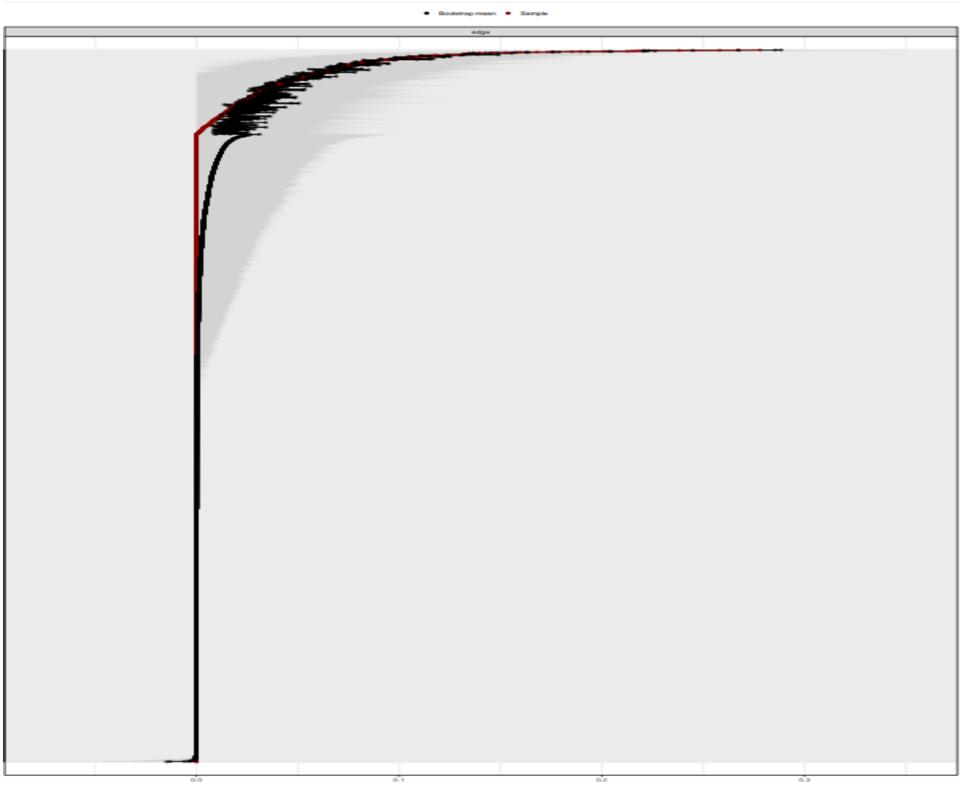

Figure S27   95% CI of rural adolescent network of the mental disorder

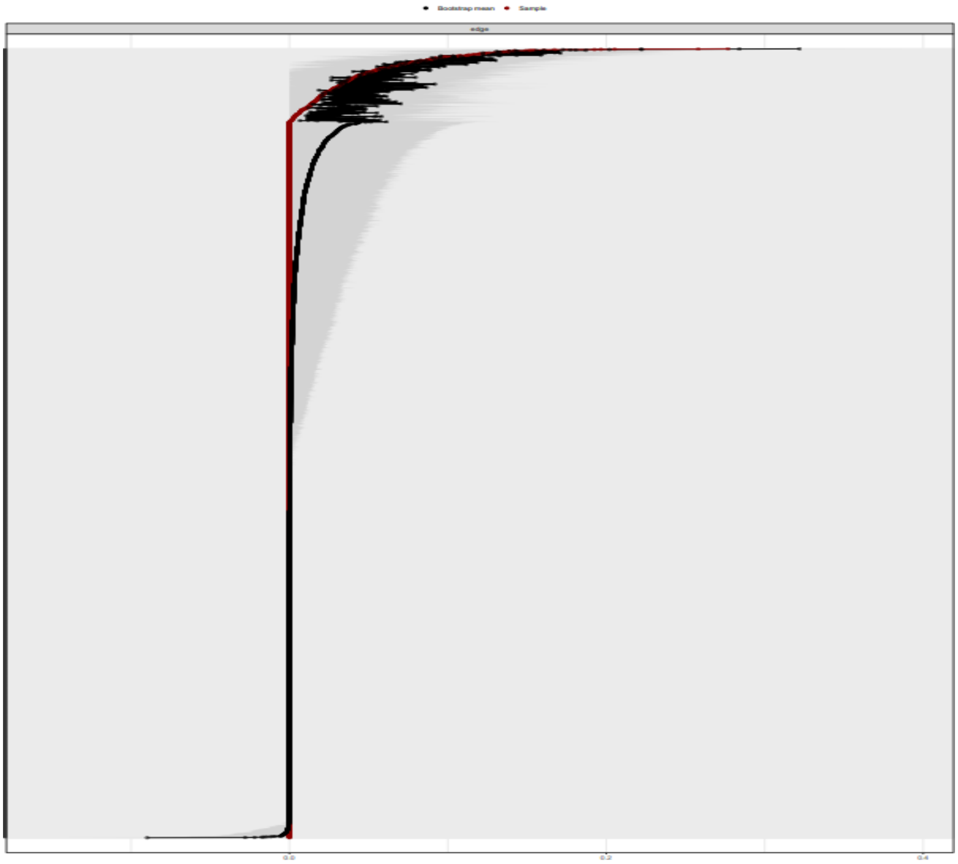

Figure S28   95% CI of urban adolescent network of the mental disorder

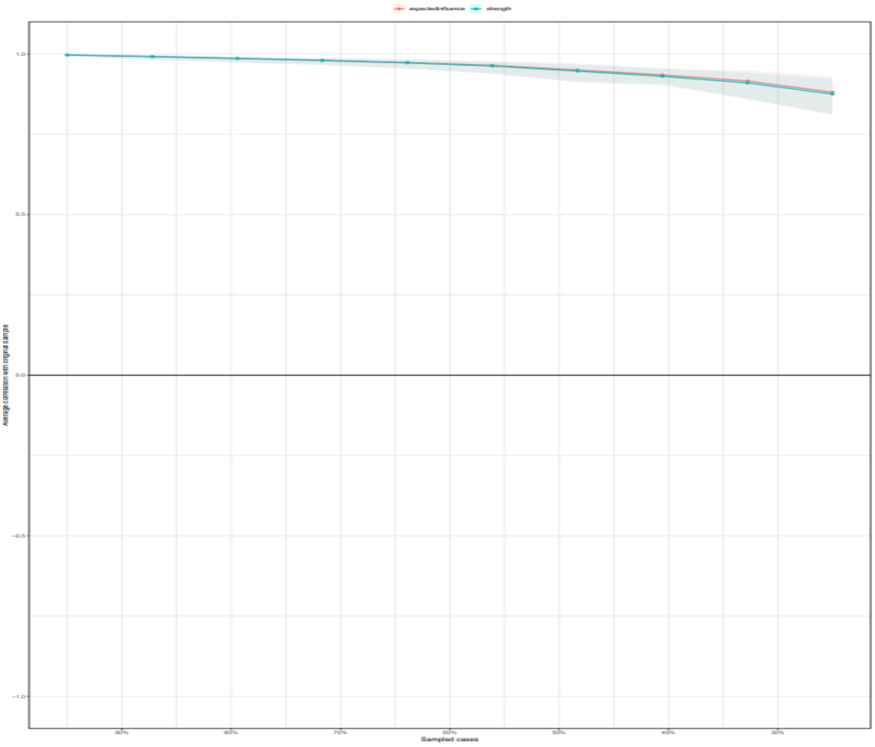

Figure S29　Stability of rural children network of entire sample

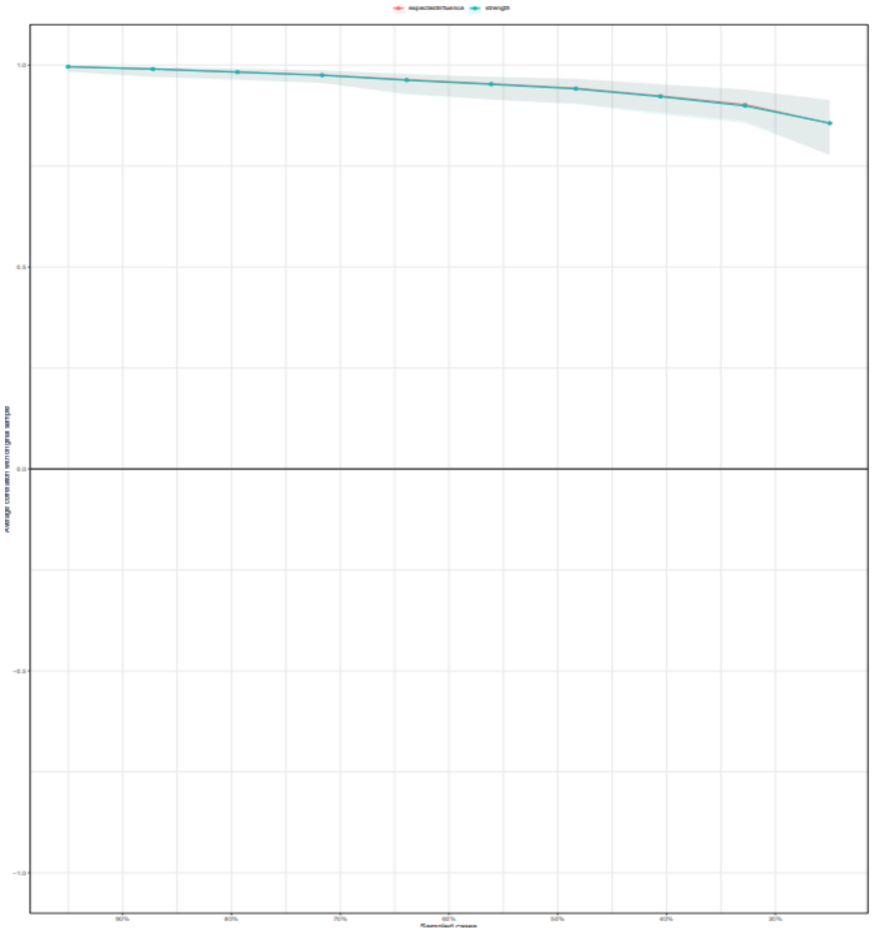

Figure S30　Stability of urban children of entire sample

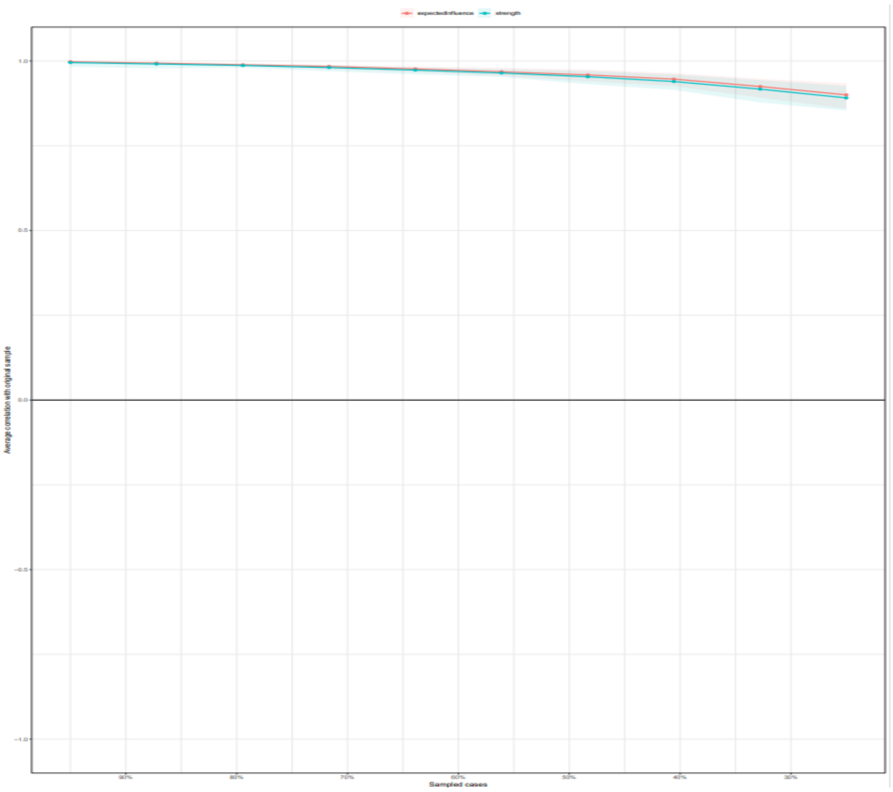

Figure S31 Stability of rural adolescent network of entire sample

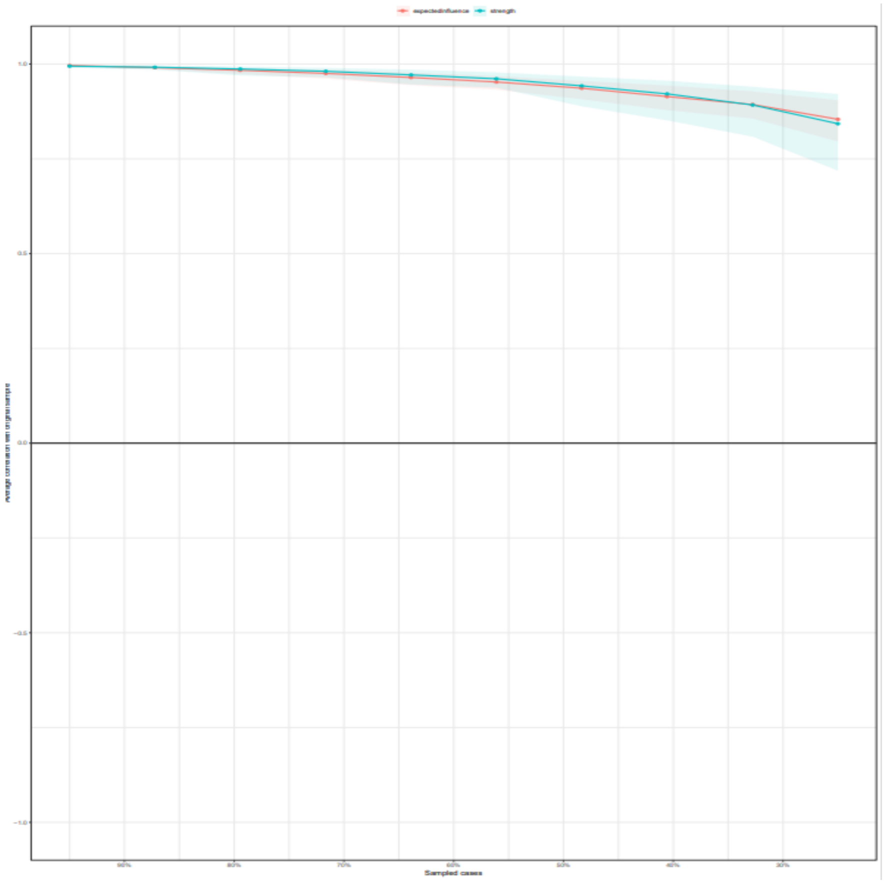

Figure S32 Stability of urban adolescent of entire sample

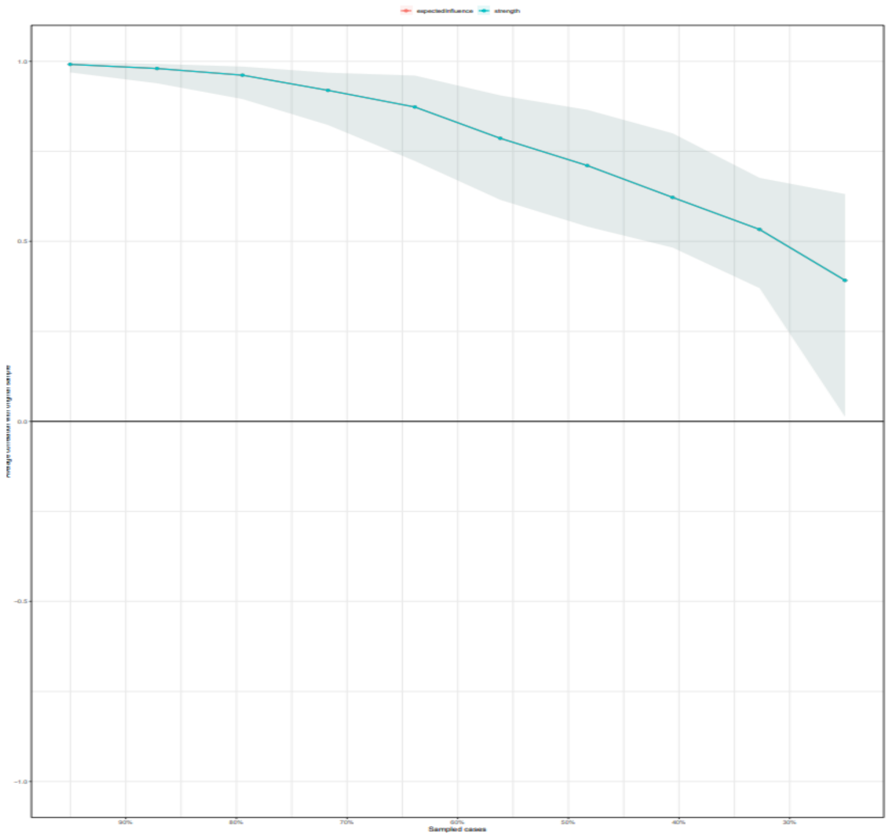

Figure S33 Stability of rural children network of mental disorder

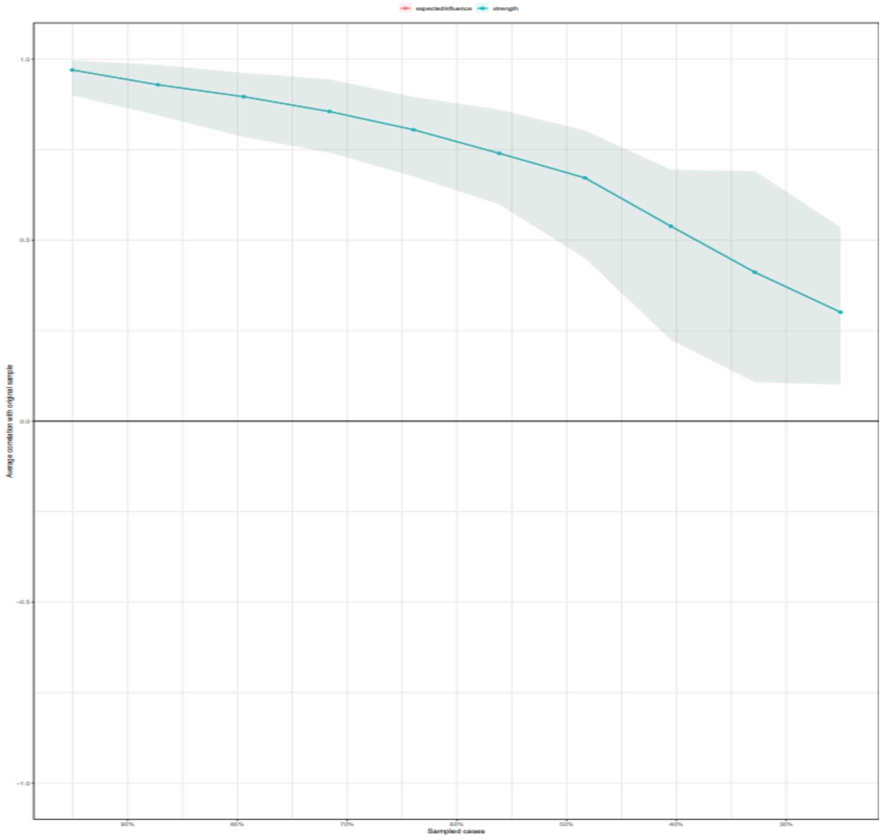

Figure S34 Stability of urban children of mental disorder

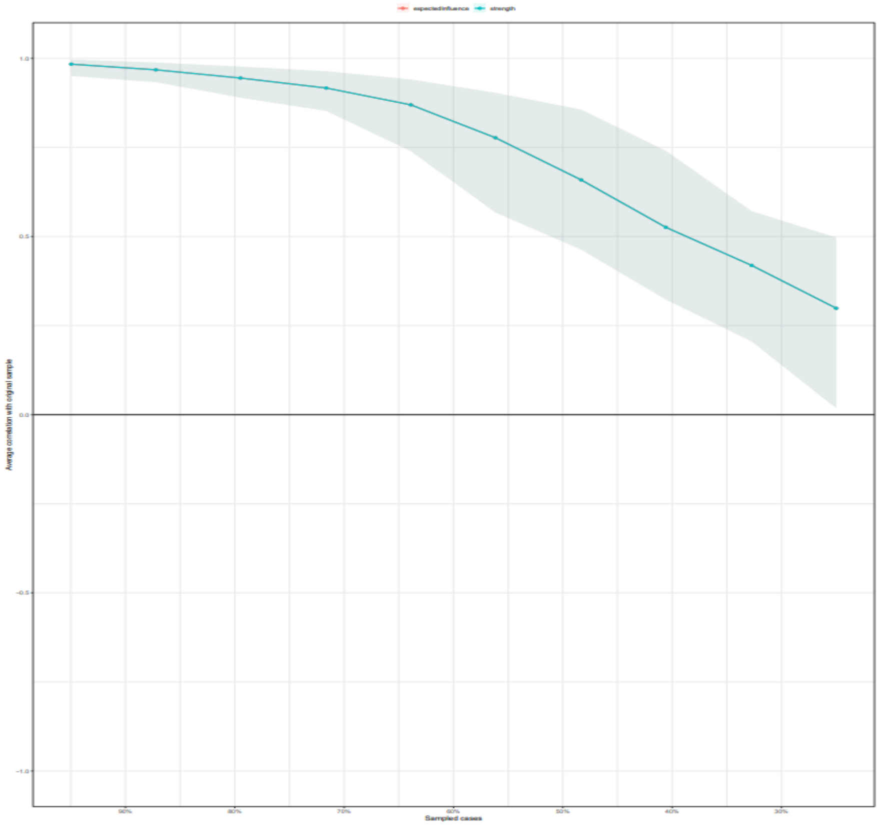

Figure S35 Stability of rural adolescent network of mental disorder

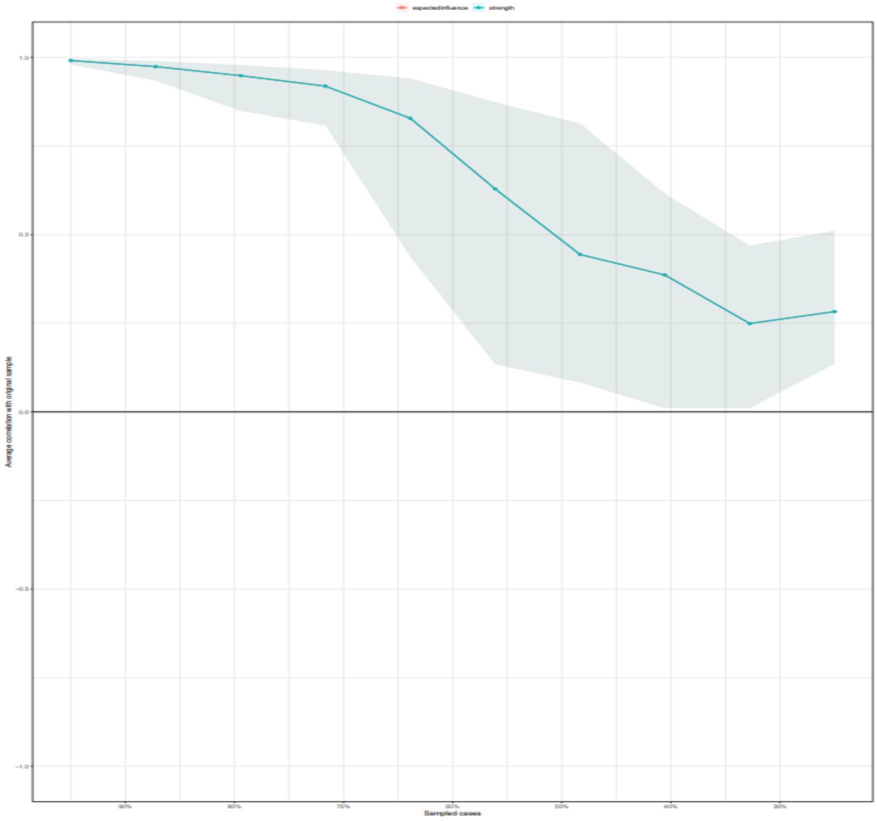

Figure S36 Stability of urban adolescent of mental disorder

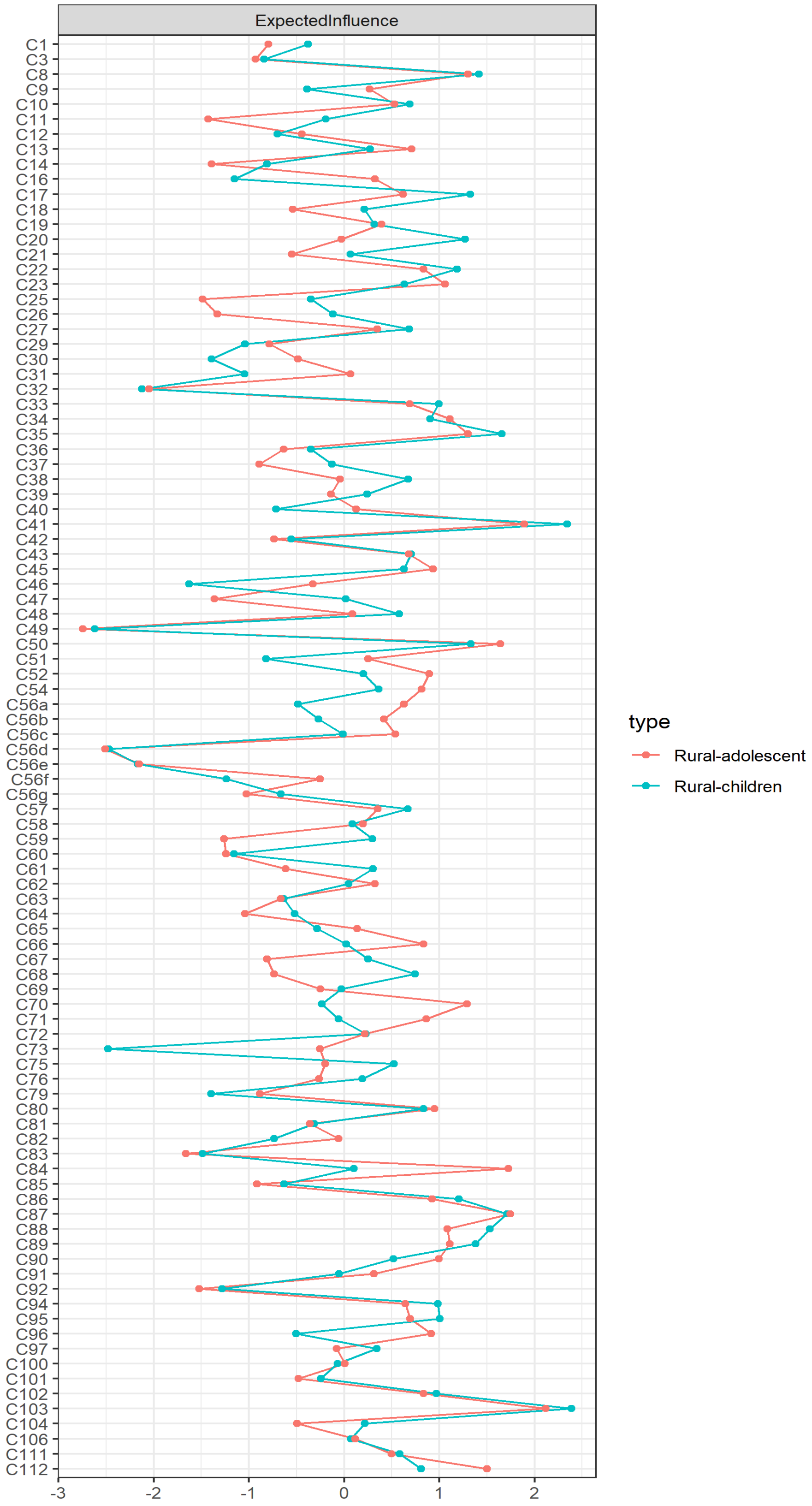

Figure S37 Centrality indicators of rural children and adolescents of entire sample

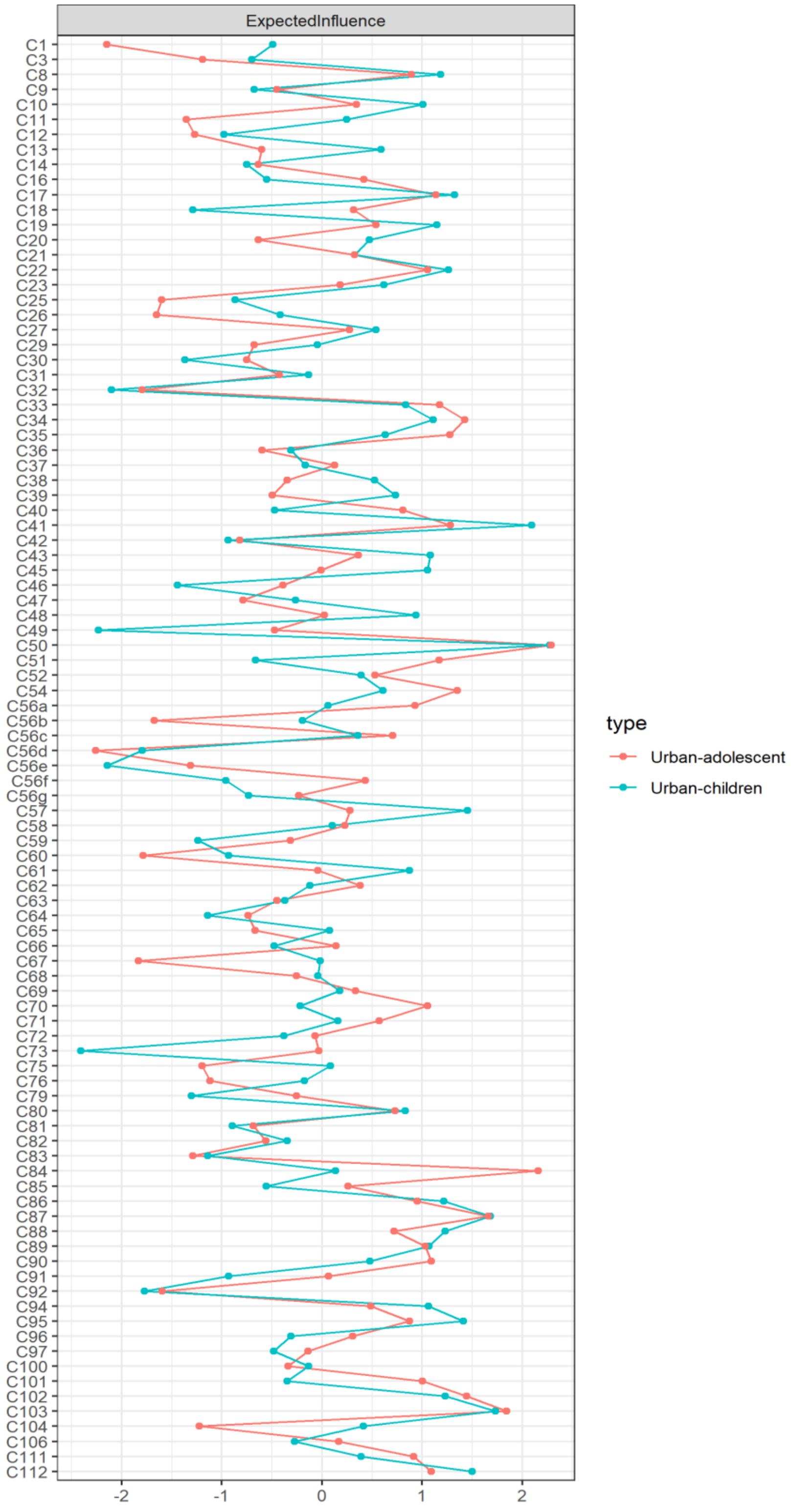

Figure S38 Centrality indicators of urban children and adolescents of entire sample

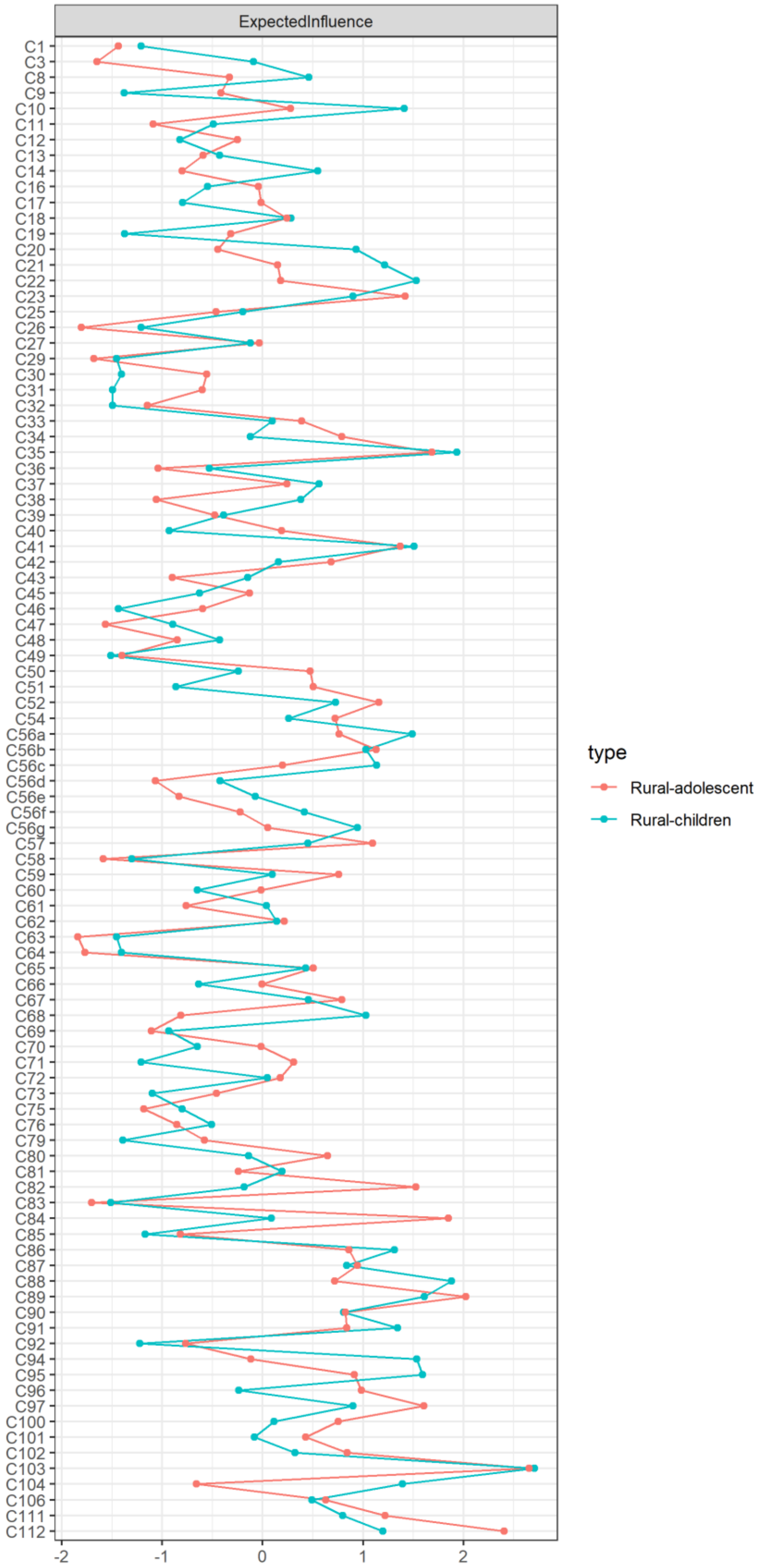

Figure S39 Centrality indicators of rural children and adolescents of mental disorder

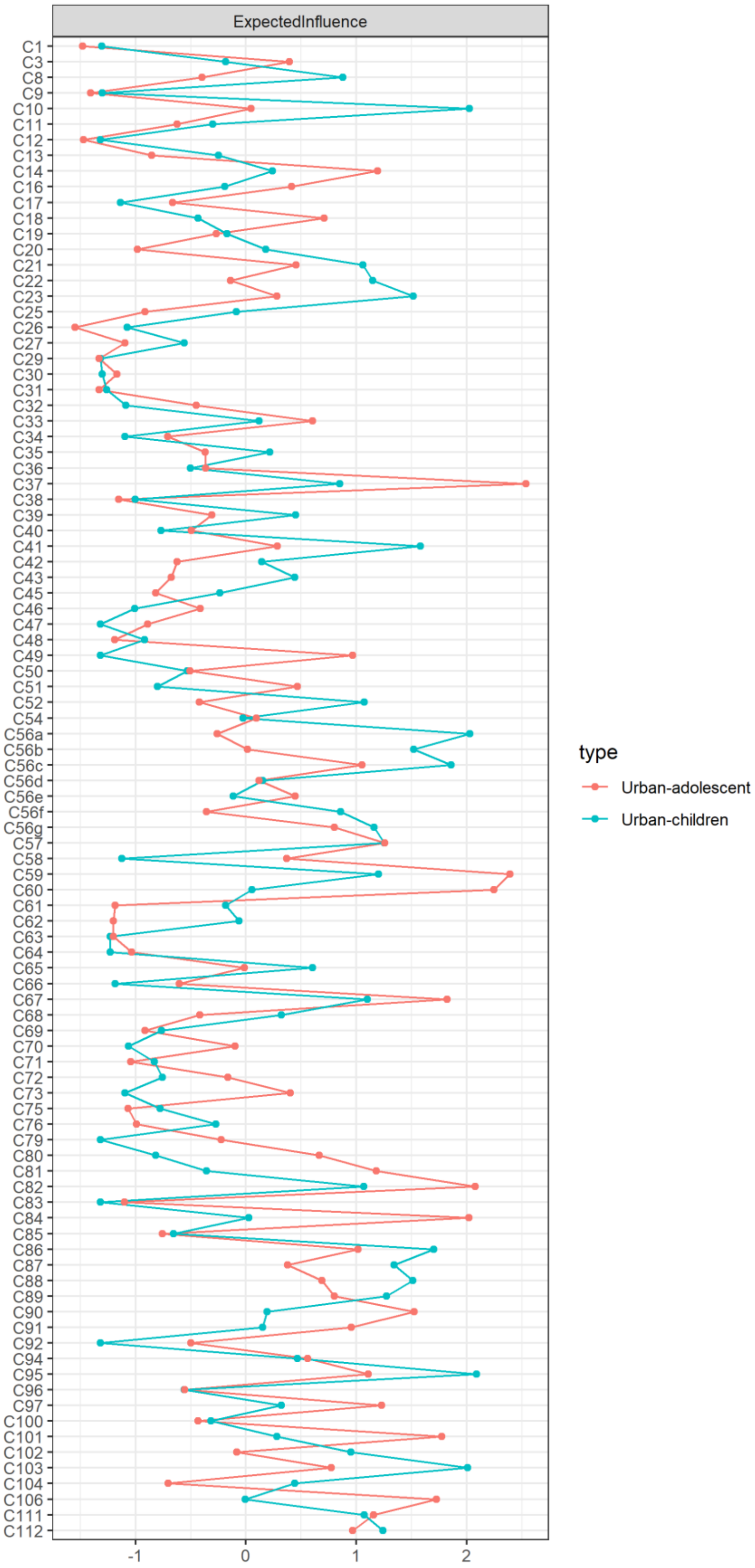

Figure S40 Centrality indicators of urban children and adolescents of mental disorder

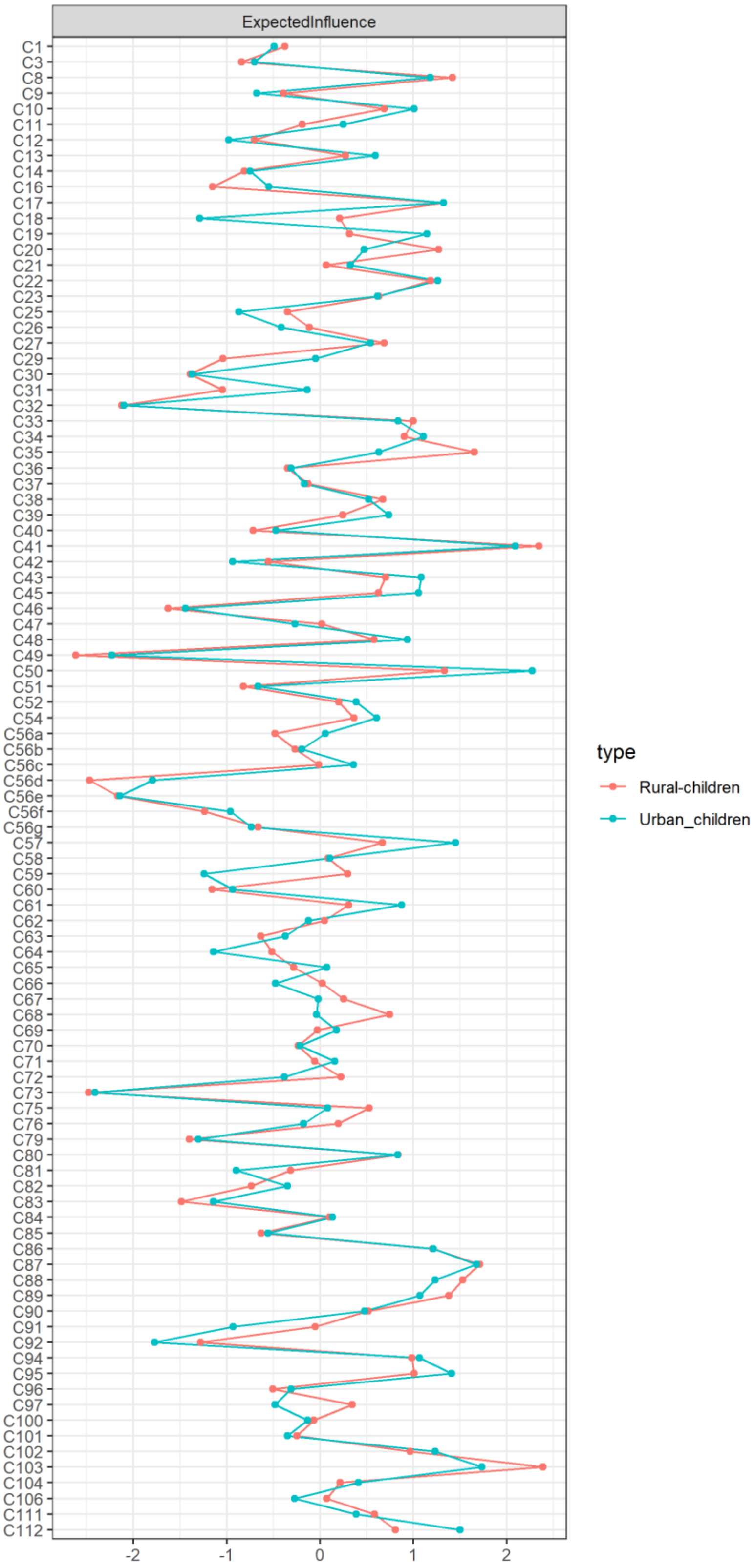

Figure S41 Centrality indicators of rural children and urban children of entire sample

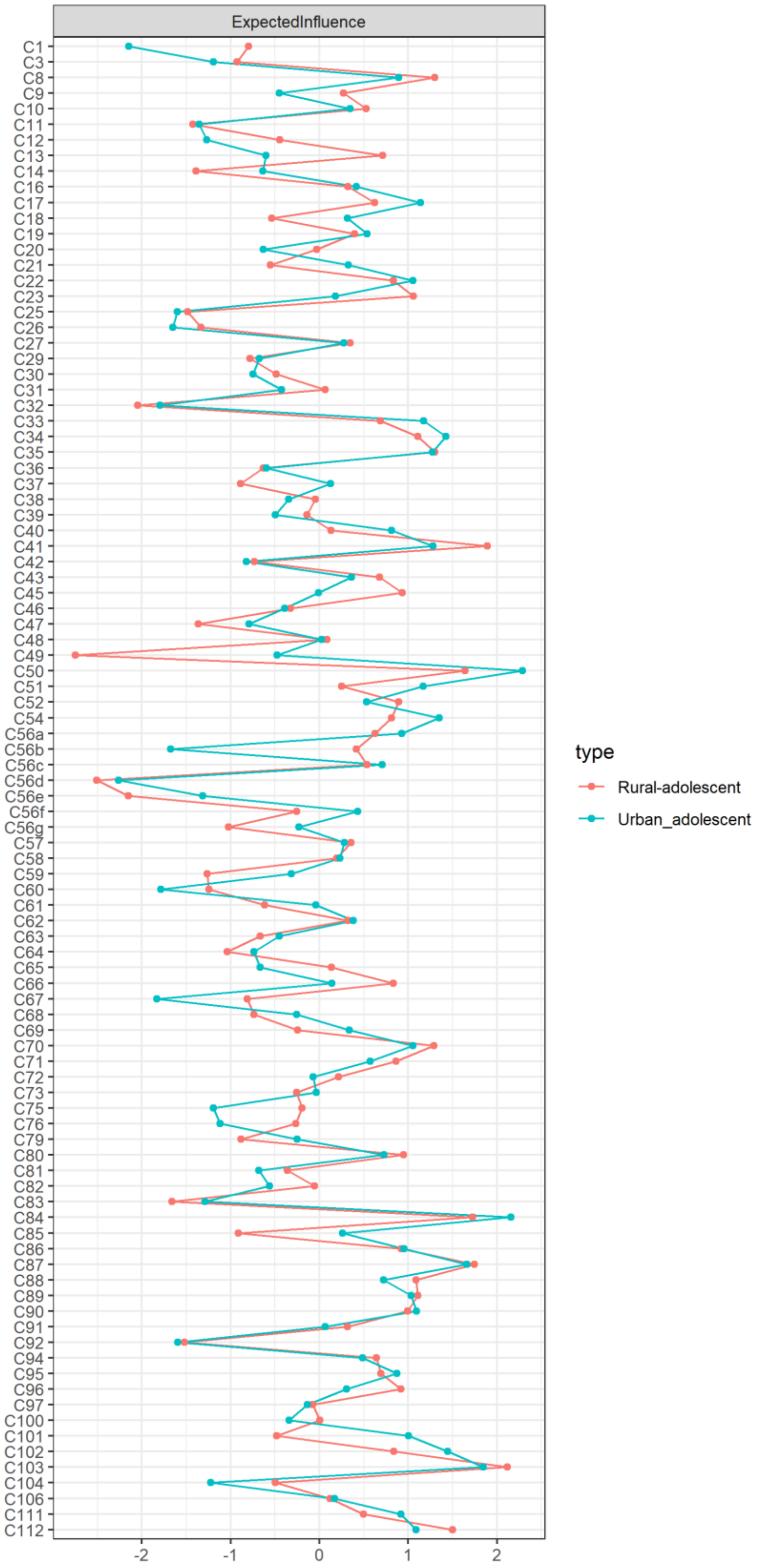

Figure S42 Centrality indicators of rural adolescent and urban adolescent of entire sample

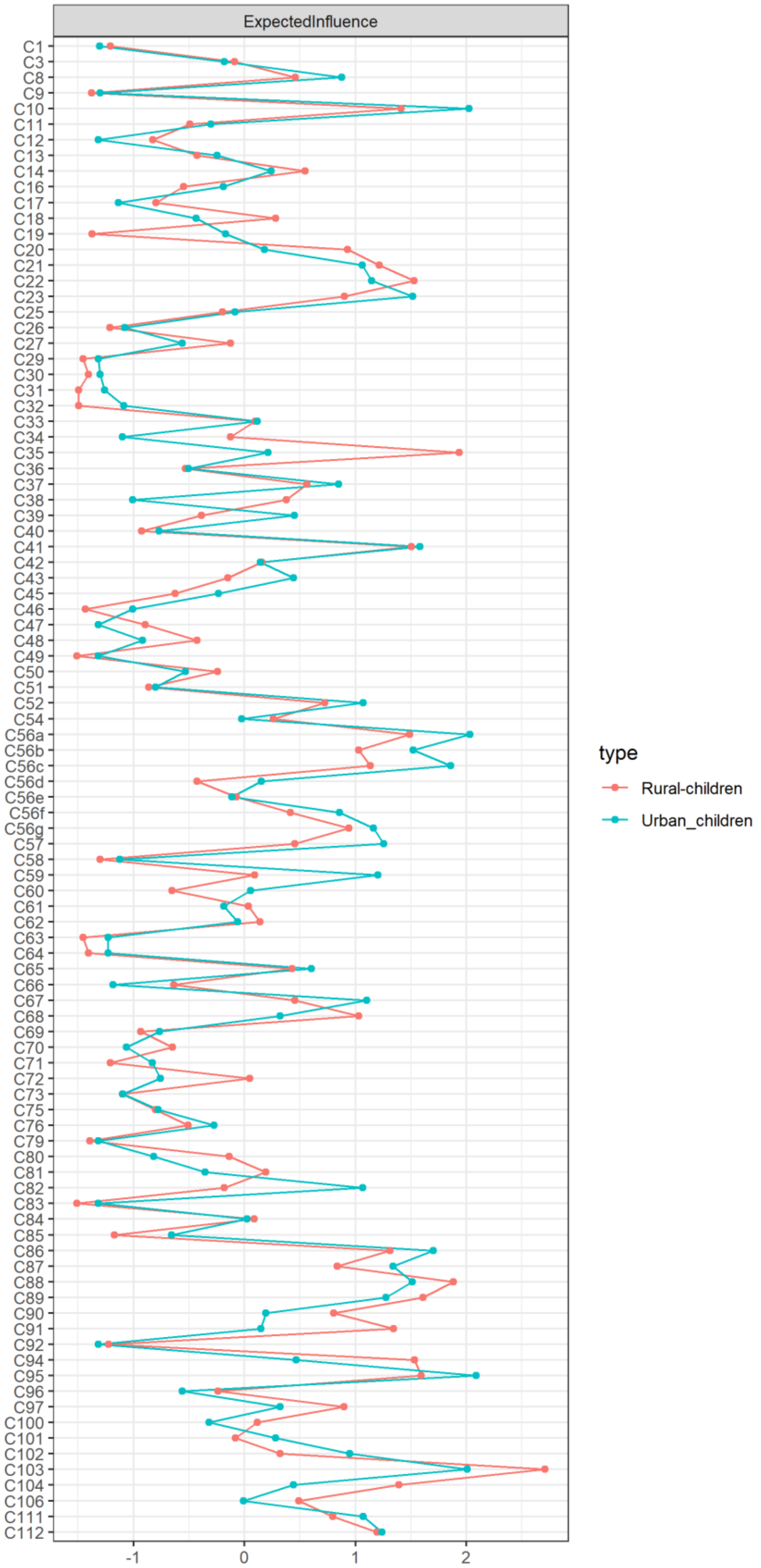

Figure S43 Centrality indicators of rural children and urban children of mental disorder

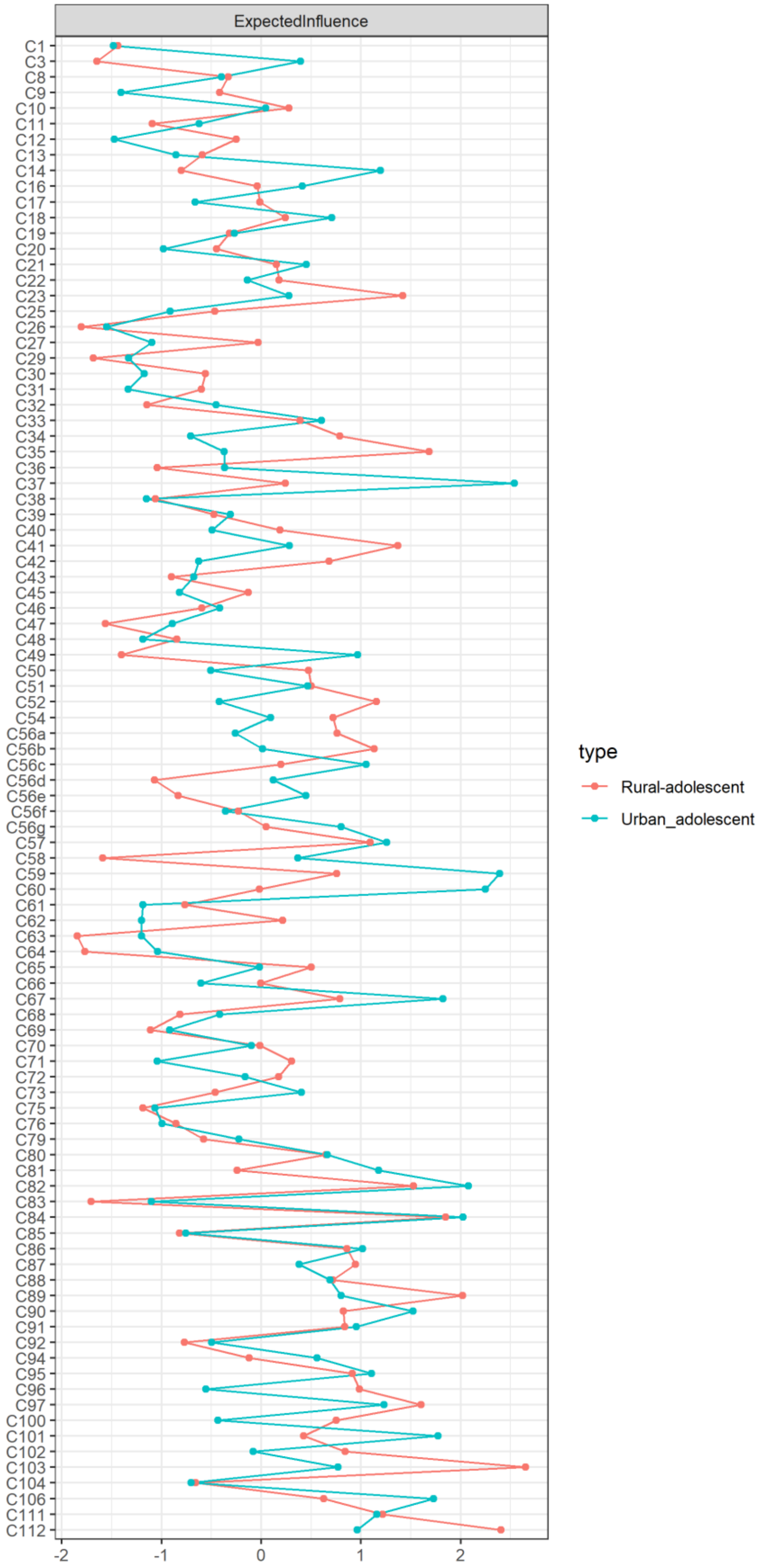

Figure S44 Centrality indicators of rural adolescent and urban adolescent of mental disorder

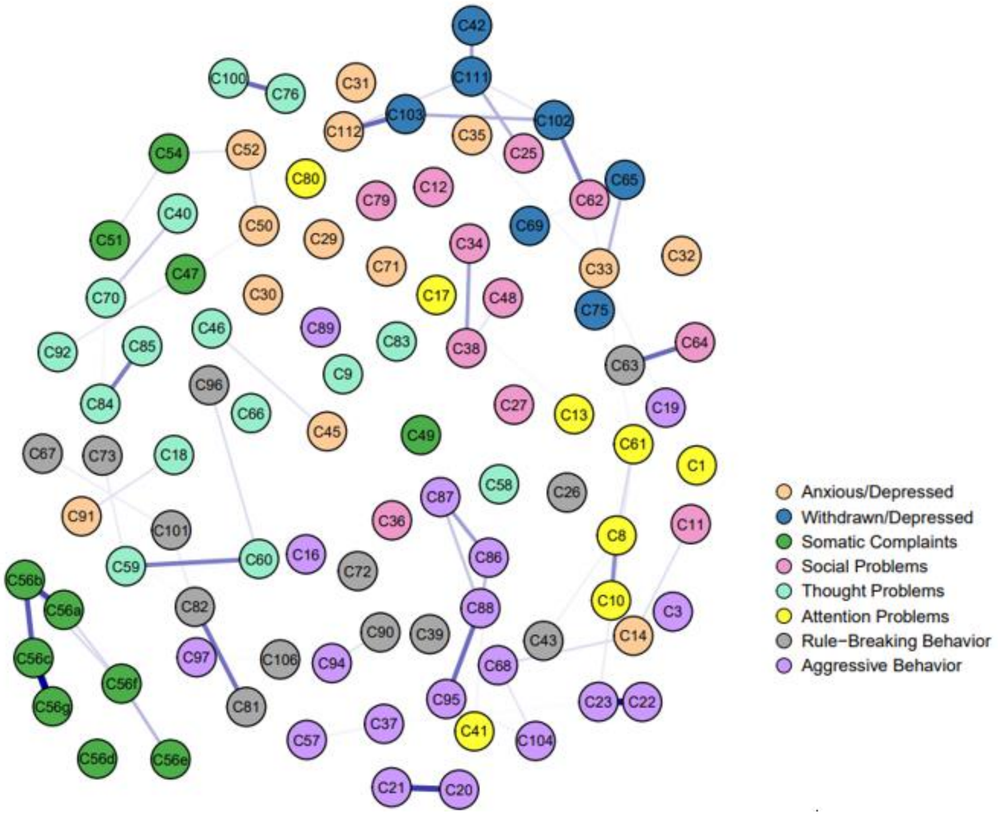

Figure S45 Networks of rural boy groups of the entire sample

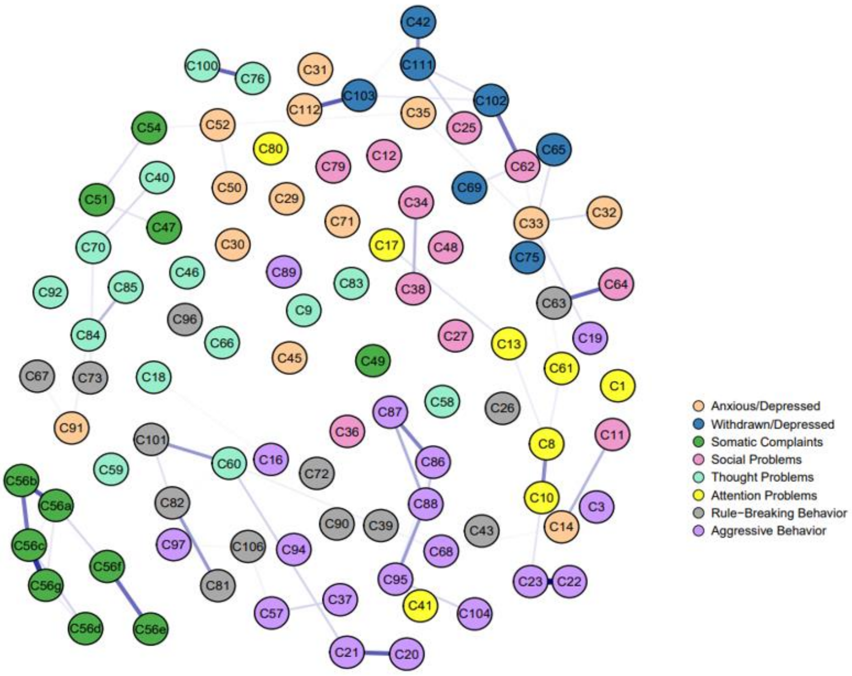

Figure S46 Networks of urban boy groups of the entire sample

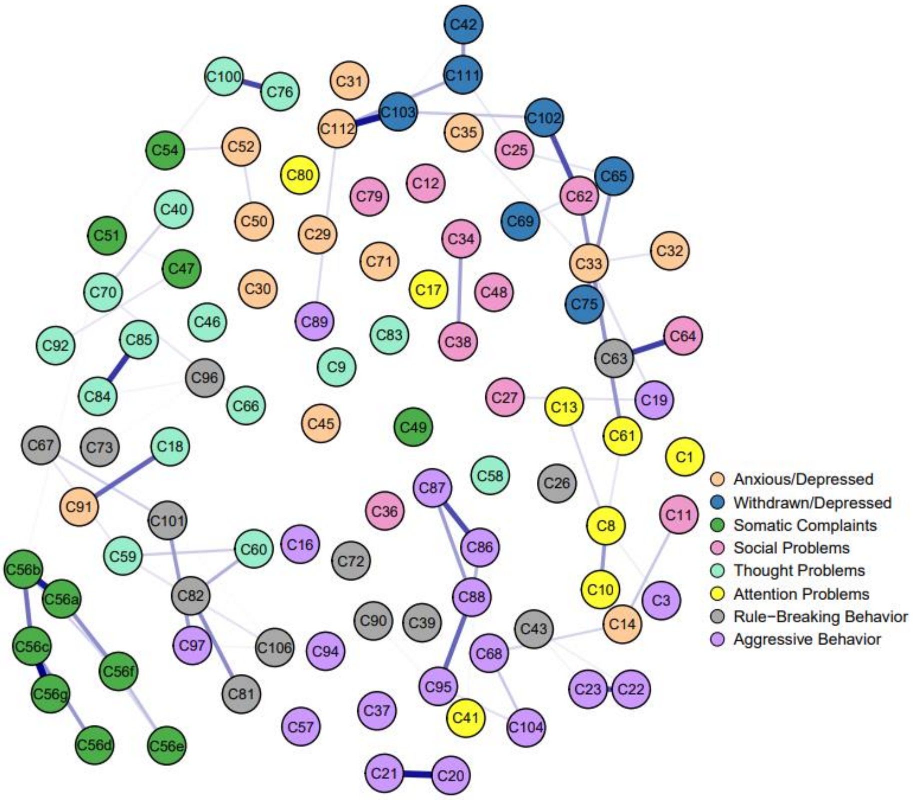

Figure S47 Networks of rural girl groups of the entire sample

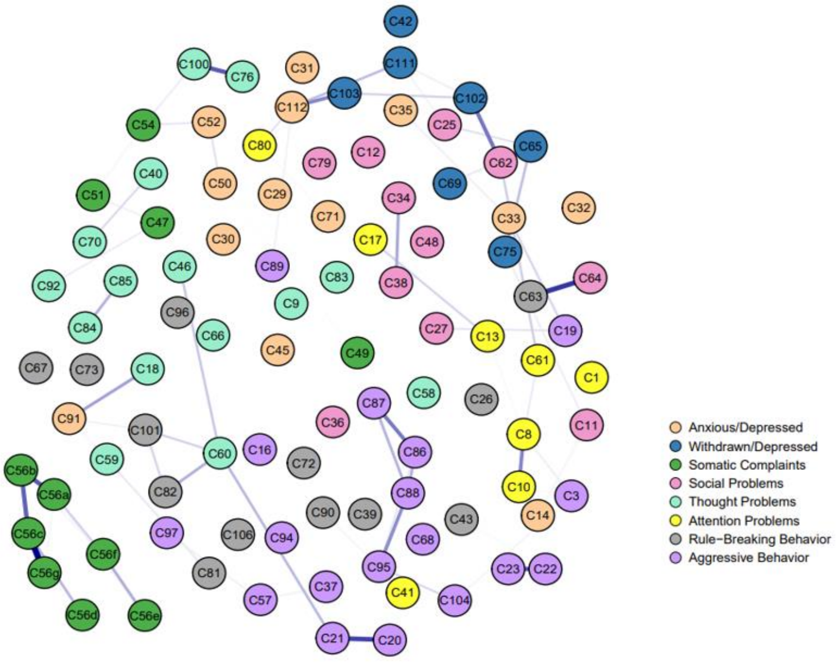

Figure S48 Networks of urban girl groups of the entire sample

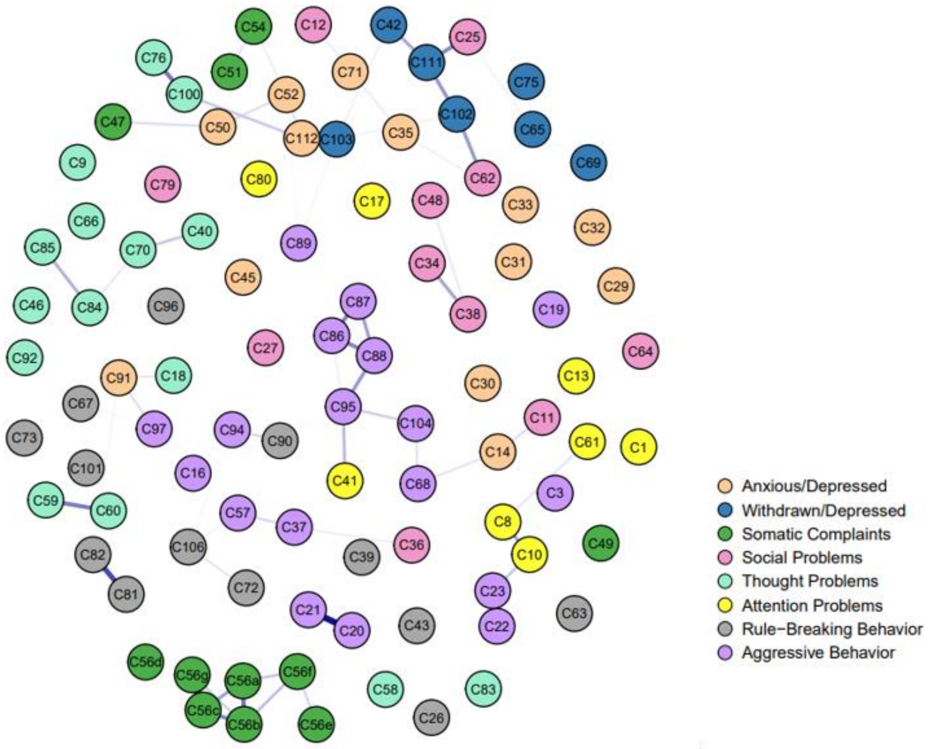

Figure S49 Networks of rural boy groups of the mental disorder

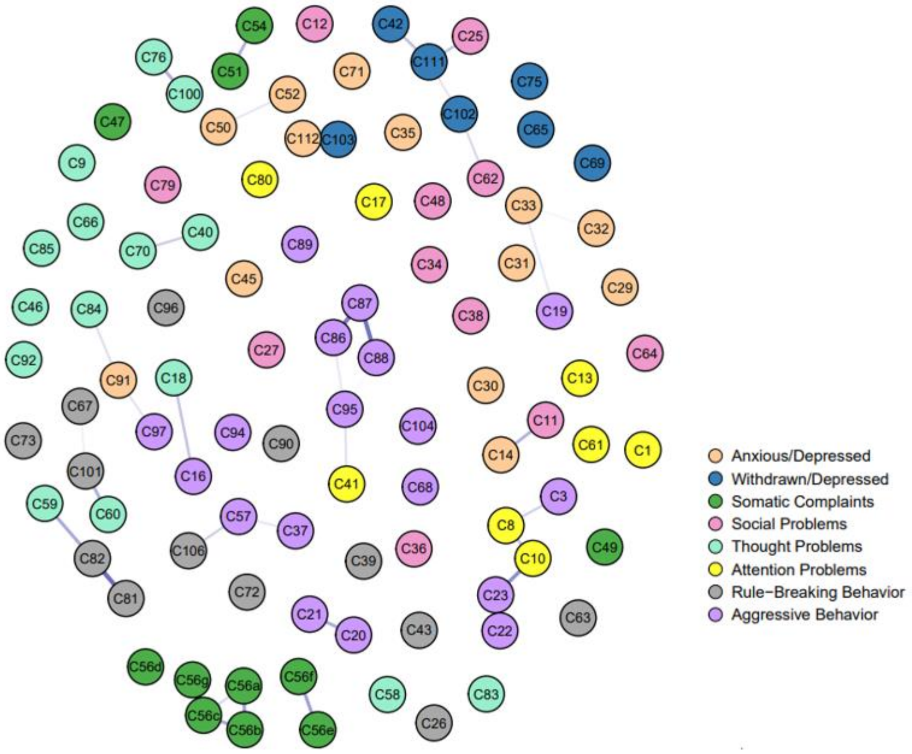

Figure S50 Networks of urban boy groups of the mental disorder

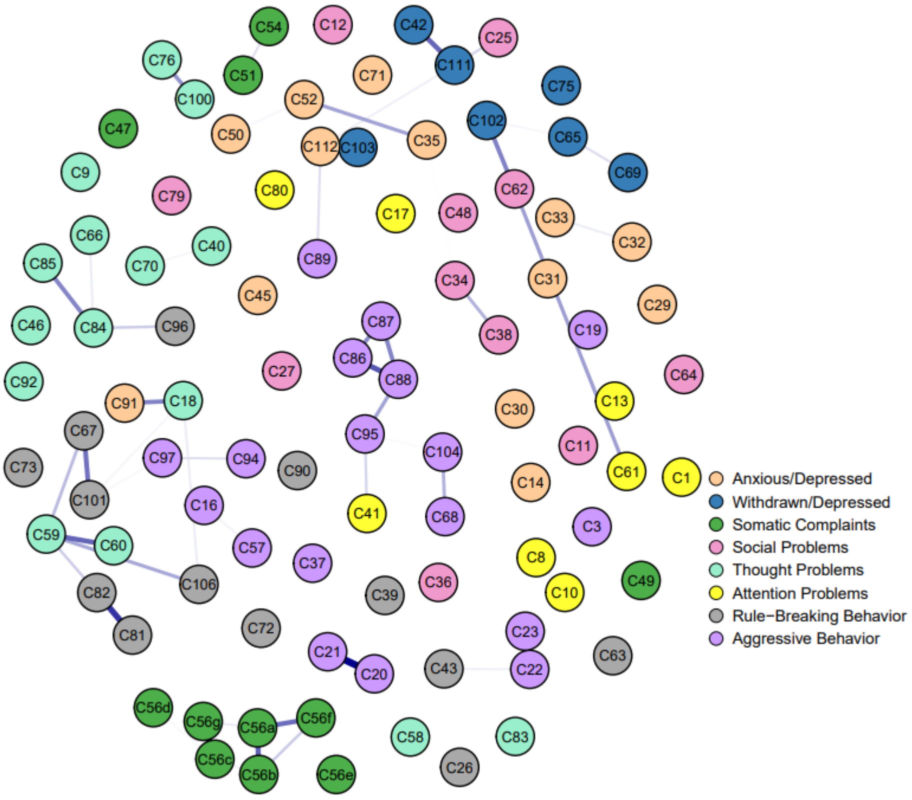

Figure S51 Networks of rural girl groups of the mental disorder

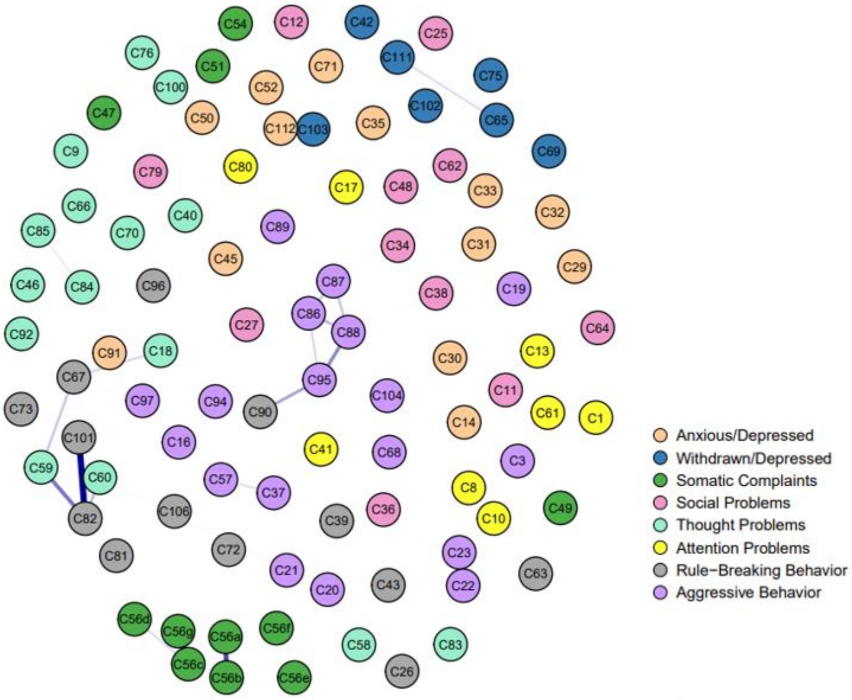

Figure S52 Networks of urban girl groups of the mental disorder

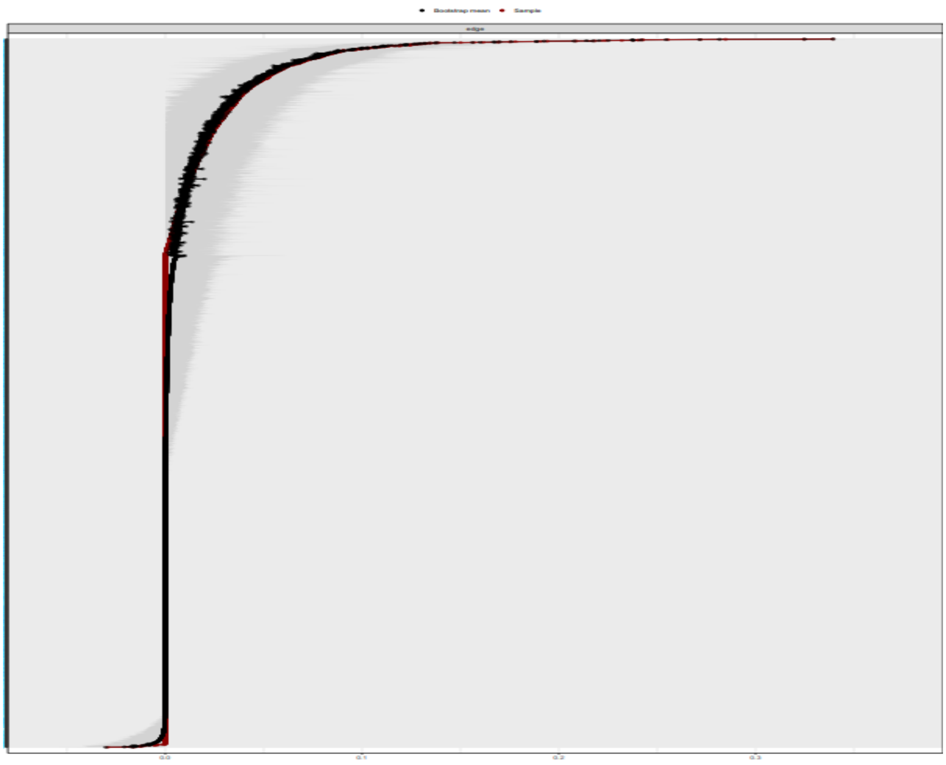

Figure S53 95% CI of rural boy network of the entire sample

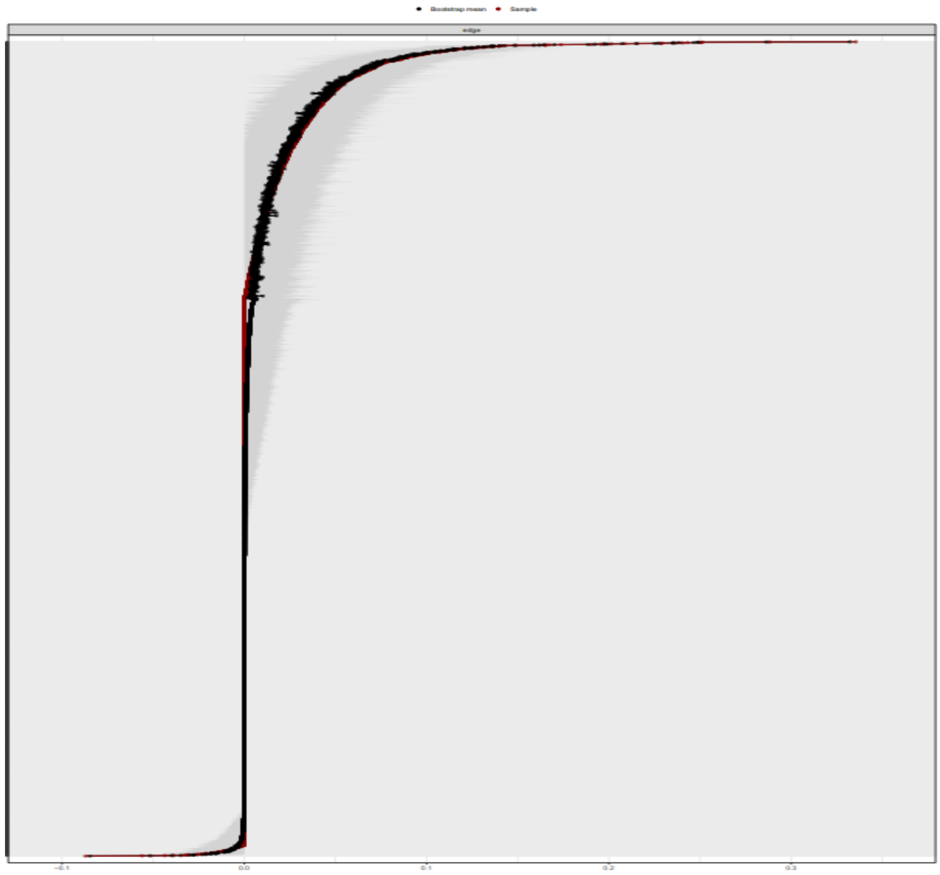

Figure S54 95% CI of urban boy network of the entire sample

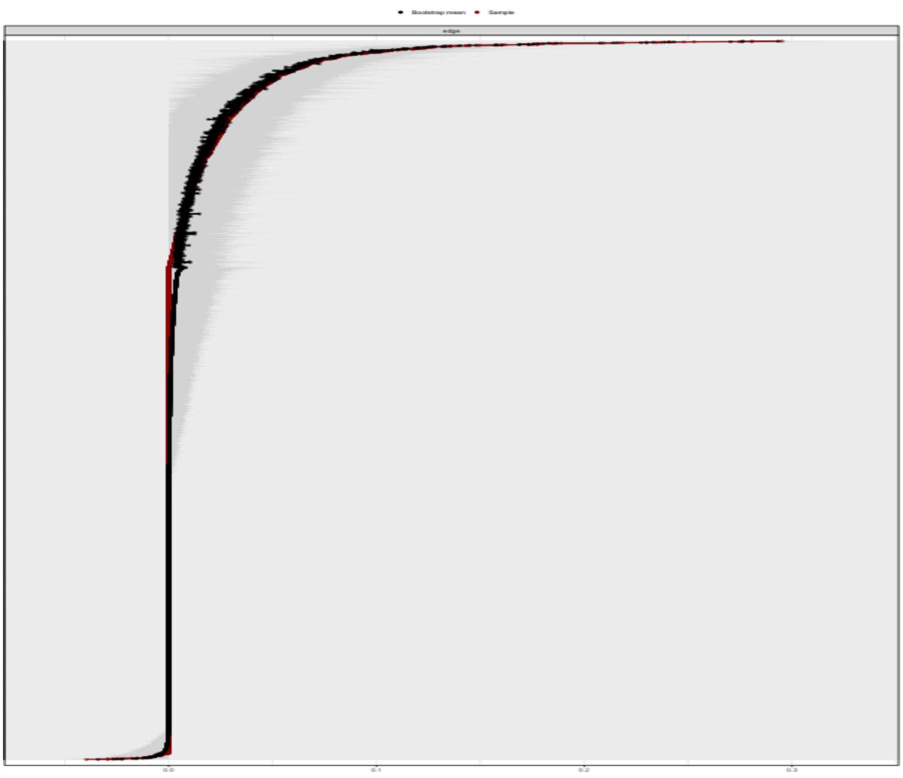

Figure S55   95% CI of rural girl network of the entire sample

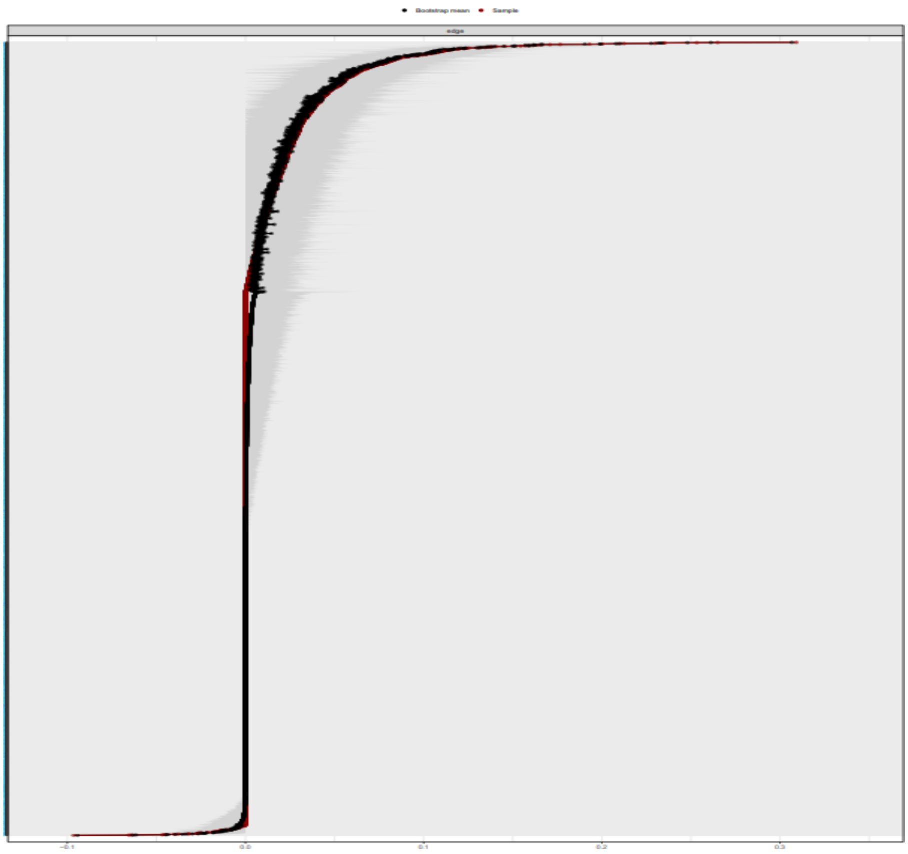

Figure S56   95% CI of urban girl network of the entire sample

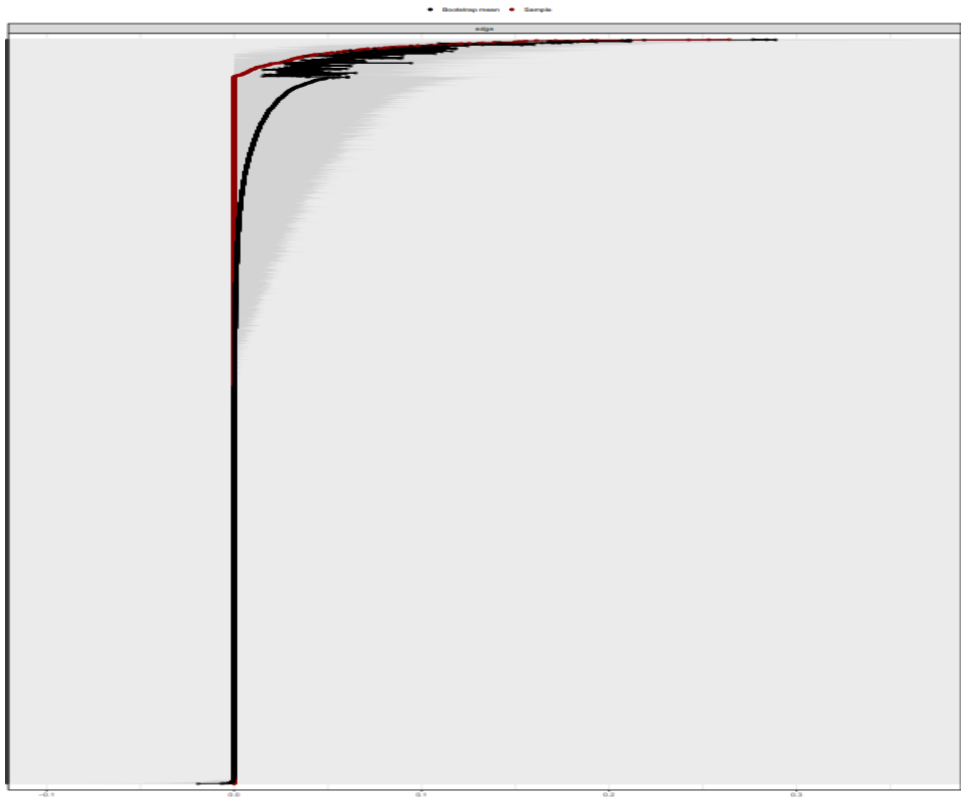

Figure S57   95% CI of rural boy network of the mental disorder

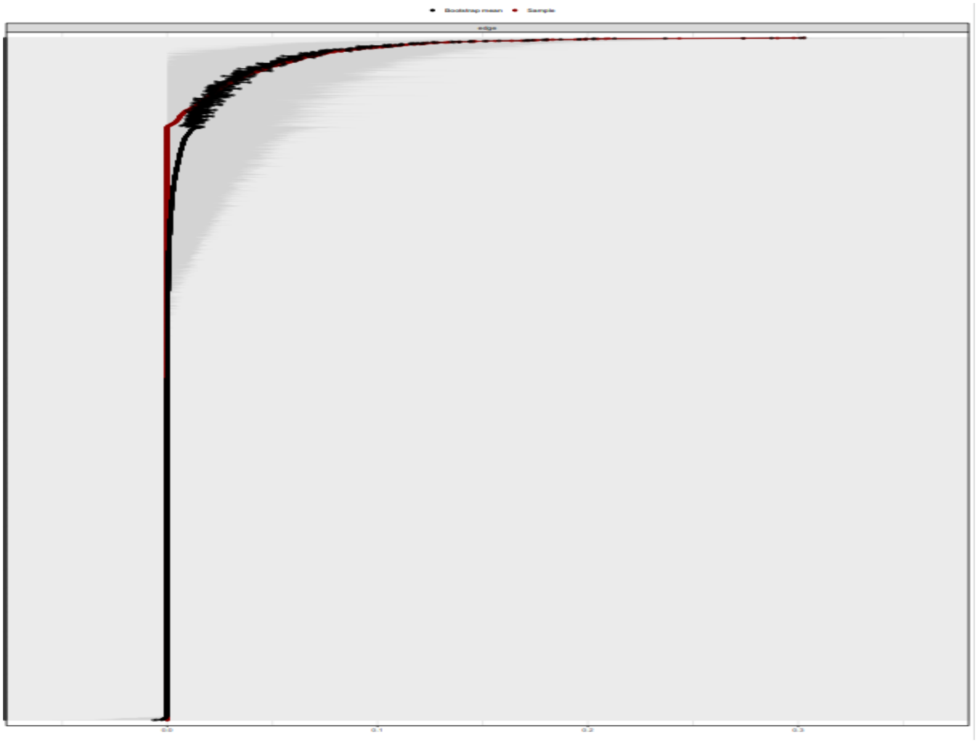

Figure S58   95% CI of urban boy network of the mental disorder

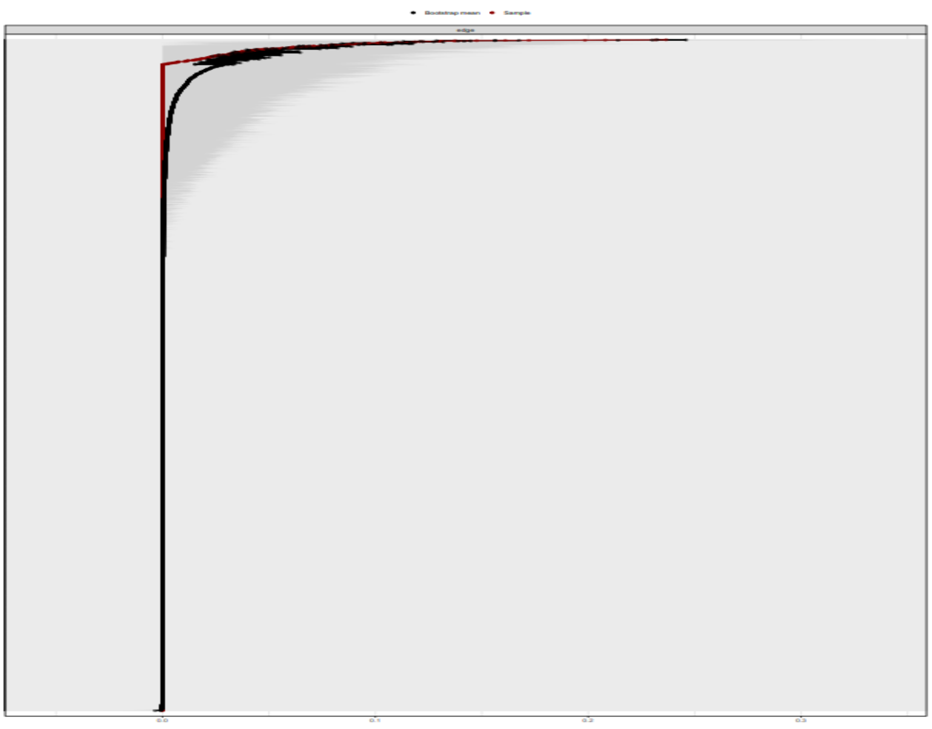

Figure S59   95% CI of rural girl network of the mental disorder

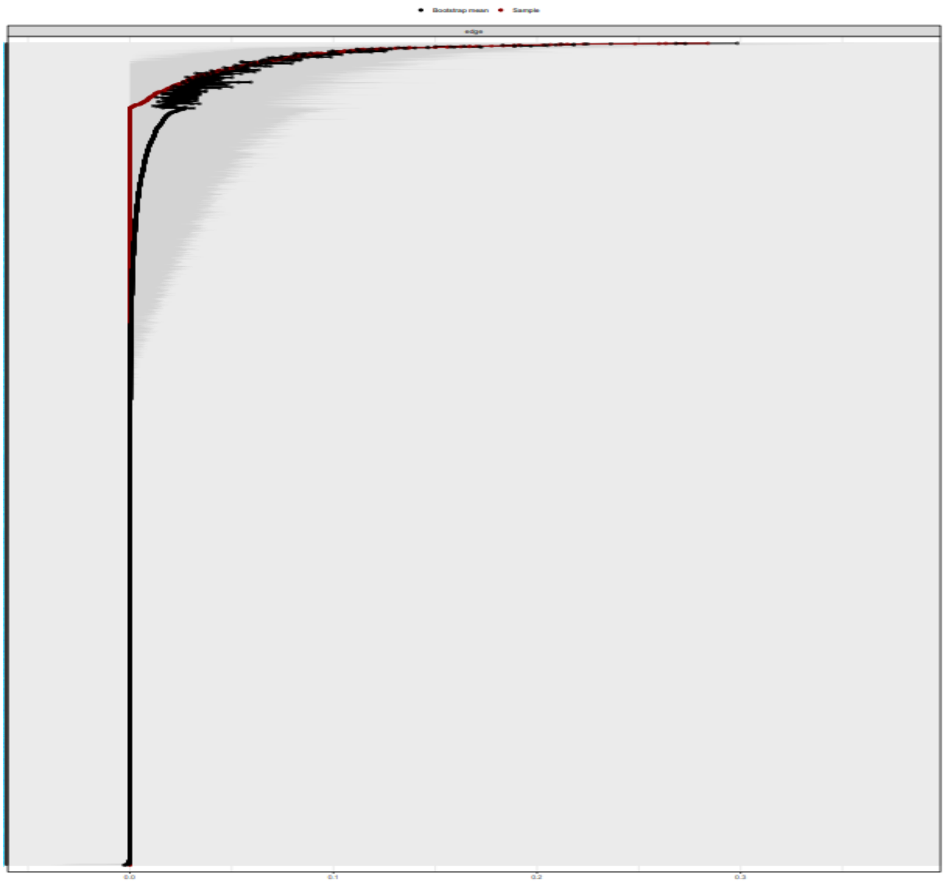

Figure S60   95% CI of urban girl network of the mental disorder

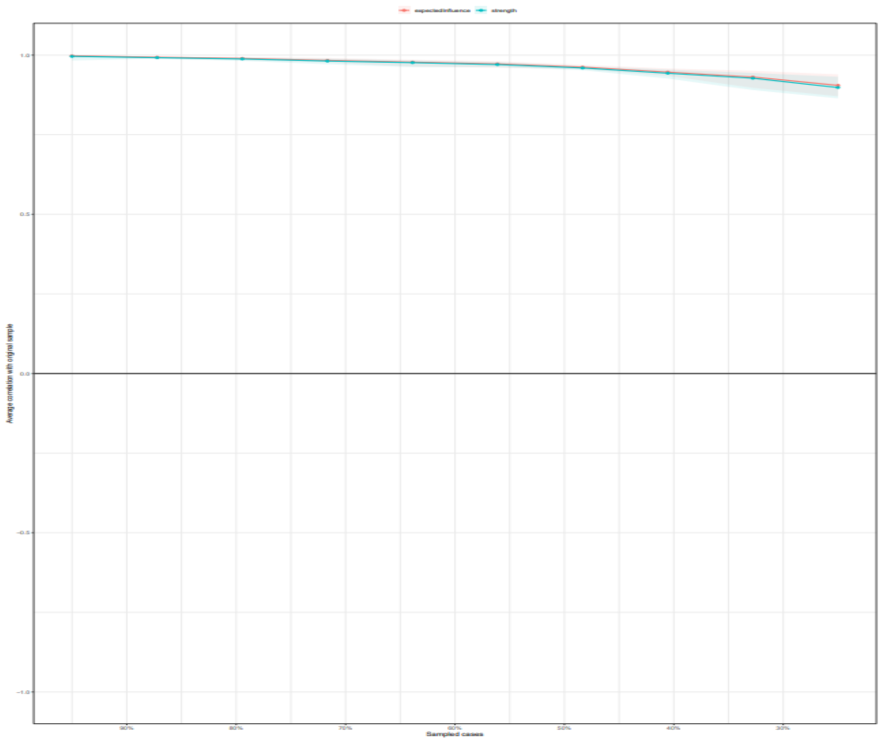

Figure S61　Stability of rural boy network of entire sample

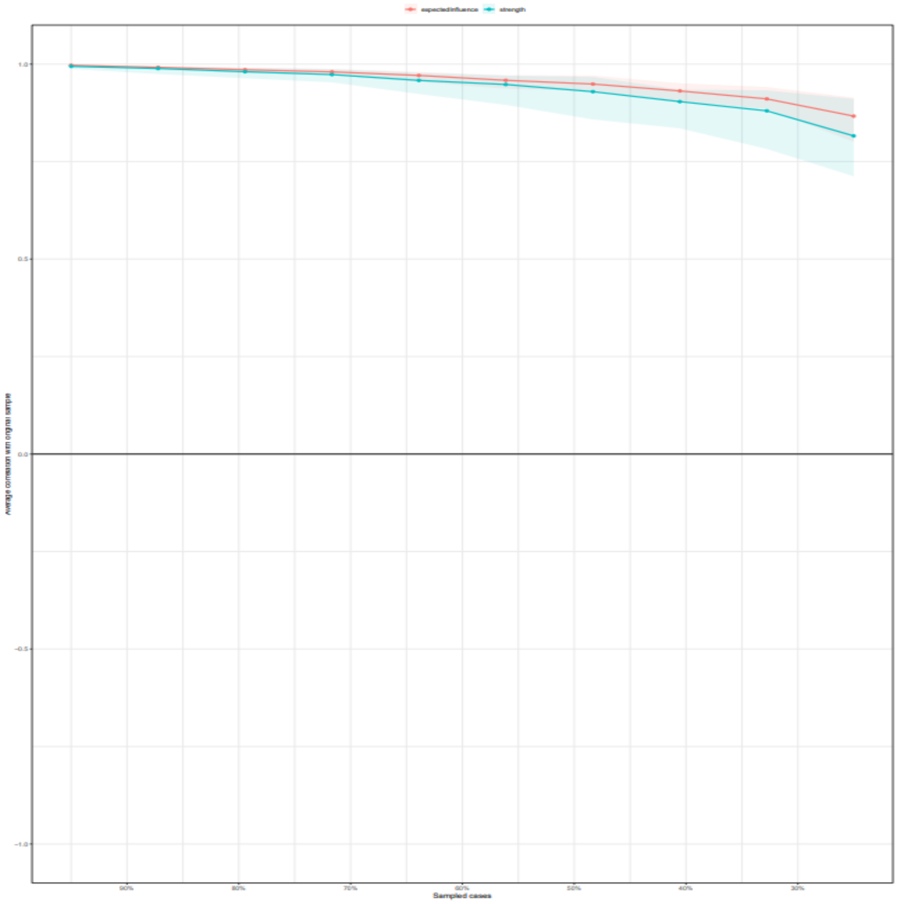

Figure S62　Stability of urban boy of entire sample

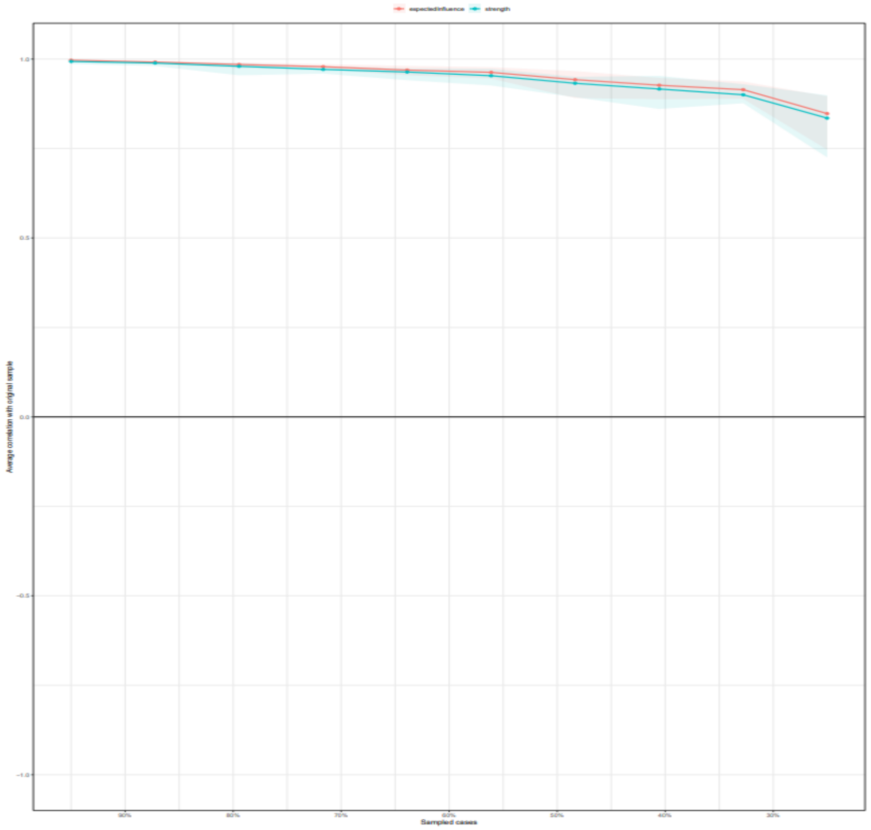

Figure S63　Stability of rural girl network of entire sample

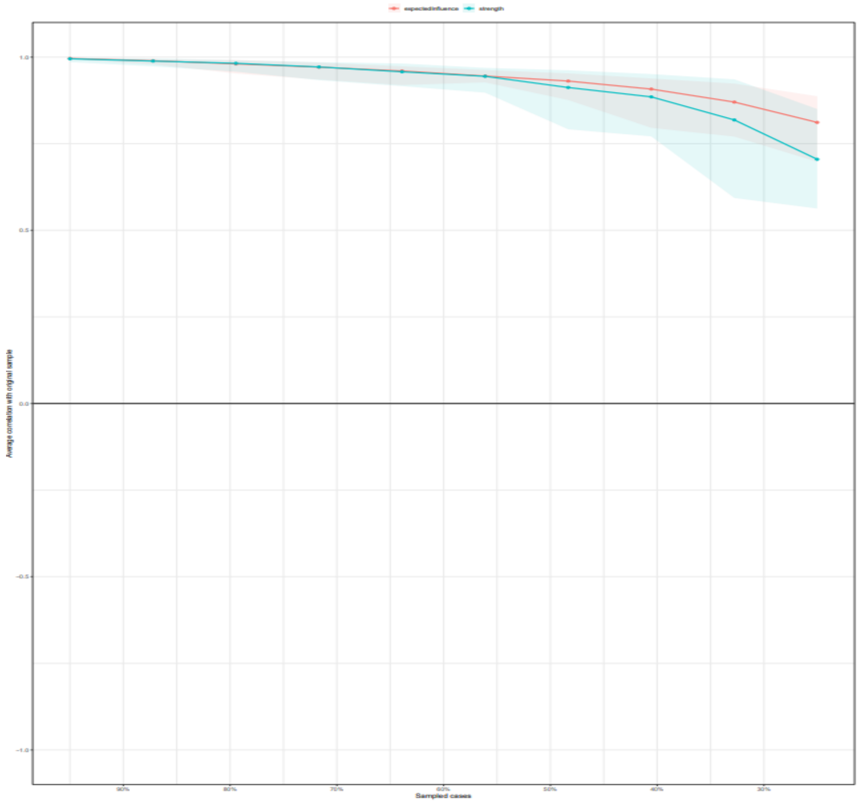

Figure S64　Stability of urban girl of entire sample

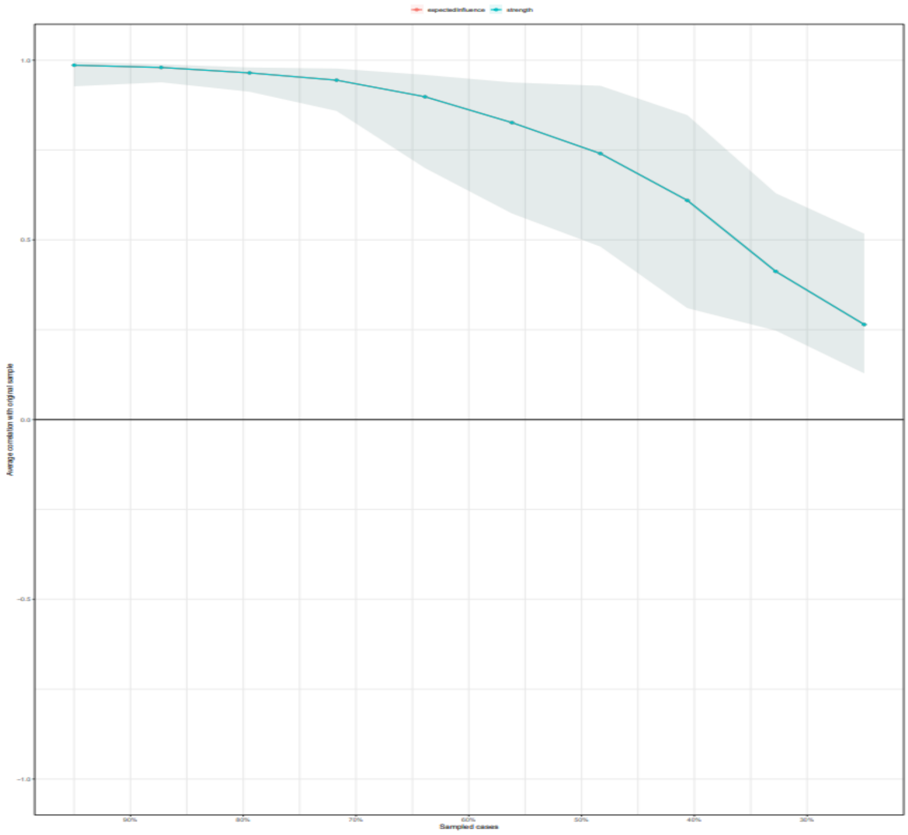

Figure S65 Stability of rural boy network of mental disorder

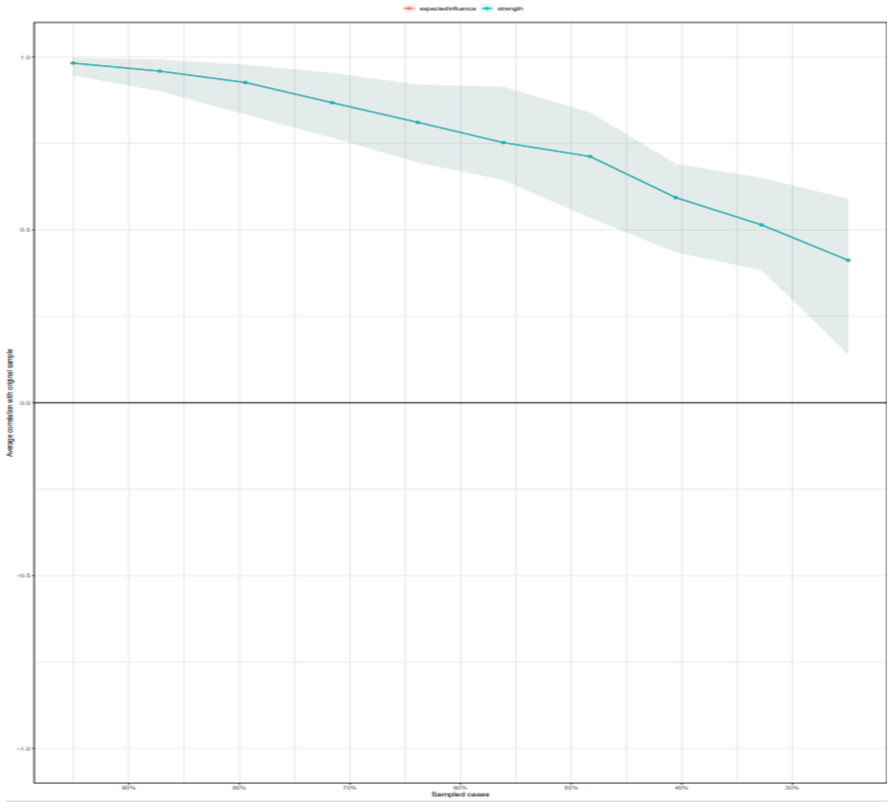

Figure S66 Stability of urban boy of mental disorder

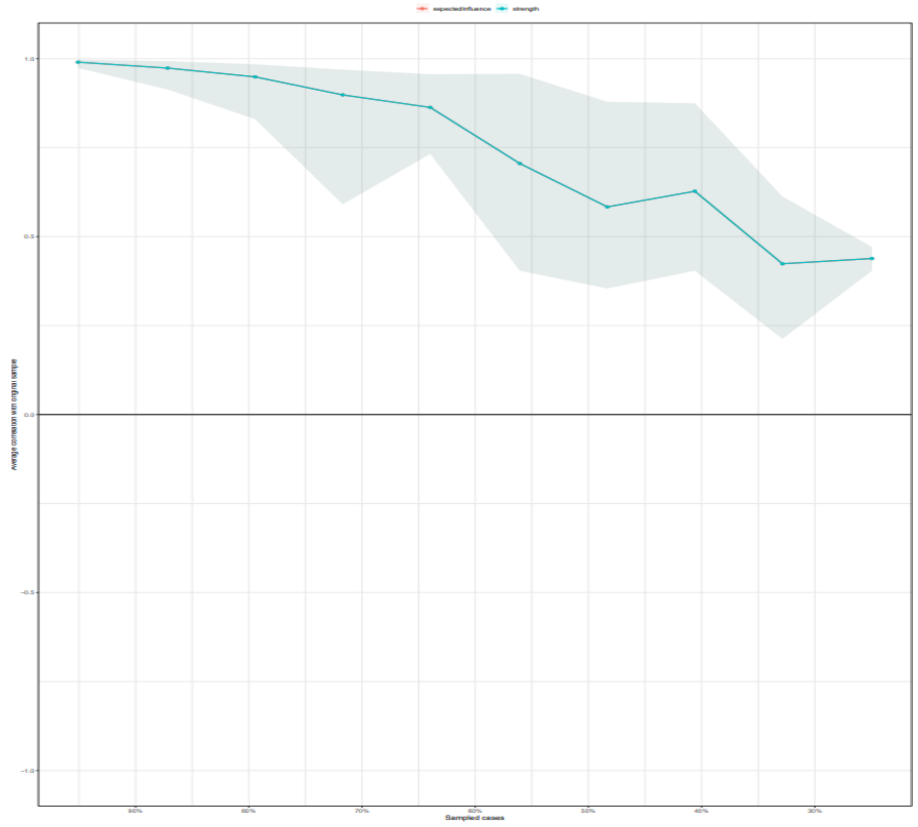

Figure S67 Stability of rural girl network of mental disorder

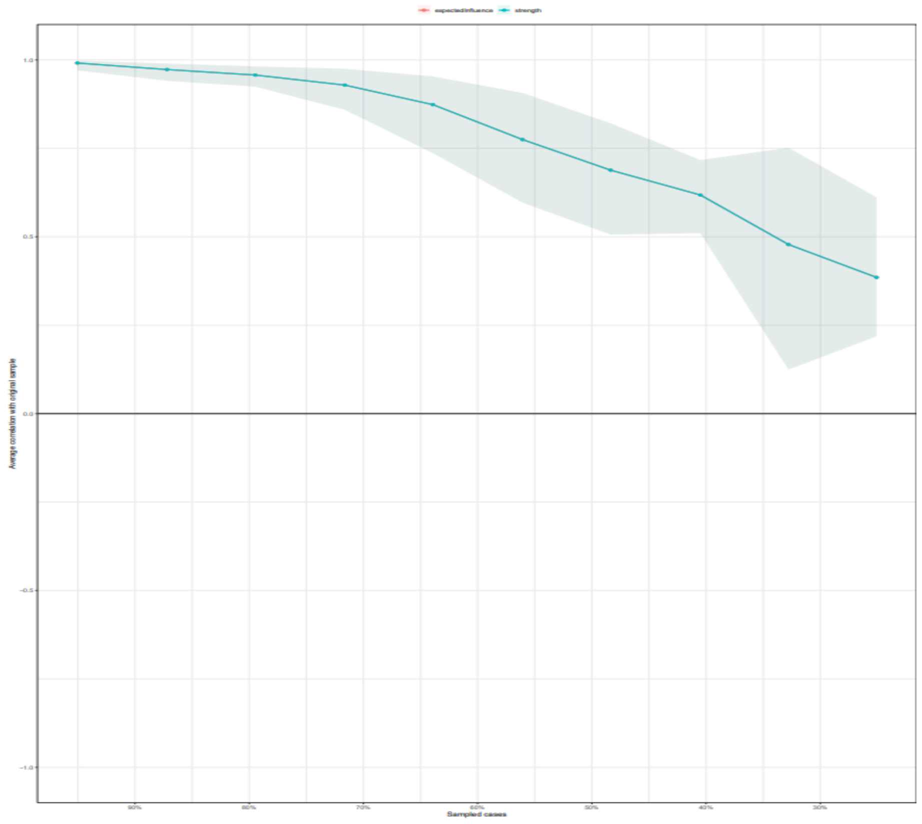

Figure S68 Stability of urban girl of mental disorder

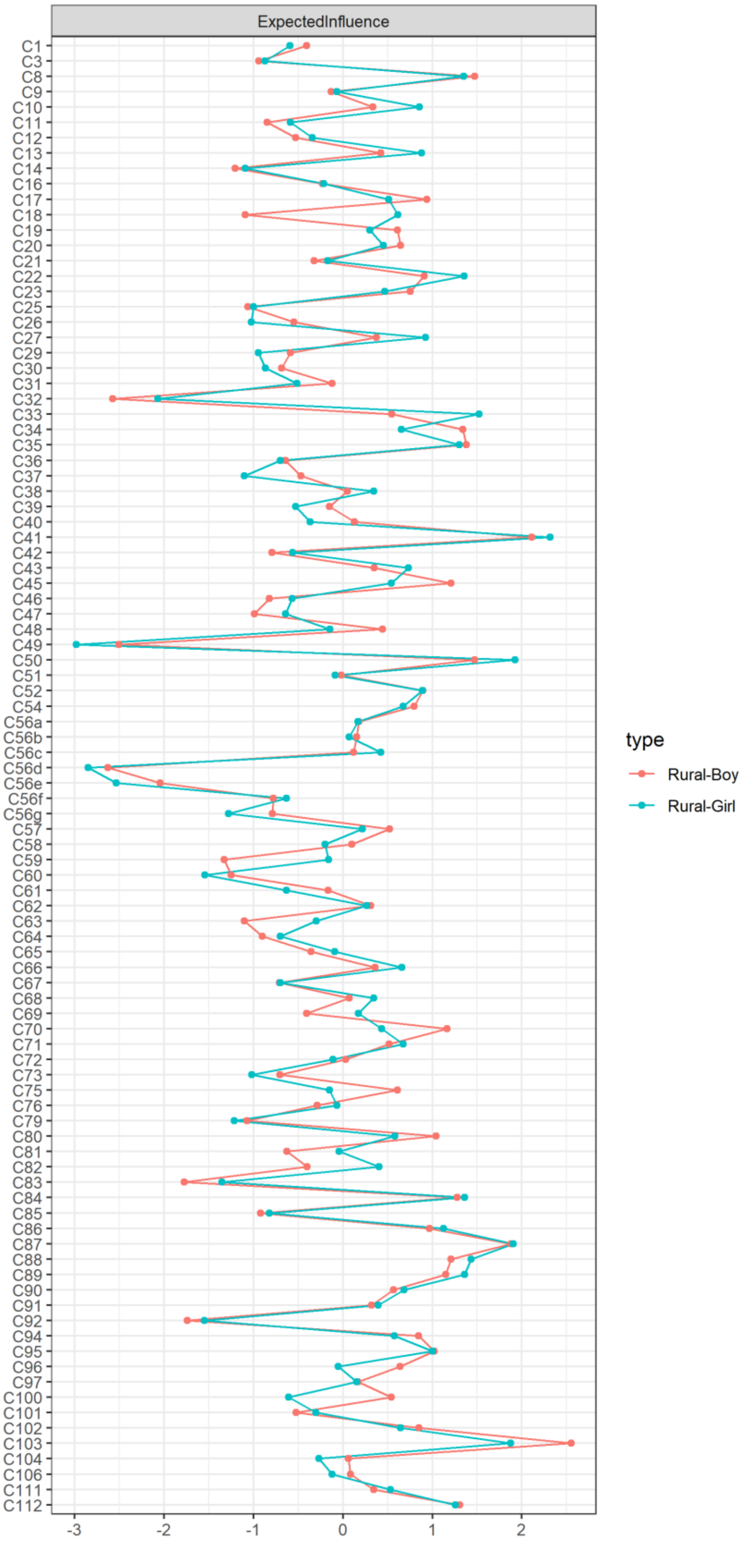

Figure S69 Centrality indicators of rural boy and girls of entire sample

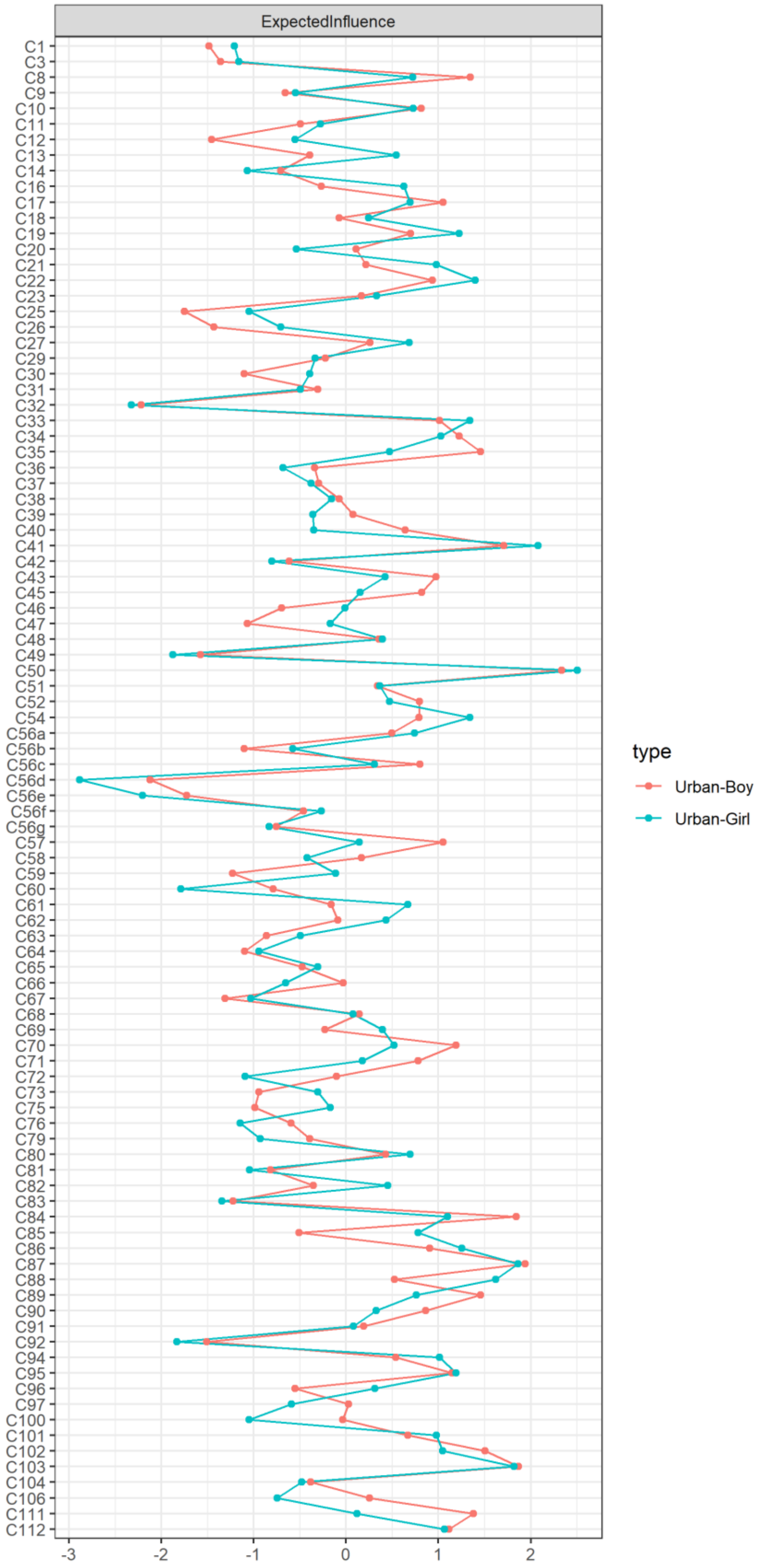

Figure S70 Centrality indicators of urban boy and girls of entire sample

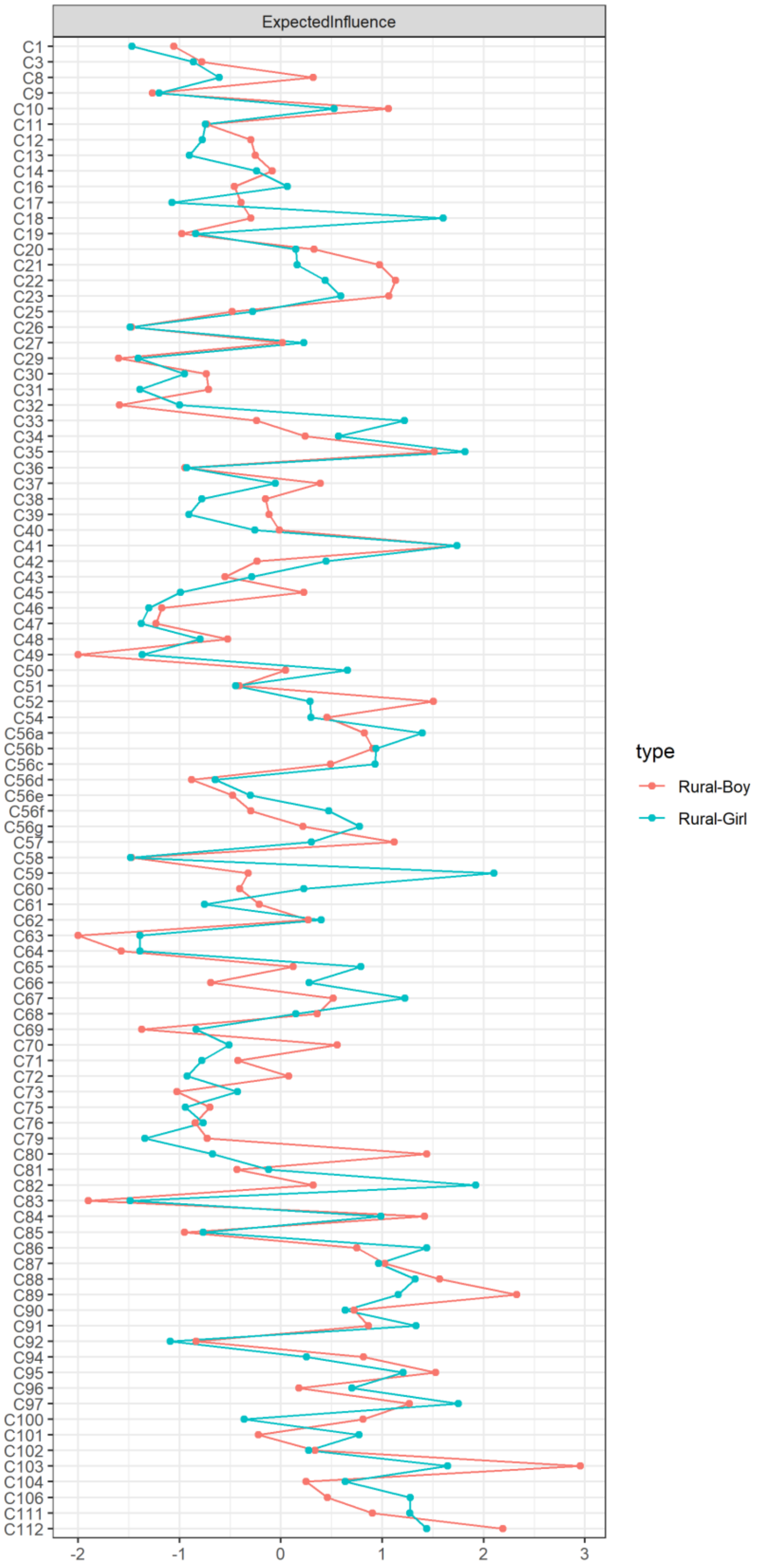

Figure S71 Centrality indicators of rural boy and girls of mental disorder

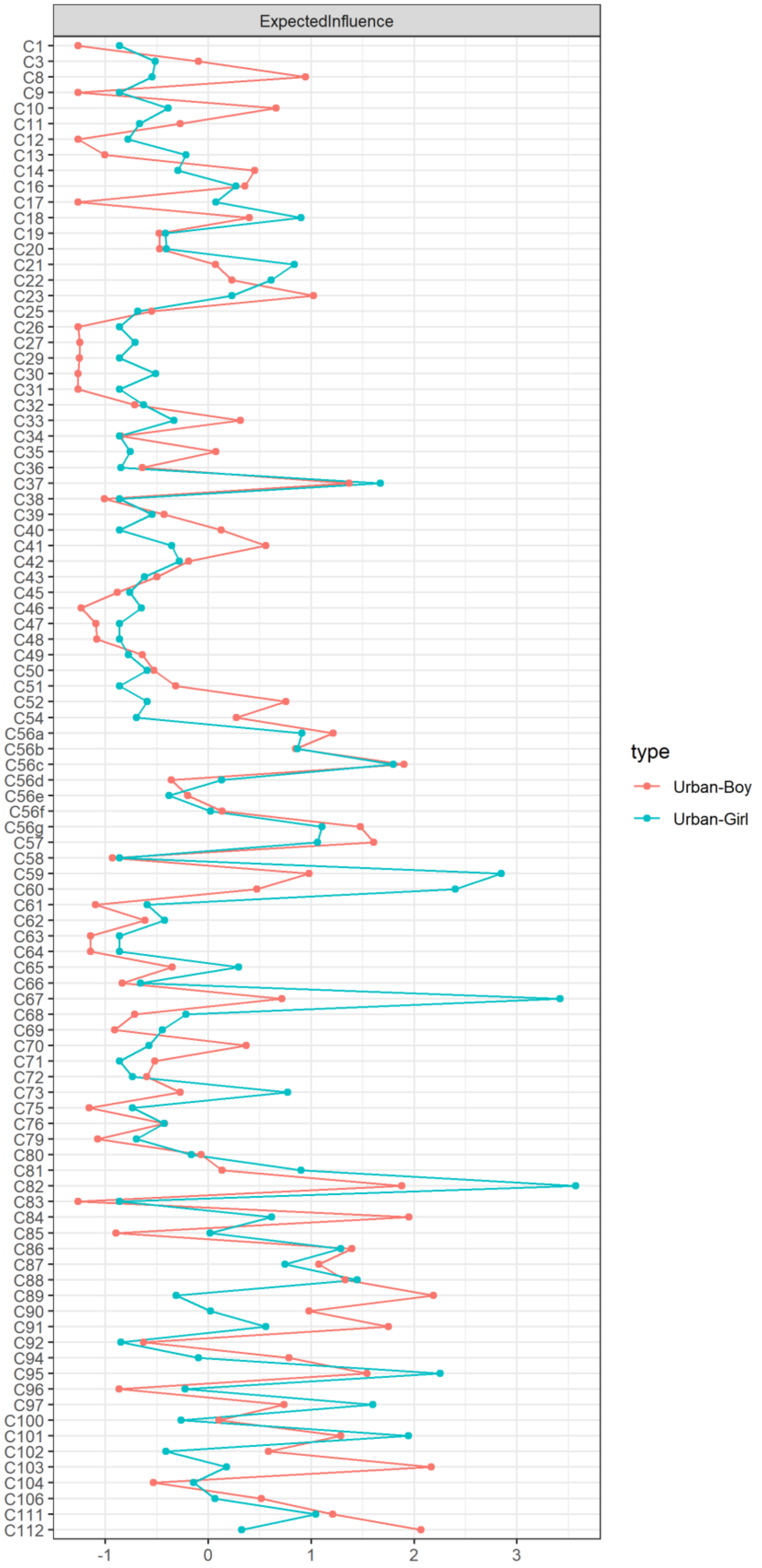

Figure S72 Centrality indicators of urban boy and girls of mental disorder

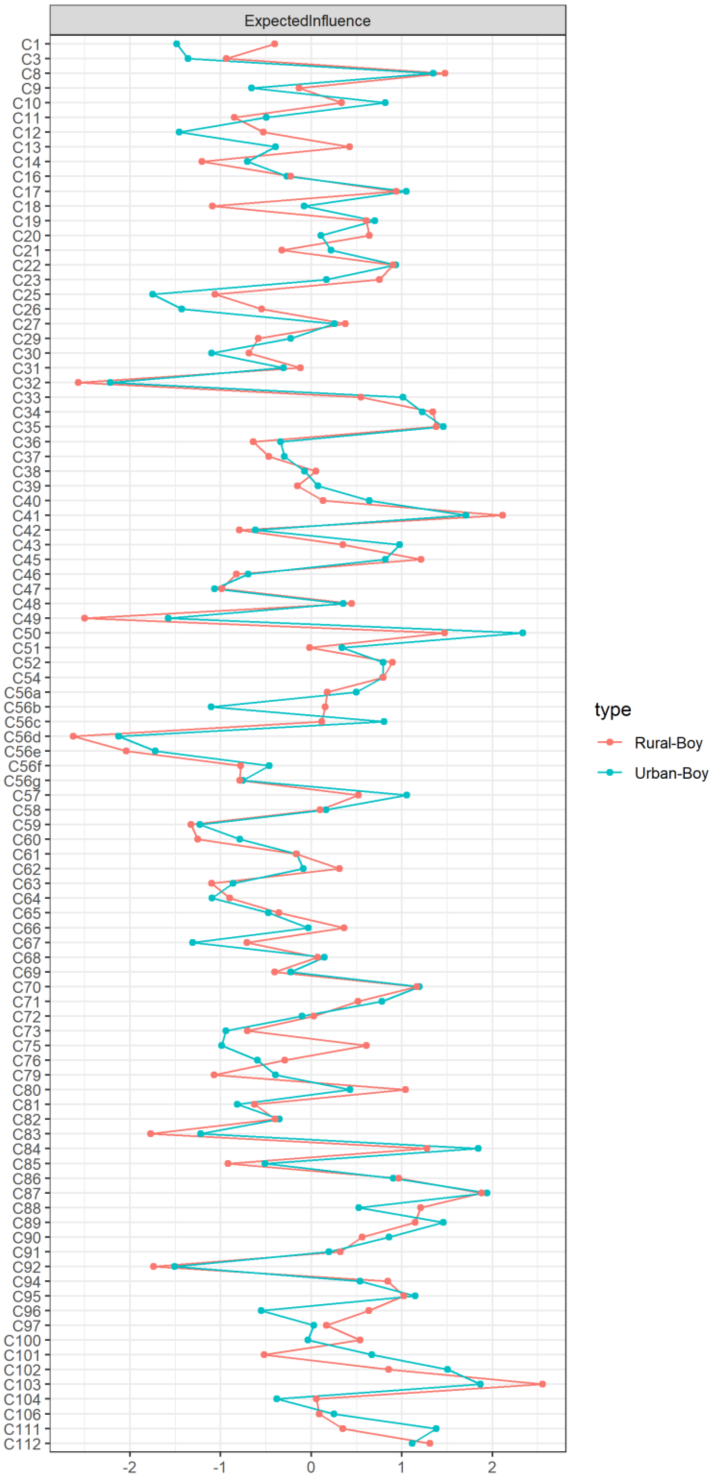

Figure S73 Centrality indicators of rural boy and urban boy of entire sample

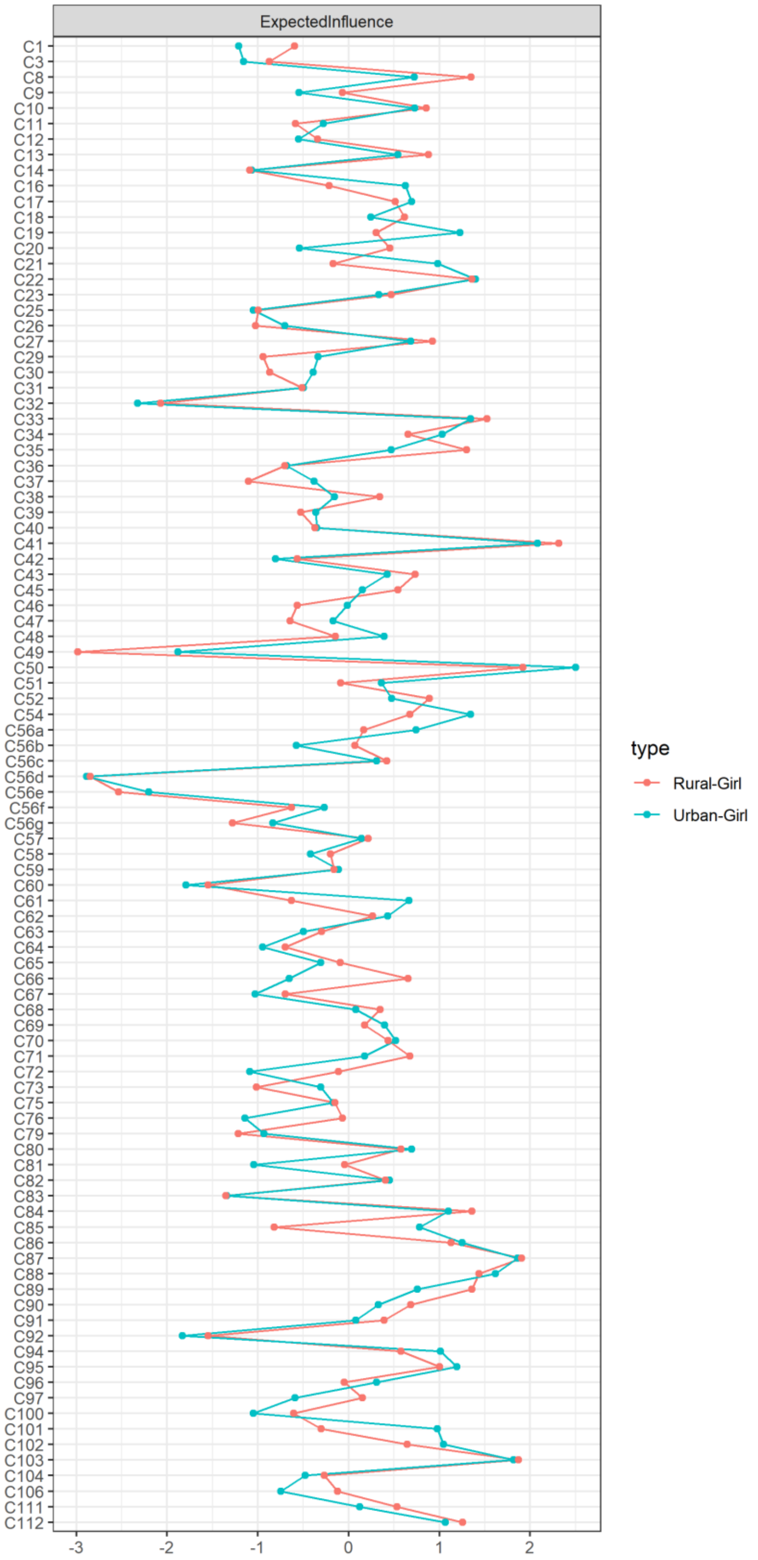

Figure S74 Centrality indicators of rural girl and urban girl of entire sample

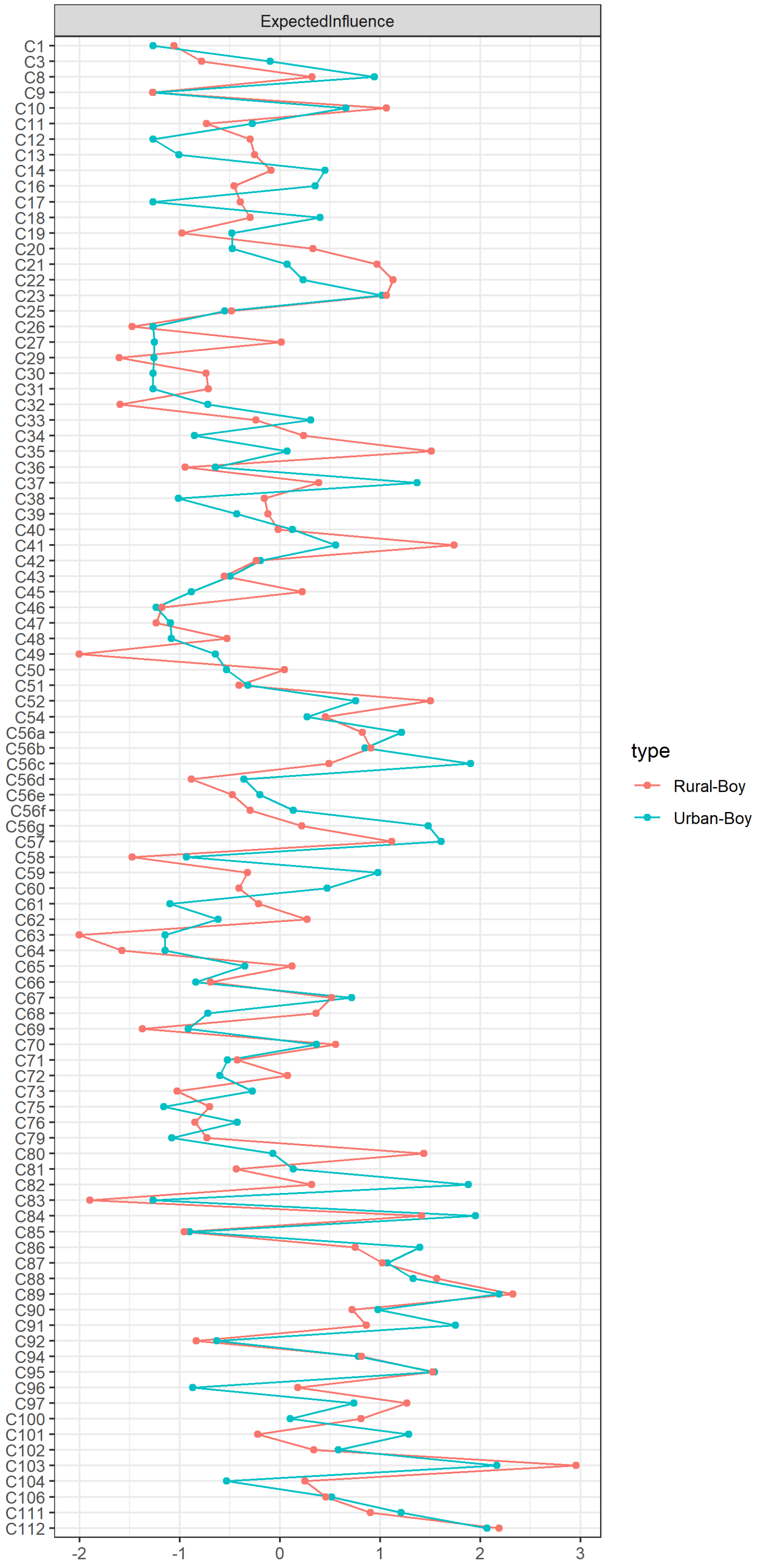

Figure S75 Centrality indicators of rural boy and urban boy of mental disorder

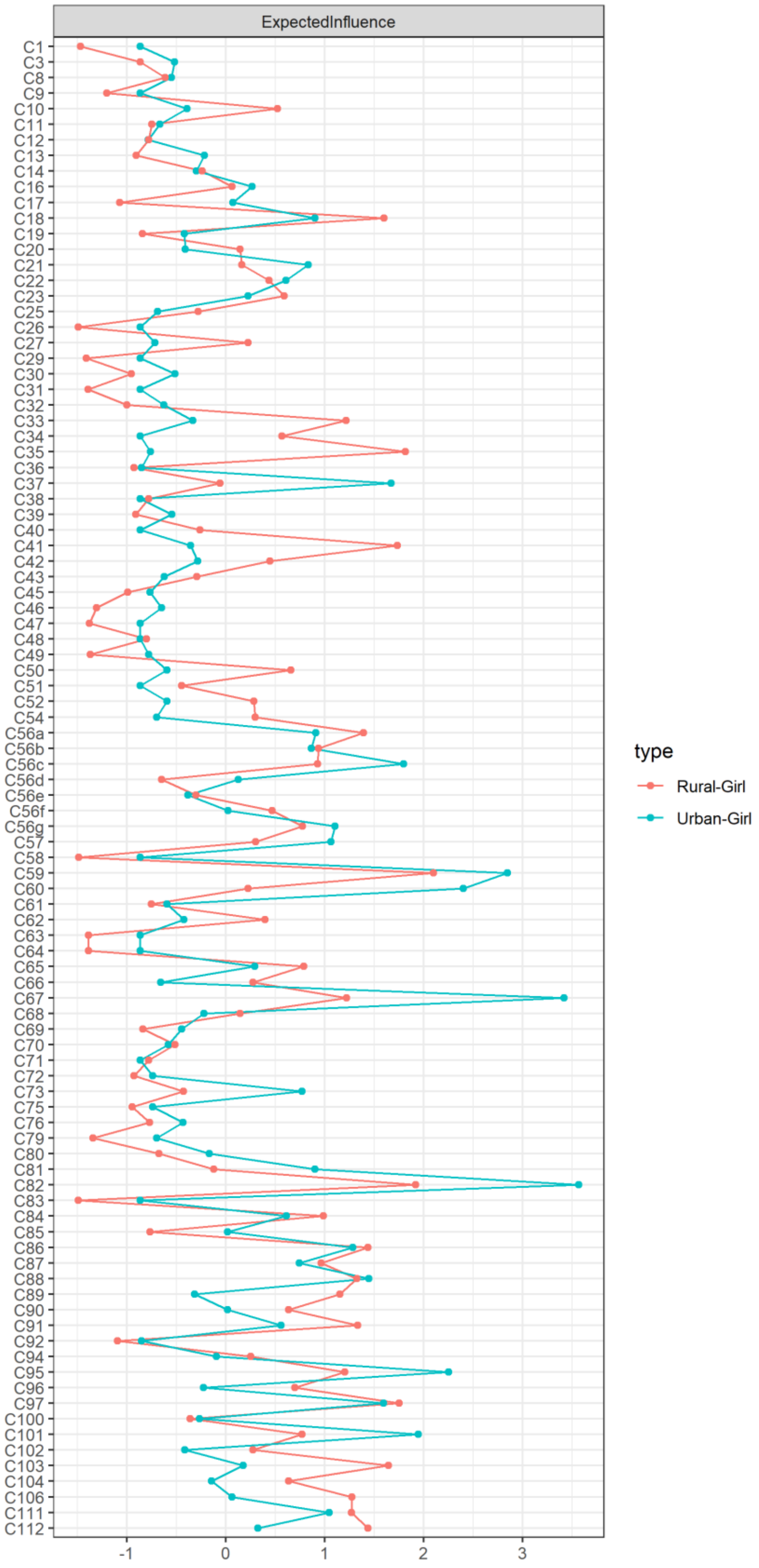

Figure S76 Centrality indicators of rural girl and urban girl of mental disorder
